# Causes of death in endometrial cancer survivors: A Surveillance, Epidemiology, and End Result–based analysis

**DOI:** 10.1002/cam4.5804

**Published:** 2023-03-16

**Authors:** Qing Xue, Wenqiang Che, Lujiadai Xue, Xian Zhang, Xiaoyu Wang, Jun Lyu

**Affiliations:** ^1^ Department of Gynaecology and Obstetrics The First Affiliated Hospital of Jinan University Guangzhou China; ^2^ Department of Clinical Research The First Affiliated Hospital of Jinan University Guangzhou China; ^3^ Department of Neurosurgery The First Affiliated Hospital of Jinan University Guangzhou China

**Keywords:** causes of death, endometrial cancer, non‐endometrial cancer death, standardised mortality ratio (SMR), the Surveillance, Epidemiology, and End Results (SEER) Programme

## Abstract

**Background:**

Increasing attention has been paid to the survival of endometrial cancer (EC) patients, but the non‐cancer causes of death from EC are rarely reported. This study primarily aimed to investigate the non‐cancer causes of death in patients with EC.

**Methods:**

The study collected relevant data, including age, tumour stage and treatment mode, on patients diagnosed with endometrial malignancies from 2000 to 2015 in the Surveillance, Epidemiology, and End Results (SEER) Programme. We analysed the standardised mortality ratio (SMR) to determine the cause of death.

**Results:**

The study included 135,831 patients with EC. During the follow‐up, 46,604 (34.3%) patients died, of whom 42.9%, 15.6% and 41.5% died of EC, other cancers and non‐cancer causes, respectively. As the diagnosis time increased, the number of EC‐associated mortalities gradually decreased. The most common non‐cancer causes of death were heart disease, cerebrovascular disease and diabetes. Regarding the general population of the United States, patients with EC died of heart disease (SMR: 1.06; 95% confidence interval [CI]: 1.03–1.09), diabetes (SMR: 1.56; 95% CI: 1.47–1.65) and septicaemia (SMR: 1.40; 95% CI: 1.28–1.52), which were statistically significant.

**Conclusions:**

For patients with EC, the number of deaths from non‐cancer causes (mainly heart disease, cerebrovascular disease and diabetes mellitus) is equivalent to that of EC. In addition, compared with the general population, EC survivors have a higher risk of death from sepsis and diabetes. These discoveries support how survivors can avoid future‐related health risks. By doing this, clinicians can improve the quality of life and chances of the survival of patients with EC.

## Graphical abstract

We analysed the cause of death of endometrial cancer patients in seer database. During follow‐up, 42.5% of endometrial cancer patients died of non‐cancer causes, which were mainly reflected in heart disease, diabetes, cerebrovascular disease and septicemia.

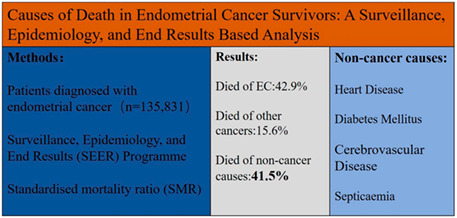

## 1 INTRODUCTION

An estimated 66,570 patients were newly diagnosed with endometrial cancer (EC) in 2021, accounting for 7% of all cancers. Fortunately, the number of deaths from EC is only 12,940, accounting for 4% of all‐cause deaths.^1^ The incidence of EC varies dramatically worldwide, with a higher incidence in North America and Europe than in Africa. However, less regional variation has been observed in mortality rates.^2^

The standard treatment for EC consists of hysterectomy and bilateral salpingo‐oophorectomy.^3^ For patients with EC, adjuvant therapy, including chemotherapy, radiation and targeted therapy, has been identified according to increased risk.^4^ As EC mortality is lower than that of other gynaecological malignancies, patients with EC tend to survive sufficiently.^5^ In other words, the effect of non‐cancer‐related diseases on survival is significant. A previous study on patients with endometrial malignancies in Wisconsin reported that cardiovascular disease and diabetes have a certain impact on the long‐term survival of patients with EC.^6^ Further studies have suggested differences in the survival rates between different races.^6^ A previous study also observed some differences in the mortality risk of non‐cancer causes for breast cancer based on race.^7^ The probability of death from non‐index malignancies varies greatly among primary locations.^8^ The death of patients with cancer caused by non‐cancerous events has gradually attracted attention.^7^, ^8^, ^9^, ^10^ Given the remarkable range of biological characteristics,^11^ treatments^12^ and social factors,^13^ investigating the causes of death in EC survivors with complex health issues is critical. However, there is a dearth of clinical evidence regarding this topic.

In this study, we selected patients diagnosed with EC between 2000 and 2015 using larger sample sizes. We aimed to collect statistics on different demographic and tumour‐related characteristics, which significantly affect the prognosis of EC,^14^ and investigate the associations between these traits and the risk of variant non‐cancer causes of death in EC survivors. We also present a thorough evaluation of changes in the risk of each cause of death over the same period compared with the US general population.

## 2 MATERIALS AND METHODS

We retrieved data from the Surveillance, Epidemiology, and End Results (SEER) Programme using SEER*Stat software version 8.4.0.1.^15^ We selected SEER 17 registers, that covered approximately 26.5% of the general population of the US between 2000 and 2015.^16^ All eligible patients were followed from when they were diagnosed with EC cancer until when they died from various causes or until the end of reporting the information to the SEER programme on 31 December 2015. Approval by the institutional review board was not required since the data in the SEER database were processed anonymously.

We selected patients diagnosed with endometrial malignancy between 2000 and 2015 and followed them until the last day of 2015 or until death. Using the SEER programme, we screened EC data using two keywords: primary site C54.1‐Endometrium and diagnostic by microscopic confirmation. The exclusion criteria were patients with multiple tumours, autopsy or confirmed death.

### 2.1 Outcomes

We mainly discussed the cause of death according to the diagnosis time and divided the diagnosis time into EC diagnoses of <1, 1–5, 5–10 and >10 years. We examined non‐cancer causes of death based on the following variables: age at diagnosis (0–49, 50–64, >64 years), race (Non‐Hispanic White, Non‐Hispanic Black, Non‐Hispanic American Indian/Alaska Native, Non‐Hispanic Asian or Pacific Islander and Hispanic [All Races]), disease stage (using SEER historic stage 2000), N stage (N0, N1, N2, N3, [other unknown or blank]), treatment (surgery, chemotherapy and radiotherapy) and histological subtypes. According to the International Classification of Diseases for Oncology, Third Edition, endometrial carcinoma is classified into endometrioid, non‐endometrioid and sarcoma subtypes. Among them, the endometrioid subtype is 8380; the non‐endometrioid subtype includes 8000, 8010, 8013, 8020, 8041, 8045, 8046, 8050, 8070, 8140, 8246, 8255, 8260, 8310, 8323, 8441, 8460–8461, 8480, 8560, 8570, 8574; and the sarcoma subtype includes 8800, 8805, 8805, 8805, 8930–8931, 8950, 8980.^17^

Since 1999, the causes of death have been supported by the World Health Organization's International Statistical Classification of Diseases and Related Health Problems, 10th Revision.^18^ The causes of death were EC, other cancers and non‐cancers causes. Detailed definitions of each cause of death are presented in Table S1.

### 2.2 Statistical analysis

We measured the standardised mortality ratio (SMR) and the corresponding accuracy (exact method) using SEER*Stat software (8.4.0.1).^15^ We adopted SMRs to compare the variation in the risk of death for each cause among patients with EC and the US general population. The SMR is the observed‐to‐expected ratio, which reflects the strength of the correlation for each cause of death. This observation refers to the number of deaths from EC. Expected refers to the expected number of deaths in the general population. We defined ‘high mortality risk’ as the mortality rate observed in patients with EC being significantly higher than the expected mortality rate for the same cause of death in the general population. The general population refers to a population structure similar to that of cancer patients after adjusting age, race, sex and year.^11^ All statistical tests were bilateral and were statistically significant at *p* < 0.05.

## 3 RESULTS

This study reviewed 135,831 women diagnosed with EC between 2000 and 2015. Patients with EC were overwhelmingly Non‐Hispanic Whites (71.7%). Most patients (85.5%) were aged ≥50 years. Only a few patients with EC were diagnosed with advanced disease (distant, 6.7%) because most endometrial malignancies were found before progression to the advanced stage (localised, 70.0%; regional, 20.3%). The number of married patients (51.1%) was significantly higher than that of single patients (17.8%) and those with marital status at diagnosis is separated, widowed and divorced (SWD) (26.2%). Most patients had a histological type of endometrioid carcinoma (66.1%). Surgery is a common treatment for EC. The surgical rate was 92.7%; radiotherapy (25.8%) and chemotherapy (15.9%) had few applications.

Data analysis revealed that 46,604 (34.3%) patients with EC died during the follow‐up period. One to five years after EC diagnosis, 19,619 (42.1%) patients died. Additionally, 9544 (20.5%), 10,072 (21.6%) and 7369 (15.8%) patients died 1, 5–10 and >10 years after EC diagnosis, respectively. Table 1 shows the characteristics of the study population who died at different time intervals.

**TABLE 1:** Baseline characteristics of all patients with endometrial cancer and patients who died according to the time of death after diagnosis.

Characteristic	Timing of deaths after diagnosis										
		All deaths		<1 year		1–5 years		5–10 years		>10 years	
	Total no. of patients	No. of patients (%)	Mean age of death, years	No. of patients (%)	Mean age of death, years	No. of patients (%)	Mean age of death, years	No. of patients (%)	Mean age of death, years	No. of patients (%)	Mean age of death, years
Overall	135,831	46,604 (100)	73.83	9544 (20.5)	70.16	19,619 (42.1)	71.52	10,072 (21.6)	76.47	7369 (15.8)	81.13
Age at diagnosis, years											
0–49	19,648	2787 (100)	48.5	649 (23.3)	44.17	1093 (39.2)	45.68	560 (20.1)	51.39	485 (17.4)	57.31
50–64	62,362	14,440 (100)	63.75	2801 (19.4)	59.21	6356 (44.0)	61.7	3033 (21.0)	65.96	2250 (15.6)	72.21
>64	53,821	29,377 (100)	81.19	6094 (20.7)	77.97	12,170 (41.4)	78.96	6479 (22.1)	83.55	4634 (15.8)	87.95
Race											
Non‐Hispanic White	97,318	33,671 (100)	75.5	6231 (18.5)	71.78	13,612 (40.4)	73.05	7807 (23.2)	77.8	6021 (17.9)	81.93
Non‐Hispanic Black	11,407	5706 (100)	70.35	1730 (30.3)	68.81	2719 (47.7)	69.15	837 (14.6)	73.01	420 (7.4)	78.97
Non‐Hispanic American Indian/Alaska Native	791	239 (100)	67.5	52 (21.8)	62.19	91 (38.1)	67.01	63 (26.3)	69.05	33 (13.8)	74.24
Non‐Hispanic Asian or Pacific Islander	10,826	2679 (100)	68.36	605 (22.6)	64.69	1245 (46.4)	66.79	498 (18.6)	71.14	331 (12.4)	76.77
Hispanic (all races)	15,489	4309 (100)	69.12	926 (21.5)	65.82	1952 (45.3)	67.35	867 (20.1)	71.43	564 (13.1)	77.11
Disease stage											
Localised	95,162	23,062 (100)	76.62	1970 (8.5)	73.51	8322 (36.1)	73.18	6929 (30.1)	77.38	5841 (25.3)	81.68
Regional	27,609	13,365 (100)	72.39	2669 (20.0)	70.91	6977 (52.2)	70.82	2476 (18.5)	74.85	1243 (9.3)	79.48
Distant	9095	7851 (100)	67.23	4067 (51.8)	66.62	3307 (42.1)	67.27	359 (4.6)	70.82	118 (1.5)	76.36
N stage											
N0	51,814	10,186 (100)	72.2	2147 (21.1)	70.47	5909 (58.0)	71.58	2130 (20.9)	75.68	—	—
N1	3645	1969 (100)	67.75	705 (35.8)	65.99	1101 (55.9)	68.2	163 (8.3)	72.39	—	—
N2	2363	1483 (100)	67.16	549 (37.1)	66.31	837 (56.4)	67.16	97 (6.5)	72.01	—	—
N3	—	—	—	—	—	—	—	—	—	—	—
Other	78,009	32,966 (100)	74.99	6143 (18.6)	70.88	11,772 (35.7)	72.11	7682 (23.3)	76.83	7369 (22.4)	81.13
Tumour grade											
Well‐differentiated; Grade I	49,595	10,449 (100)	75.8	824 (7.9)	71.08	3212 (30.7)	72.24	3353 (32.1)	76.16	3060 (29.3)	80.41
Moderately differentiated; Grade II	34,478	11,347 (100)	75.24	1349 (11.9)	71.79	4409 (38.9)	71.91	3160 (27.9)	76.55	2429 (21.4)	81.5
Poorly differentiated; Grade III	22,361	12,765 (100)	72.81	3578 (28.0)	69.92	6273 (49.1)	71.41	1855 (14.6)	77.55	1059 (8.3)	82.57
Undifferentiated; anaplastic; Grade IV	6779	4272 (100)	70.7	1488 (34.8)	67.91	2160 (50.6)	70.15	437 (10.2)	77.69	187 (4.4)	82.78
Radiotherapy											
Yes	34,999	14,857 (100)	72.88	2310 (15.6)	68.88	7267 (48.9)	70.62	3220 (21.7)	75.69	2060 (13.9)	80.92
Chemotherapy											
Yes	21,646	11,610 (100)	67.3	2867 (24.7)	64.19	6801 (58.6)	67.07	1473 (12.7)	71.35	469 (4.0)	76.72
Surgery											
Yes	125,920	39,222 (100)	73.89	5763 (14.7)	69.05	16,907 (43.1)	70.97	9419 (24.0)	76.54	7133 (18.2)	81.26
Marital status											
Single	24,200	7690 (100)	66.89	1799 (23.4)	63.32	3319 (43.2)	65.14	1574 (20.5)	69.7	998 (13.0)	74.71
Married	69,440	18,966 (100)	72.11	3316 (17.5)	66.92	7904 (41.7)	69.32	4297 (22.7)	74.68	3449 (18.2)	80.32
SWD	35,534	17,620 (100)	78.61	3873 (22.0)	75.75	7393 (42.0)	76.48	3749 (21.3)	81.47	2605 (14.8)	84.81
Type											
Endometrioid subtype	89,798	24,034 (100)	74.52	3249 (13.5)	70.15	9565 (39.8)	71.63	6556 (27.3)	76.28	4664 (19.4)	81.04
Non‐endometrioid subtype	6958	4391 (100)	69.76	1770 (40.3)	68.44	2008 (45.7)	69	404 (9.2)	74.52	209 (4.8)	79.17
Sarcoma subtype	36,510	17,148 (100)	74.04	4238 (24.7)	71.02	7643 (44.6)	72.16	2912 (17.0)	77.31	2355 (13.8)	81.53

*Note*: Values indicate the number of patients with cancer who died.

Abbreviation: SWD: marital status at diagnosis is separated, widowed and divorced.

**FIGURE 1:**
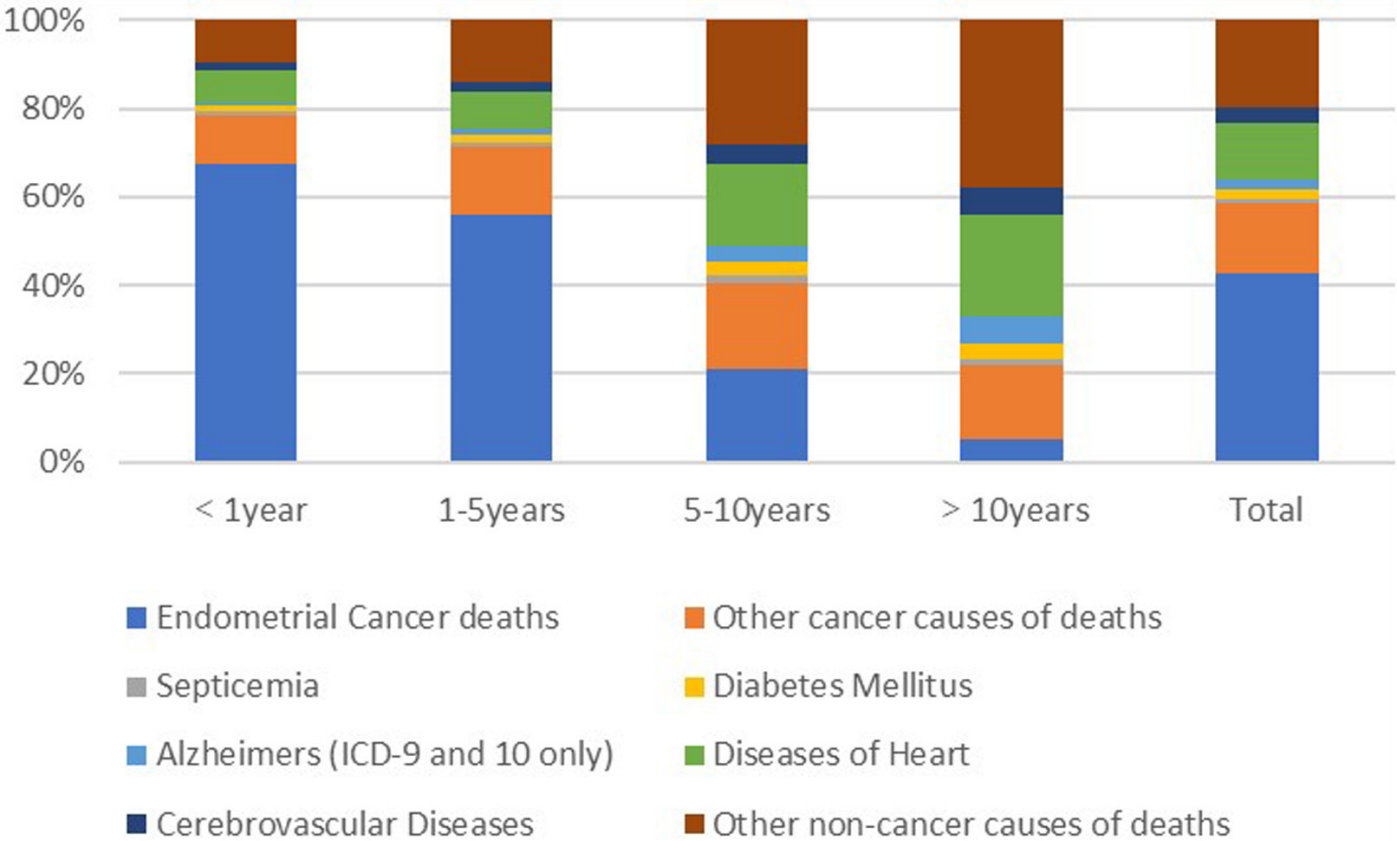
The cause of death during each incubation period after endometrial cancer diagnosis.

During follow‐up, 46,604 patients with EC died, of whom 19,994 (42.9%) died of EC, 7263 (15.6%) died of other cancers and 19,347 (41.5%) died of non‐cancer causes. Among Non‐Hispanic White patients with EC, the number of deaths caused by non‐cancer causes (15,169; 45.1%) was higher than those caused by EC (13,263; 39.3%). Patients older than 64 years died of non‐cancer causes (11,027; 49.2%) more often than from EC (8143; 36.4%). Patients with local disease, N0 Stage, grade I/II tumour differentiation and a pathological subtype of endometrioid carcinoma were more likely to die from non‐cancer causes. Figure 1 shows the cause of death during each incubation period after EC diagnosis. Table 2 shows the characteristics of the study population and the patients with different causes of death.

**TABLE 2:** Baseline characteristics of all patients with endometrial cancer and patients who died according to the causes of death after diagnosis.

Characteristic	No. of patients (%)	No. of deaths (%)	Endometrial cancer deaths	Other cancer causes of deaths	Non‐cancer deaths		
			No. of observed deaths (%)	No. of observed deaths (%)	No. of observed deaths (%)	No. of expected deaths (%)	SMR (95% CI)
Overall	135,831 (100)	46,604 (100)	19,994 (42.9)	7263 (15.6)	19,347 (41.5)	18,413.53	1.05 (1.04–1.07)*
Age at diagnosis, years							
0–49	19,648 (14.5)	2163 (100)	1078 (49.8)	388 (17.9)	697 (32.3)	337.73	2.06 (1.91–2.22)*
50–64	62,362 (45.9)	11,226 (100)	5529 (49.2)	2008 (17.9)	3689 (32.9)	3027.15	1.22 (1.18–1.26)*
>64	53,821 (39.6)	22,401 (100)	8143 (36.4)	3231 (14.4)	11,027 (49.2)	11,974.15	0.92 (0.9–0.94)*
Race							
Non‐Hispanic White	97,318 (71.7)	33,671 (100)	13,263 (39.3)	5239 (15.6)	15,169 (45.1)	15,282.79	0.99 (0.98–1.01)
Non‐Hispanic Black	11,407 (8.3)	5706 (100)	3147 (55.1)	876 (15.4)	1683 (29.5)	1218.65	1.38 (1.32–1.45)*
Non‐Hispanic American Indian/Alaska Native	860 (0.6)	239 (100)	87 (36.4)	45 (18.8)	107 (44.8)	35.26	3.03 (2.49–3.67)*
Non‐Hispanic Asian or Pacific Islander	10,826 (8.0)	2679 (100)	1435 (53.6)	387 (14.4)	857 (32.0)	563.09	1.52 (1.42–1.63)*
Hispanic (all races)	15,489 (11.4)	4309 (100)	2099 (48.7)	723 (16.8)	1487 (34.5)	1313.74	1.13 (1.08–1.19)*
Disease stage							
Localised	95,162 (70.0)	19,233 (100)	4228 (22.0)	3412 (17.7)	11,593 (60.3)	12,523.61	0.93 (0.91–0.94)*
Regional	27,609 (20.3)	10,855 (100)	6112 (56.3)	1418 (13.1)	3325 (30.6)	2562.34	1.30 (1.25–1.34)*
Distant	9095 (6.7)	5702 (100)	4410 (77.3)	797 (14.0)	495 (8.7)	253.09	1.96 (1.79–2.14)*
N stage							
N0	51,814 (38.2)	10,186 (100)	5046 (49.5)	1547 (15.2)	3593 (35.3)	3738.48	0.96 (0.93–0.99)*
N1	3645 (2.7)	1969 (100)	1483 (75.3)	237 (12.0)	249 (12.7)	182.24	1.37 (1.2–1.55)*
N2	2363 (1.7)	1483 (100)	1148 (77.4)	151 (10.2)	184 (12.4)	95.77	1.92 (1.65–2.22)*
N3	—	—	—	—	—	—	—
Other	78,009 (57.4)	32,966 (100)	12,354 (37.5)	5335 (16.2)	15,277 (46.3)	14,397.04	1.06 (1.04–1.08)*
Tumour grade							
Well‐differentiated; Grade I	49,595 (36.5)	9416 (100)	1595 (16.9)	1713 (18.2)	6108 (64.9)	6582.99	0.93 (0.90–0.95)*
Moderately differentiated; Grade II	34,478 (25.4)	10,513 (100)	3470 (33.0)	1689 (16.1)	5354 (50.9)	5358.41	1 (0.97–1.03)
Poorly differentiated; Grade III	22,361 (16.5)	11,869 (100)	7001 (59.0)	1688 (14.2)	3180 (26.8)	2771.85	1.15 (1.11–1.19)*
Undifferentiated; anaplastic; Grade IV	6779 (5.0)	3992 (100)	2684 (67.2)	537 (13.5)	771 (19.3)	625.79	1.23 (1.15–1.32)*
Radiotherapy							
Yes	34,999 (25.8)	11,850 (100)	5923 (50.0)	1676 (14.1)	4251 (35.9)	4052.48	1.05 (1.02–1.08)*
Chemotherapy							
Yes	21,646 (15.9)	8721 (100)	6220 (71.3)	1262 (14.5)	1239 (14.2)	1138.38	1.09 (1.03–1.15)*
Surgery							
Yes	125,920 (92.7)	32,195 (100)	12,556 (39.0)	5295 (16.4)	14,344 (44.6)	14,996.90	0.96 (0.94–0.97)*
Marital status							
Single	24,200 (17.8)	6111 (100)	2796 (45.8)	908 (14.9)	2407 (39.4)	1630.58	1.48 (1.42–1.54)*
Married	69,440 (51.1)	15,571 (100)	6607 (42.4)	2665 (17.1)	6299 (40.5)	7431.12	0.85 (0.83–0.87)*
SWD	35,534 (26.2)	14,108 (100)	5347 (37.9)	2054 (14.6)	6707 (47.5)	6277.34	1.07 (1.04–1.09)*
Type							
Endometrioid subtype	89,798 (66.1)	19,644 (100)	6366 (32.4)	3145 (16.0)	10,133 (51.6)	10,377.40	0.98 (0.96–1)*
Non‐endometrioid subtype	6958 (5.1)	2759 (100)	1879 (68.1)	418 (15.2)	462 (16.7)	357.91	1.29 (1.18–1.41)*
Sarcoma subtype	36,510 (26.9)	12,752 (100)	6207 (48.7)	1956 (15.3)	4589 (36.0)	4366.89	1.05 (1.02–1.08)*

*Note*: Values indicate the number of patients with cancer who died from each cause of death.

Abbreviations: CI, confidence interval; SMR, standardised mortality ratio; SWD, marital status at diagnosis is separated, widowed and divorced.

* *p* < 0.05.

**FIGURE 2:**
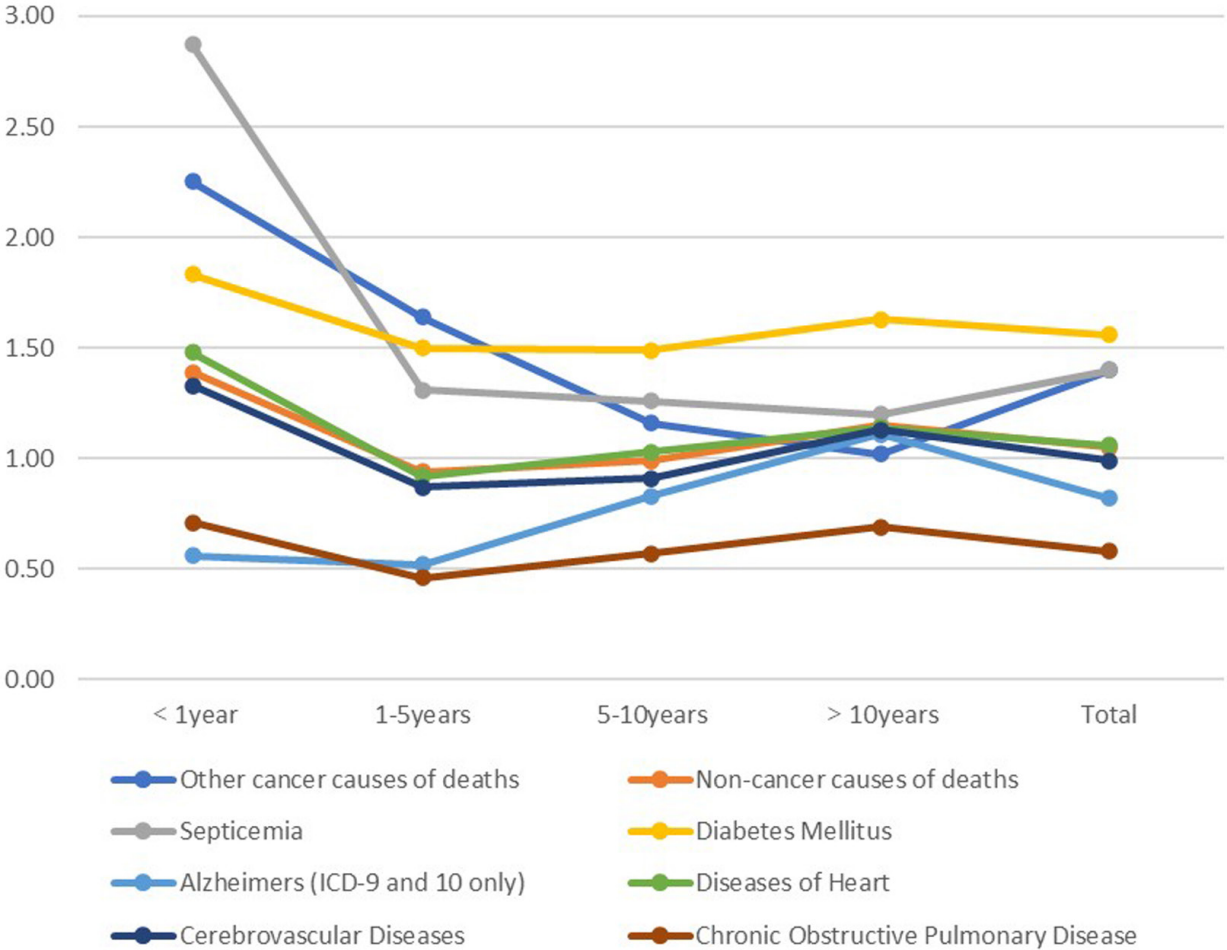
The changes in standardised mortality ratio in endometrial cancer survivors with different causes of death and incubation periods.

The leading cause of non‐cancer death was heart disease (5950, 12.8%), followed by cerebrovascular disease (1525, 3.3%), diabetes mellitus (1066, 2.3%) and Alzheimer's disease (1036, 2.2%). However, using the US general population as a reference, EC survivors have an increased risk of death from diabetes at any time after diagnosis. The SMR for sepsis was highest in the first year after EC diagnosis. Diabetes SMR first occurred after the diagnosis time was extended. The SMR of Alzheimer's increases with increasing diagnosis time. However, the risk of death due to chronic obstructive pulmonary disease (COPD) was lower than that in the general population. Figure 2 shows the changes in SMR in EC patients with different causes of death and incubation periods. Table 3 shows the death events observed at different time intervals and SMR for each cause of death.

**TABLE 3:** Standardised mortality ratios for each cause of death after endometrial cancer diagnosis.

	Timing of deaths after diagnosis									
	<1 year		1–5 years		5–10 years		>10 years		Total	
Causes of death	No. observed	SMR (95% CI)	No. observed	SMR (95% CI)	No. observed	SMR (95% CI)	No. observed	SMR (95% CI)	No. observed	SMR (95% CI)
All causes of death	9544	4.87 (4.78–4.97)*	19,619	2.51 (2.47–2.54)*	10,072	1.29 (1.27–1.32)*	7369	1.18 (1.15–1.2)*	46,604	1.95 (1.94–1.97)*
Endometrial cancer deaths	6438	299.59 (292.32–307)*	11,020	128.22 (125.84–130.64)*	2140	27.51 (26.36–28.7)*	396	7.39 (6.68–8.16)*	19,994	83.73 (82.57–84.9)*
Other cancer causes of deaths	1066	2.25 (2.12–2.39)*	3040	1.64 (1.59–1.7)*	1950	1.16 (1.11–1.21)*	1207	1.02 (0.96–1.08)	7263	1.40 (1.37–1.43)*
Non‐cancer causes of deaths	2040	1.39 (1.33–1.46)*	5559	0.94 (0.92–0.97)*	5982	0.99 (0.97–1.02)	5766	1.15 (1.12–1.18)*	19,347	1.05 (1.04–1.07)*
In situ, benign or unknown behaviour neoplasm	30	2.65 (1.79–3.78)*	72	1.59 (1.24–2)*	54	1.19 (0.89–1.55)	49	1.35 (1–1.79)*	205	1.48 (1.29–1.7)*
Septicemia	90	2.87 (2.31–3.53)*	163	1.31 (1.11–1.52)*	153	1.26 (1.07–1.48)*	111	1.2 (0.98–1.44)	517	1.40 (1.28–1.52)*
Other infectious and parasitic diseases including HIV	26	1.57 (1.03–2.3)*	76	1.15 (0.91–1.44)	76	1.23 (0.97–1.54)	56	1.31 (0.99–1.7)	234	1.25 (1.09–1.42)*
Diabetes mellitus	115	1.83 (1.51–2.2)*	360	1.50 (1.35–1.66)*	328	1.49 (1.33–1.66)*	263	1.63 (1.44–1.84)*	1066	1.56 (1.47–1.65)*
Alzheimer's	42	0.56 (0.41–0.76)*	177	0.52 (0.45–0.6)*	347	0.83 (0.75–0.92)*	470	1.11 (1.01–1.21)*	1036	0.82 (0.78–0.88)*
Diseases of heart	709	1.48 (1.37–1.59)*	1706	0.92 (0.88–0.97)*	1863	1.03 (0.98–1.07)	1672	1.14 (1.08–1.19)*	5950	1.06 (1.03–1.09)*
Hypertension without heart disease	37	1.62 (1.14–2.23)*	111	1.15 (0.95–1.39)	128	1.22 (1.02–1.45)*	135	1.46 (1.23–1.73)*	411	1.30 (1.18–1.43)*
Cerebrovascular diseases	172	1.33 (1.14–1.55)*	433	0.87 (0.79–0.96)*	449	0.91 (0.82–0.99)*	471	1.13 (1.03–1.23)*	1525	0.99 (0.94–1.04)
Atherosclerosis	10	1.23 (0.59–2.26)	30	1.07 (0.72–1.53)	22	0.92 (0.58–1.39)	27	1.57 (1.03–2.28)*	89	1.15 (0.93–1.42)
Aortic aneurysm and dissection	3	0.36 (0.07–1.06)	17	0.55 (0.32–0.88)*	10	0.36 (0.17–0.66)*	15	0.73 (0.41–1.21)	45	0.52 (0.38–0.69)*
Other diseases of arteries, arterioles, capillaries	12	1.47 (0.76–2.57)	33	1.04 (0.72–1.46)	26	0.84 (0.55–1.23)	19	0.76 (0.46–1.19)	90	0.94 (0.76–1.16)
Pneumonia and influenza	50	1.09 (0.81–1.44)	157	0.88 (0.75–1.03)	187	1.07 (0.93–1.24)	147	1.05 (0.89–1.24)	541	1.01 (0.92–1.09)
Chronic obstructive pulmonary disease	90	0.71 (0.57–0.88)*	244	0.46 (0.41–0.53)*	306	0.57 (0.51–0.64)*	292	0.69 (0.61–0.77)*	932	0.58 (0.54–0.62)*
Stomach and duodenal ulcers	5	1.8 (0.58–4.2)	20	1.92 (1.17–2.97)*	12	1.24 (0.64–2.17)	11	1.46 (0.73–2.61)	48	1.58 (1.16–2.09)*
Chronic liver disease and cirrhosis	28	1.46 (0.97–2.11)	80	1.04 (0.82–1.29)	69	1.02 (0.79–1.29)	48	1.07 (0.79–1.42)	225	1.08 (0.94–1.23)
Nephritis, nephrotic syndrome and nephrosis	59	1.59 (1.21–2.05)*	181	1.21 (1.04–1.41)*	179	1.20 (1.03–1.39)*	178	1.53 (1.31–1.77)*	597	1.32 (1.22–1.43)*
Complications of pregnancy, childbirth, puerperium	1	6.75 (0.17–37.64)	4	8.11 (2.21–20.77)*	1	3.57 (0.09–19.88)	0	0 (0–38.96)	6	5.90 (2.17–12.85)*
Congenital anomalies	4	1.64 (0.45–4.19)	5	0.53 (0.17–1.25)	7	0.9 (0.36–1.86)	6	1.2 (0.44–2.6)	22	0.9 (0.56–1.36)
Certain conditions originating in perinatal period	0	0 (0–528.23)	0	0 (0–134.67)	0	0 (0–154.43)	0	0 (0–239.68)	0	0 (0–50.08)
Symptoms, signs and ill‐defined conditions	34	1.44 (1–2.02)	104	1.09 (0.89–1.32)	98	0.94 (0.76–1.14)	67	0.84 (0.65–1.07)	303	1 (0.89–1.12)
Accidents and adverse effects	45	0.92 (0.67–1.23)	154	0.76 (0.65–0.89)*	157	0.78 (0.66–0.91)*	138	0.85 (0.71–1)	494	0.80 (0.73–0.88)*
Suicide and self‐inflicted injury	8	1.03 (0.44–2.02)	16	0.53 (0.3–0.86)*	20	0.85 (0.52–1.31)	15	1.14 (0.64–1.88)	59	0.79 (0.6–1.02)
Homicide and legal intervention	1	0.48 (0.01–2.68)	8	1.07 (0.46–2.11)	3	0.53 (0.11–1.55)	3	0.93 (0.19–2.7)	15	0.81 (0.46–1.34)
Other causes of death	372	1.27 (1.14–1.4)*	1122	0.89 (0.84–0.95)*	1288	0.93 (0.88–0.98)*	1349	1.10 (1.04–1.16)*	4131	0.99 (0.96–1.02)

*Note*: Values indicate the number of patients with cancer who died from each cause of death.

Abbreviations: CI, confidence interval; SMR, standardised mortality ratio.

* *p* < 0.05.

### 3.1 Non‐cancer causes of death within 1 year after EC diagnosis

A total of 9544 patients died in the first year after EC diagnosis, of whom 6438 (67.5%) died from EC, 1066 (11.2%) from other cancers and 2040 (21.4%) from non‐cancer causes. During this period, the most common cause of non‐cancer death was heart disease (709, 34.8%), followed by cerebrovascular disease (172, 8.4%), and diabetes mellitus (115, 5.6%).

Compared with the US general population, EC survivors had a significantly higher risk of death due to non‐cancer comorbidities, reflected by sepsis (SMR: 2.87; 95% CI: 2.31–3.53), diabetes (SMR: 1.83; 95% CI: 1.51–2.2), heart disease (SMR: 1.48; 95% CI: 1.37–1.59), cerebrovascular disease (SMR: 1.33; 95% CI: 1.14–1.55) and hypertension without heart disease (SMR: 1.62; 95% CI: 1.14–2.23) (Table 3). EC survivors who were Non‐Hispanic Black and Non‐Hispanic Asian or Pacific Islander, in the distant, N1/N2 stage, received chemotherapy, and sarcoma subtypes had a significantly higher risk of death from sepsis within the first year than the general population (see Supporting Tables S6, S8, S12, S14, S15, S21 and S28). EC survivors <49 years of age had a higher risk of sepsis and heart disease within the first year after diagnosis. Survivors over 49 years of age showed more deaths due to heart disease and diabetes than due to septicaemia (Tables S2–S4). Survivors who were non‐Hispanic American Indian/Alaska Natives had a significantly higher risk of death from diabetes. The risks of death associated with Alzheimer's disease (SMR: 0.56; 95% CI: 0.41–0.76) and COPD (SMR: 0.71; 95% CI: 0.57–0.88) in patients with EC were significantly lower than those in the general population (Table 3).

In general, the causes of death in the first year after EC diagnosis followed a trend similar to that of the general population, with sepsis and cardio‐cerebrovascular diseases being the most common causes of death in specific demographic and tumour‐related subgroups (Tables S2–S24).

### 3.2 Non‐cancer causes of death within 1–5 years after EC diagnosis

Approximately 19,619 patients died within 1–5 years of EC diagnosis, of whom 11,020 (56.2%) died from EC, 3040 (15.5%) from other cancers and 5559 (28.3%) from non‐cancer causes. Among deaths from non‐cancer causes, the most common cause of death was heart disease (1706, 30.7%), followed by cerebrovascular disease (433, 7.8%), diabetes mellitus (360, 6.5%) and COPD (244, 4.4%).

The risk of death from septicaemia (SMR: 1.31; 95% CI: 1.11–1.52), diabetes mellitus (SMR: 1.50; 95% CI: 1.35–1.66), stomach and duodenal ulcers (SMR: 1.92; 95% CI: 1.17–2.97) and nephritis, nephrotic syndrome and nephrosis (SMR: 1.21; 95% CI: 1.04–1.41) were statistically significantly higher than in the US general population (referenced to Table 3), while the risk of death from Alzheimer's disease (SMR: 0.52; 95% CI: 0.45–0.6), heart disease (SMR: 0.92; 95% CI: 0.88–0.97) and cerebrovascular disease (SMR: 0.87; 95% CI: 0.79–0.96) was statistically lower. Patients under 64 years had a significantly increased risk of death from heart disease and sepsis within 1–5 years of being diagnosed with EC (Table 2 and Tables S2 and S3). Non‐Hispanic American Indian/Alaska Native, endometrioid carcinoma types, unmarried status, non‐distant stage, N0‐stage and non‐tumour grade IV with EC were significantly associated with an increased risk of death from diabetes mellitus (see Tables S7, S10 and S19–S28). Patients receiving radiotherapy were at an increased risk of death from sepsis, diabetes, nephritis, nephrotic syndrome and nephrosis. It is worth mentioning that patients with locally diagnosed EC had a lower risk of non‐cancerous death within 1–5 years of diagnosis than the general population (Table S9).

The trends in mortality in the other subgroups of patients were comparable to those in the general population (Table 3 and all Supporting Tables).

### 3.3 Non‐cancer causes of death within 5–10 years after EC diagnosis

A total of 10,072 patients died within 5–10 years of EC diagnosis. Among them, 2140 (21.2%) died of EC, 1950 (19.4%) died of other cancers and 5982 (59.4%) died of non‐cancer causes. Heart disease (1863, 31.1%), cerebrovascular disease (449, 7.5%), Alzheimer's disease (347, 5.8%), diabetes mellitus (328, 5.5%) and COPD (306, 5.1%) were the common causes of non‐cancer deaths.

Compared with the US general population, the risk of death from COPD (SMR: 0.57; 95% CI: 0.51–0.64) was lower. The risk of death from septicaemia (SMR: 1.26; 95% CI: 1.07–1.48), diabetes mellitus (SMR: 1.49; 95% CI: 1.33–1.66) and hypertension without heart disease (SMR: 1.22; 95% CI: 1.02–1.45) was statistically significantly higher (Table 3). Survivors of the Hispanic, regional stage and unmarried status were at significantly higher risk of death from hypertension without heart disease (Tables S9, S11 and S23–S25). Patients under 65 years at diagnosis, Non‐Hispanic White, non‐localised stage, N0 or N2 stage, had a significantly increased risk of dying from septicaemia (see Tables S2, S3, S5 and S11–S15). Patients receiving radiotherapy were at a significantly higher risk of death from septicaemia, other infectious and parasitic diseases and diabetes mellitus (Table S20). In addition, patients receiving chemotherapy had a significantly higher risk of death from septicaemia (Table S21). The mortality trends in other subgroups of patients were similar to those in the general population (Table 3 and all Supporting Tables).

### 3.4 Non‐cancer causes of death >10 years after EC diagnosis

A total of 7369 patients died >10 years after EC diagnosis. Of these, 396 (5.4%) died from EC, 1207 (12.4%) from other cancers and 5766 (78.2%) from non‐cancer causes. The most common cause of death among non‐cancer patients was heart disease (1672, 29.0%), followed by Alzheimer's disease (470, 8.2%), cerebrovascular disease (471, 8.2%), COPD (292, 5.1%) and diabetes mellitus (263, 4.6%).

Women with EC had a statistically significantly higher risk of partial non‐cancer causes of death more than 10 years after the cancer diagnosis compared with the US general population, mainly reflected by diabetes mellitus (SMR: 1.63; 95% CI: 1.44–1.84), Alzheimer's (SMR: 1.11; 95% CI: 1.01–1.21), heart diseases (SMR: 1.14; 95% CI: 1.08–1.19), hypertension without heart disease (SMR: 1.46; 95% CI: 1.23–1.73), cerebrovascular diseases (SMR: 1.13; 95% CI: 1.03–1.23) and atherosclerosis (SMR: 1.57; 95% CI: 1.03–2.28) (Table 3). Survivors over 65 years, Non‐Hispanic White, who had surgery, and who had SWD status, had a significantly higher risk of dying from Alzheimer's disease (Tables S4, S5, S22 and S25). Survivors under 65 years, Non‐Hispanic, non‐distant stage, non‐grade IV, received radiotherapy, underwent surgery, unmarried status and had an endometrioid subtype were at significantly higher risk of death from heart diseases (Tables S2, S3, S5–S8, S10, S11, S16–S18, S20 and S22–S26). Survivors who were Non‐Hispanic White, non‐distant stage, received treatment, had SWD status and had an endometrioid subtype were at significantly higher risk of death from hypertension without heart disease (Tables S5, S10, S11, S20–S22, S25 and S26). Survivors who were at the regional stage, Non‐Hispanic Asian or Pacific Islander, Hispanic, grade III; underwent surgery; and had SWD status; and non‐endometrioid subtype was at a significantly higher risk of death from cerebrovascular diseases (Tables S8, S9, S11, S18, S22, S25 and S27). Trends in mortality in the other subgroups of patients were similar to those in the general population (Table 2 and all Supporting Tables).

## 4 DISCUSSION

This study analysed 135,831 patients with EC and found that approximately 41.5% died from non‐cancer causes. Over the years, the number of patients dying from EC has gradually decreased, and non‐cancer causes have become the leading cause of death. Heart disease, cerebrovascular disease and diabetes mellitus are the leading causes of non‐cancer deaths.

The incidence of EC has shown an increasing trend worldwide. Over the last 30 years, the overall incidence has increased by 132%. However, mortality rates are declining.^19^ Improvements in survival are probably related to the continuous improvement in treatment options, choice of surgical modalities^20^ and recommendations for adjuvant therapy.^21^ EC is treated primarily by surgery, with clinical risk factors determining whether adjuvant radiotherapy or chemotherapy should be administered.^4^ The survival rate for patients with EC who refused surgery was only 29.2%.^22^ Lymph node involvement is an important prognostic factor in patients with EC.^23^ Our study also found that most patients with lymph node metastasis died of EC. When treating EC, the National Comprehensive Cancer Network guidelines will improve the survival rate of patients.^24^ The therapeutic role of chemotherapy in EC remains controversial.^25^ In this study, 92.7% of patients with EC underwent surgery, 25.8% received radiotherapy and only 15.9% received chemotherapy. This could be related to the fact that only 6.7% of the patients had the advanced‐stage disease when EC was detected. Owing to the local stages found and perfected treatment modalities, patients with EC can achieve a survival rate of 81.3% within 5 years after diagnosis.^26^ Accordingly, deaths due to other diseases significantly impact on the survival of patients with EC. Preventing and tackling non‐EC‐induced deaths may prolong the lives of patients with EC.

Studies have found that in patients with EC, the risk of death from cardiovascular disease in the first year of diagnosis is extremely high.^27^ Cardiovascular diseases include heart disease, hypertension, cerebrovascular disease, atherosclerosis and aortic aneurysms/dissections. Obesity is an independent risk factor for EC. Similarly, obesity is also a high‐risk factor for cerebrovascular diseases.^28^ Bariatric surgery can reduce the incidence of postoperative complications in patients with EC, but it does not affect the survival rate of patients with EC.^29^ As a result, we attempted to obtain data from public databases and employed reasonable methods to describe the cause of death in EC.^30^, ^31^ Our study showed similar results: patients with EC had an increased risk of death from cardiovascular and cerebrovascular diseases in the first year or more than 10 years after diagnosis. These results indicate that monitoring cardiovascular health during the initial diagnosis of EC and the treatment phase is significant. During follow‐up, individuals under 65 years, Non‐Hispanic, non‐distant stage, non‐grade IV, received radiotherapy or underwent surgery, unmarried and do not have a specific endometriosis subtype should have their heart systems examined. Conversely those with a regional stage, Non‐Hispanic Asian or Pacific Islander, Hispanic, grade III, surgery, SWD status or non‐endometrioid subtype, should pay close attention to their cerebrovascular system.

A few studies believe that chemotherapy is an important factor in increasing the cause of death from heart disease owing to the cardiotoxicity of chemotherapy drugs.^32^ However when we analysed patients who received chemotherapy, the risk of sepsis increased, but the risk of death due to heart disease did not appear to increase significantly. This may be linked to the limitations of the chemotherapy data provided by the SEER database.

Studies have suggested that diabetes mellitus may increase the risk of death and cardiovascular‐specific death in patients with EC.^33^ A study proved that metformin and a diabetes risk‐reducing diet could prolong the survival of patients with EC.^34^, ^35^ Our study also found that diabetes was a high‐risk cause of non‐cancer death in patients with EC at different periods after diagnosis, especially in survivors over 49 years old, at stage N0, and who received radiotherapy and underwent surgery. In other words, we can screen patients with EC, regardless of whether they have diabetes mellitus, and treat them with early intervention. This may reduce the non‐cancer causes of death in patients with EC.

In this study, we found that within 10 years of EC diagnosis, the risk of death from sepsis was high, especially in patients receiving surgery, chemotherapy and radiation therapy. Patients with EC in the distant stage, Non‐Hispanic Black and Non‐Hispanic Asian or Pacific Islander, N1/N2 stage, received chemotherapy and sarcoma subtypes had a higher risk of sepsis death. Studies show differences in cancer survival among races, possibly related to early treatment and better care.^36^ Better hospital care could prevent one in every eight sepsis‐related deaths.^37^ Considering this, prevention can be achieved through guided care and education on the early signs of infection.

Cancer survivors had a 10% higher risk of developing subsequent primary cancers (SPCs) and a 33% higher risk of death from SPCs than the general population.^38^ The inclinations of SPCs in EC survivors tend to occur in the small intestine, kidney, oral cavity, pharynx, lymphoma, lung and breast.^39^ The study also found that 15.6% of patients with EC died from other malignancies during their survival. The research considered that when stratified by histological subtype, women diagnosed with serous or carcinosarcoma tumours had significantly higher SPC risks combined.^40^ We also found that patients with sarcoma‐subtype EC had a higher risk of death from other cancers. Our data would inform physicians of the development of SPCs in EC survivors.

### 4.1 Strengths and limitations

In the current study, we conducted a description of non‐cancer deaths based on enough patients registered in the SEER database and analysed variations in the risk of each cause of death. Based on the changes in SMR in EC patients with different characteristics at different times after cancer diagnosis, we can formulate nursing plans for patients with EC in the survival process to prevent or timely discover the high‐risk factors of non‐cancer death and achieve an improved quality of life and prolonged survival. To reduce the risk of bias through misspecification, we included all patients with EC who met the screening criteria and were registered in the SEER database. The SEER database constantly updates data, and there is no detailed information about other specific cancer deaths, which is also a limitation of this study. To the best of our knowledge, SEER‐Medicare offers data from various sources and may address this issue.^41^ However, we did not have access to this information. Unfortunately, the SEER database does not contain detailed information about current and emerging prognostic biomarkers in EC, such as molecular subtyping, obesity and socioeconomic status.^42^ Alternatively, some novel biomarkers have been proven helpful for the early diagnosis of EC,^43^, ^44^ which may improve survival^45^ and significantly impact the distribution of deaths from different causes. The current SEER database does not have access to this data. Furthermore, we cannot make definitive conclusions about the risk of causes of death in connection with treatment type since some of these patients may still be receiving different forms of adjuvant treatments that are not reported in SEER as the first course of treatment.^46^ Finally, the current work would be strengthened if the SEER programme could provide additional information on comorbidities before or after EC diagnosis

## 5 CONCLUSIONS

Deaths from non‐EC accounted for the majority of deaths in patients with EC. The leading causes of non‐cancer death include heart disease, cerebrovascular disease and diabetes. Compared with the US general population, patients with EC have a higher risk of death from sepsis and diabetes and a lower risk of death from COPD. With the development of preventive measures and regular follow‐up for the high‐risk causes of death identified by the institute, a better quality of life could be obtained for EC survivors

## AUTHOR CONTRIBUTIONS

**Qing Xue:** Conceptualization (lead); data curation (lead); formal analysis (lead); methodology (lead); visualization (lead); writing – original draft (lead); writing – review and editing (lead). **Wenqiang Che:** Writing – original draft (equal). **Lujiadai Xue:** Data curation (equal). **Xian Zhang:** Project administration (supporting); supervision (supporting). **Xiaoyu Wang:** Conceptualization (lead); project administration (lead); supervision (lead); writing – review and editing (lead). **Jun Lyu:** Conceptualization (lead); formal analysis (equal); project administration (equal); supervision (equal); writing – review and editing (lead).

## FUNDING INFORMATION

Xian Zhang was supported by ‘the Fundamental Research Funds for the Central Universities’ (project number: 21620310) and Medical Joint Fund of Jinan University (MF220215, MF220216).

## CONFLICT OF INTEREST STATEMENT

The authors declared no conflict of interest.

## Supporting information


Tables S1.

## Data Availability

Publicly available datasets were analysed in this study. These data can be found here: https://seer.cancer.gov/.
