# Supplementary material for: Causes of death in endometrial cancer survivors: A Surveillance, Epidemiology, and End Result–based analysis
Source: Cancer Med. 2023 Mar 16;12(9):10917–30. doi: 10.1002/cam4.5804 (PMC10225232; doi:10.1002/cam4.5804)
Supplement: Supplementary file 1 — Tables S1. [file CAM4-12-10917-s001.docx]

**Supporting Table 1.** Definition of each cause of death and corresponding codes in the ICD-10 of Diseases and Related Health.

| Non-Cancer Causes of Death | ICD-10 corresponding codes | Cause of death definition |
| --- | --- | --- |
| In situ, benign or unknown behavior neoplasms | D00-D09 | In situ neoplasms |
|  | D10-D36 | Benign neoplasms |
|  | D37-D48 | Neoplasms of uncertain or unknown behavior |
| Septicemia | A40-A41 | Sepsis |
| Other Infectious Diseases |  |  |
| Diabetes Mellitus | E10-E14 | Diabetes mellitus |
| Alzheimer’s | G30 | Alzheimer disease |
| Diseases of Heart | I00-I02 | Acute rheumatic fever |
|  | I05-I09 | Chronic rheumatic heart diseases |
|  | I11 | Hypertensive heart disease |
|  | I13 | Hypertensive heart and renal disease |
|  | I20-I25 | Ischemic heart diseases |
|  | I26-I28 | Pulmonary heart disease and diseases of pulmonary circulation |
|  | I30-I32 | Diseases of pericardium |
|  | I33 | Acute and subacute endocarditis |
|  | I34-I39 | Nonrheumatic valve disorders |
|  | I40-I41 | Myocarditis |
|  | I42-I43 | Cardiomyopathy |
|  | I44-I45 | Conduction disorders |
|  | I46 | Cardiac arrest |
|  | I47-I49 | Arrythmias |
|  | I50 | Heart failure |
|  | I51 | Complications and ill-defined descriptions of heart disease |
| Hypertension without Heart Disease | I10 | Essential (primary) hypertension |
|  | I12 | Hypertensive renal disease |
| Cerebrovascular Diseases | I60-I62 | Nontraumatic intracranial haemorrhage |
|  | I63 | Cerebral infarction |
|  | I64 | Stroke, not specified as haemorrhage or infarction |
|  | I65-I66 | Occlusion and stenosis of precerebral/cerebral arteries, not resulting in cerebral infarction |
|  | I67-I69 | Other cerebrovascular diseases or Sequelae of cerebrovascular disease |
| Atherosclerosis | I70 | Atherosclerosis |
| Aortic Aneurysm and Dissection | I71 | Aortic Aneurysm and Dissection |
| Other Diseases of Arteries, Arterioles, Capillaries | I72-I73 | Other aneurysm and dissection or other peripheral vascular diseases |
|  | I74 | Arterial embolism and thrombosis |
|  | I77 | Other disorders of arteries and arterioles |
|  | I78 | Diseases of capillaries |
| Pneumonia and Influenza | J09-J18 | Influenza and pneumonia |
| Chronic Obstructive Pulmonary Disease | J40-J42 | Bronchitis |
|  | J43 | Emphysema |
|  | J44 | Other chronic obstructive pulmonary disease |
|  | J45-J46 | Asthma or Status asthmaticus |
|  | J47 | Bronchiectasis |
| Chronic Liver Disease and Cirrhosis | K70 | Alcoholic liver disease |
|  | K73 | Chronic hepatitis |
|  | K74 | Fibrosis and cirrhosis of liver |
| Nephritis, Nephrotic Syndrome and Nephrosis | N00-N07 | Glomerular diseases |
|  | N17-N19 | Renal failure |
|  | N25 | Disorders resulting from impaired renal tubular function |
|  | N26 | Unspecified contracted kidney |
|  | N27 | Small kidney of unknown cause |
| Symptoms, Signs and Ill-Defined Conditions | R00-R99 | Symptoms, signs, abnormal results of clinical or other investigative procedures, and ill-defined conditions regarding which no diagnosis classifiable elsewhere is recorded. |
| Accidents and Adverse Effects | V01-V99 | Transport accidents |
|  | W00-X59 | Other external causes of accidental injury |
|  | Y85-Y86 | Sequelae of transport accidents or other accidents |
| Suicide and Self-Inflicted Injury | X60-X84 | Intentional self-harm |
|  | Y87 | Sequelae of intentional self-harm, assault and events of undetermined intent |
| Other Cause of Death |  |  |

**Supporting Table 2.** Standardized-mortality ratios (SMRs) for each cause of death following endometrial cancer diagnosis in females aged younger than 49 years.

| **Timing of deaths after diagnosis** | | | | | | | | | | |
| --- | --- | --- | --- | --- | --- | --- | --- | --- | --- | --- |
|  | **＜1year** | | **1-5years** | | **5-10years** | | **＞10years** | | **Total** | |
| **Causes of death** | **No.observed** | **SMR(95% CI)** | **No.observed** | **SMR(95% CI)** | **No.observed** | **SMR(95% CI)** | **No.observed** | **SMR(95% CI)** | **No.observed** | **SMR(95% CI)** |
| **All Causes of Death** | 470 | 18.51(16.87-20.26)* | 858 | 6.81(6.36-7.28)* | 6 | 7.98(2.93-17.38)* | 402 | 2.02(1.83-2.23)* | 11 | 4.63(2.31-8.29)* |
| **Endometrial Cancer deaths** | 364 | 581.38(523.18-644.28)* | 532 | 173.43(159-188.81)* | 7 | 2.59(1.04-5.33)* | 40 | 8.50(6.08-11.58)* | 32 | 3.80(2.6-5.37)* |
| **Other cancer causes of deaths** | 53 | 7.17(5.37-9.37)* | 152 | 3.94(3.34-4.62)* | 4 | 1.28(0.35-3.28) | 88 | 1.31(1.05-1.62)* | 16 | 1.67(0.96-2.72) |
| **No-cancer causes of deaths** | 53 | 3.05(2.29-3.99)* | 174 | 2.06(1.77-2.39)* | 22 | 4.00(2.51-6.06)* | 274 | 2.16(1.91-2.43)* | 57 | 3.31(2.51-4.29)* |
| **In situ, benign or unknown behavior neoplasm** | 1 | 8.1(0.21-45.14) | 2 | 3.44(0.42-12.41) | 0 | 0(0-14.61) | 2 | 2.18(0.26-7.87) | 0 | 0(0-3) |
| **Septicemia** | 6 | 16.65(6.11-36.23)* | 8 | 4.25(1.83-8.37)* | 41 | 1.69(1.21-2.3)* | 11 | 3.17(1.58-5.68)* | 146 | 1.92(1.62-2.26)* |
| **Other Infectious and Parasitic Diseases including HIV** | 2 | 2.86(0.35-10.34) | 3 | 1.02(0.21-2.99) | 3 | 2.59(0.53-7.57) | 7 | 2.50(1-5.14)* | 14 | 3.76(2.05-6.31)* |
| **Diabetes Mellitus** | 3 | 4.27(0.88-12.47) | 12 | 3.19(1.65-5.57)* | 7 | 1.21(0.49-2.5) | 20 | 2.75(1.68-4.25)* | 27 | 1.48(0.98-2.15) |
| **Alzheimers (ICD-9 and 10 only)** | 0 | 0(0-333.71) | 0 | 0(0-42.59) | 0 | 0(0-33.52) | 0 | 0(0-4.19) | 1 | 2.8(0.071-5.61) |
| **Diseases of Heart** | 11 | 3.35(1.67-5.99)* | 34 | 1.99(1.38-2.78)* | 0 | 0(0-9.73) | 60 | 1.91(1.46-2.46)* | 2 | 1.62(0.2-5.87) |
| **Hypertension without Heart Disease** | 1 | 7.98(0.24-4.47) | 4 | 5.53(1.51-14.16)* | 1 | 2.49(0.06-13.87) | 6 | 3.49(1.28-7.6)* | 4 | 3.03(0.83-7.77) |
| **Cerebrovascular Diseases** | 0 | 0(0-4.07) | 7 | 1.57(0.63-3.24) | 2 | 0.84(0.1-3.03) | 13 | 1.83(0.97-3.13) | 18 | 2.39(1.42-3.78)* |
| **Atherosclerosis** | 0 | 0(0-289.48) | 0 | 0(0-52.91) | 5 | 0.74(0.24-1.73) | 1 | 6.08(0.15-33.9) | 21 | 0.94(0.58-1.44) |
| **Aortic Aneurysm and Dissection** | 0 | 0(0-63.07) | 1 | 3.47(0.09-19.33) | 0 | 0(0-16.77) | 1 | 1.98(0.05-11.01) | 0 | 0(0-5.4) |
| **Other Diseases of Arteries, Arterioles, Capillaries** | 0 | 0(0-67.09) | 1 | 3.57(0.09-19.92) | 8 | 1.2(0.52-2.36) | 2 | 3.44(0.42-12.41) | 24 | 1.24(0.79-1.84) |
| **Pneumonia and Influenza** | 1 | 2.94(0.07-16.37) | 4 | 2.33(0.63-5.96) | 11 | 5.30(2.65-9.48)* | 11 | 3.58(1.79-6.4)* | 35 | 5.28(3.68-7.34)* |
| **Chronic Obstructive Pulmonary Disease and Allied Cond** | 2 | 3.59(0.43-12.95) | 4 | 1.14(0.31-2.92) | 1 | 4.5(50.12-25.36) | 10 | 0.87(0.42-1.59) | 4 | 5.99(1.63-15.33)* |
| **Stomach and Duodenal Ulcers** | 0 | 0(0-122.2) | 0 | 0(0-23.98) | 0 | 0(0-4.46) | 0 | 0(0-13.19) | 1 | 0.4(0.01-2.24) |
| **Chronic Liver Disease and Cirrhosis** | 1 | 1.02(0.03-5.69) | 6 | 1.19(0.44-2.59) | 0 | 0(0-721.4) | 9 | 1.34(0.61-2.55) | 0 | 0(0-237.42) |
| **Nephritis, Nephrotic Syndrome and Nephrosis** | 2 | 7.53(0.91-27.22) | 8 | 5.72(2.47-11.27)* | 6 | 3.17(1.16-6.9)* | 14 | 4.84(2.65-8.12)* | 13 | 2.21(1.18-3.78)* |
| **Complications of Pregnancy, Childbirth, Puerperium** | 1 | 13.59(0.34-75.74) | 2 | 6.74(0.82-24.34) | 13 | 0.87(0.46-1.49) | 0 | 0(0-47.23) | 46 | 1.04(0.76-1.39) |
| **Congenital Anomalies** | 0 | 0(0-23.92) | 1 | 1.45(0.04-8.05) | 6 | 1.2(0.44-2.61) | 0 | 0(0-4.54) | 18 | 1.23(0.73-1.95) |
| **Certain Conditions Originating in Perinatal Period** | 0 | 0(0-3012.01) | 0 | 0(0-712.52) | 0 | 0(0-3.32) | 0 | 0(0-917.24) | 0 | 0(0-1.04) |
| **Symptoms, Signs and Ill-Defined Conditions** | 1 | 2.27(0.06-12.63) | 5 | 2.79(0.91-6.51) | 43 | 1.93(1.39-2.59)* | 1 | 0.57(0.01-3.18) | 150 | 2.14(1.81-2.51)* |
| **Accidents and Adverse Effects** | 4 | 1.24(0.34-3.18) | 15 | 1.04(0.58-1.72) | 433 | 2.58(2.35-2.84)* | 14 | 1.21(0.66-2.03) | 2163 | 4.18(4-4.36)* |
| **Suicide and Self-Inflicted Injury** | 1 | 0.91(0.02-5.06) | 5 | 1.03(0.33-2.41) | 142 | 35.25(29.69-41.55)* | 6 | 1.64(0.6-3.57) | 1078 | 86.76(81.66-92.1)* |
| **Homicide and Legal Intervention** | 0 | 0(0-9.84) | 0 | 0(0-2.73) | 95 | 1.73(1.4-2.12)* | 0 | 0(0-5.17) | 388 | 2.31(2.09-2.55)* |
| **Other Cause of Death** | 8 | 2.3(0.99-4.53) | 30 | 1.76(1.19-2.51)* | 196 | 1.80(1.56-2.07)* | 69 | 2.54(1.98-3.22)* | 697 | 2.06(1.91-2.22)* |

Abbreviation: SMR, standardized mortality ratio. CI: confidence interval

Values indicate the number of patients with cancer who died from each cause of death.

***** P value less than .05

**Supporting Table 3.** Standardized-mortality ratios (SMRs) for each cause of death following Endometrial Cancer diagnosis in females aged 50-64 years.

| **Timing of deaths after diagnosis** | | | | | | | | | | |
| --- | --- | --- | --- | --- | --- | --- | --- | --- | --- | --- |
|  | **＜1year** | | **1-5years** | | **5-10years** | | **＞10years** | | **Total** | |
| **Causes of death** | **No.observed** | **SMR(95% CI)** | **No.observed** | **SMR(95% CI)** | **No.observed** | **SMR(95% CI)** | **No.observed** | **SMR(95% CI)** | **No.observed** | **SMR(95% CI)** |
| **All Causes of Death** | 1,984 | 7.387.06-7.71)* | 4,872 | 3.83(3.72-3.94)* | 2,442 | 1.59(1.52-1.65)* | 1,928 | 1.19(1.14-1.24)* | 11,226 | 2.39(2.34-2.43)* |
| **Endometrial Cancer deaths** | 1,480 | 251.02238.39-264.14)* | 3,160 | 115.12(111.15-119.21)* | 728 | 23.62(21.94-25.4)* | 161 | 6.33(5.39-7.38)* | 5,529 | 61.70(60.09-63.35)* |
| **Other cancer causes of deaths** | 203 | 2.00(1.74-2.3)* | 757 | 1.62(1.5-1.73)* | 581 | 1.08(1-1.18) | 467 | 0.97(0.89-1.07) | 2,008 | 1.27(1.21-1.32)* |
| **Non-cancer causes of deaths** | 301 | 1.86(1.66-2.08)* | 955 | 1.23(1.15-1.31)* | 1,133 | 1.16(1.1-1.23)* | 1,300 | 1.16(1.1-1.23)* | 3,689 | 1.22(1.18-1.26)* |
| **In situ, benign or unknown behavior neoplasm** | 4 | 2.89(0.79-7.39) | 17 | 2.57(1.5-4.11)* | 8 | 0.94(0.4-1.85) | 14 | 1.44(0.79-2.41) | 43 | 1.64(1.18-2.2)* |
| **Septicemia** | 15 | 3.28(1.83-5.4)* | 33 | 1.47(1-2.06)* | 42 | 1.50(1.08-2.02)* | 38 | 1.31(0.92-1.79) | 128 | 1.52(1.27-1.81)* |
| **Other Infectious and Parasitic Diseases including HIV** | 7 | 1.81(0.73-3.73) | 24 | 1.48(1-2.2) | 27 | 1.64(1.08-2.38)* | 11 | 0.8(0.4-1.43) | 69 | 1.37(1.07-1.73)* |
| **Diabetes Mellitus** | 19 | 1.81(1.09-2.83)* | 84 | 1.70(1.36-2.11)* | 96 | 1.65(1.34-2.02)* | 108 | 1.92(1.57-2.32)* | 307 | 1.76(1.57-1.97)* |
| **Alzheimers (ICD-9 and 10 only)** | 2 | 2.08(0.25-7.51) | 4 | 0.57(0.16-1.46) | 18 | 1.05(0.62-1.66) | 36 | 0.76(0.53-1.06) | 60 | 0.83(0.63-1.07) |
| **Diseases of Heart** | 81 | 1.74(1.39-2.17)* | 265 | 1.21(1.07-1.36)* | 340 | 1.26(1.13-1.4)* | 348 | 1.15(1.03-1.28)* | 1,034 | 1.23(1.16-1.31)* |
| **Hypertension without Heart Disease** | 3 | 1.48(0.3-4.31) | 18 | 1.71(1.01-2.7)* | 22 | 1.53(0.96-2.31) | 25 | 1.4(0.9-2.06) | 68 | 1.52(1.18-1.92)* |
| **Cerebrovascular Diseases** | 21 | 2.00(1.24-3.06)* | 50 | 1(0.74-1.32) | 62 | 0.96(0.74-1.23) | 89 | 1.09(0.87-1.34) | 222 | 1.07(0.94-1.22) |
| **Atherosclerosis** | 1 | 3(0.08-16.69) | 0 | 0(0-2.27) | 1 | 0.49(0.01-2.71) | 4 | 1.63(0.44-4.17) | 6 | 0.93(0.34-2.02) |
| **Aortic Aneurysm and Dissection** | 0 | 0(0-4.59) | 0 | 0.00(0-0.93)* | 3 | 0.59(0.12-1.71) | 4 | 0.68(0.19-1.75) | 7 | 0.44(0.18-0.91)* |
| **Other Diseases of Arteries, Arterioles, Capillaries** | 2 | 2.44(0.3-8.81) | 2 | 0.49(0-1.78) | 2 | 0.38(0.05-1.36) | 3 | 0.49(0.1-1.45) | 9 | 0.55(0.25-1.05) |
| **Pneumonia and Influenza** | 9 | 2.49(1.14-4.72)* | 21 | 1.15(0.71-1.75) | 36 | 1.48(1.03-2.04)* | 27 | 0.93(0.61-1.35) | 93 | 1.24(1-1.51) |
| **Chronic Obstructive Pulmonary Disease and Allied Cond** | 12 | 0.79(0.41-1.38) | 34 | 0.41(0.28-0.57)* | 56 | 0.46(0.35-0.6)* | 93 | 0.65(0.53-0.8)* | 195 | 0.54(0.46-0.62)* |
| **Stomach and Duodenal Ulcers** | 0 | 0(0-10.3) | 2 | 1.2(0.15-4.34) | 3 | 1.54(0.32-4.5) | 5 | 2.42(0.79-5.64) | 10 | 1.66(0.79-3.04) |
| **Chronic Liver Disease and Cirrhosis** | 7 | 1.09(0.44-2.24) | 18 | 0.64(0.38-1) | 31 | 1.09(0.74-1.54) | 20 | 0.94(0.57-1.45) | 76 | 0.9(0.71-1.13) |
| **Nephritis, Nephrotic Syndrome and Nephrosis** | 11 | 2.60(1.3-4.65)* | 35 | 1.64(1.15-2.29)* | 35 | 1.24(0.86-1.72) | 49 | 1.53(1.13-2.02)* | 130 | 1.52(1.27-1.8)* |
| **Complications of Pregnancy, Childbirth, Puerperium** | 0 | 0(0-103.92) | 0 | 0(0-48.38) | 0 | 0(0-431.88) | 0 | 0(0-2,681.04) | 0 | 0(0-30.32) |
| **Congenital Anomalies** | 1 | 1.15(0.03-6.39) | 3 | 0.84(0.17-2.46) | 3 | 0.97(0.2-2.84) | 3 | 1.56(0.32-4.56) | 10 | 1.06(0.51-1.95) |
| **Certain Conditions Originating in Perinatal Period** | 0 | 0(0-1619.96) | 0 | 0(0-374.1) | 0 | 0(0-394.26) | 0 | 0(0-638.9) | 0 | 0(0-135.28) |
| **Symptoms, Signs and Ill-Defined Conditions** | 8 | 3.53(1.52-6.95)* | 13 | 1.31(0.7-2.23) | 20 | 1.70(1.04-2.63)* | 11 | 0.87(0.43-1.55) | 52 | 1.42(1.06-1.86)* |
| **Accidents and Adverse Effects** | 6 | 0.58(0.21-1.26) | 38 | 0.87(0.62-1.2) | 37 | 0.88(0.62-1.21) | 30 | 0.78(0.53-1.12) | 111 | 0.83(0.68-1)* |
| **Suicide and Self-Inflicted Injury** | 2 | 0.59(0.07-2.13) | 4 | 0.30(0.08-0.78)* | 9 | 0.84(0.39-1.6) | 3 | 0.51(0.1-1.48) | 18 | 0.54(0.32-0.86)* |
| **Homicide and Legal Intervention** | 0 | 0(0-5.2) | 2 | 0.76(0.09-2.75) | 1 | 0.47(0.01-2.6) | 1 | 0.74(0.02-4.14) | 4 | 0.59(0.16-1.5) |
| **Other Cause of Death** | 73 | 2.28(1.79-2.87)* | 205 | 1.30(1.12-1.49)* | 236 | 1.13(0.99-1.29) | 305 | 1.19(1.06-1.33)* | 819 | 1.25(1.17-1.34)* |

Abbreviation: SMR, standardized mortality ratio. CI: confidence interval

Values indicate the number of patients with cancer who died from each cause of death.

* P value less than .05

**Supporting Table 4.** Standardized-mortality ratios (SMRs) for each cause of death following Endometrial Cancer diagnosis in females older than 64 years.

| **Timing of deaths after diagnosis** | | | | | | | | | | |
| --- | --- | --- | --- | --- | --- | --- | --- | --- | --- | --- |
|  | **＜1year** | | **1-5years** | | **5-10years** | | **＞10years** | | **Total** | |
| **Causes of death** | **No.observed** | **SMR(95% CI)** | **No.observed** | **SMR(95% CI)** | **No.observed** | **SMR(95% CI)** | **No.observed** | **SMR(95% CI)** | **No.observed** | **SMR(95% CI)** |
| **All Causes of Death** | 3,955 | 3.26(3.16-3.36)* | 9,026 | 1.86(1.82-1.9)* | 5,387 | 1.10(1.07-1.13)* | 4,033 | 1.09(1.06-1.13)* | 22,401 | 1.53(1.51-1.55)* |
| **Endometrial Cancer deaths** | 2,597 | 256.63(246.85-266.69)* | 4,587 | 122.61(119.09-126.21)* | 835 | 27.51(25.68-29.44)* | 124 | 7.39(6.15-8.81)* | 8,143 | 86.02(84.17-87.91)* |
| **Other cancer causes of deaths** | 413 | 1.58(1.43-1.74)* | 1,398 | 1.43(1.36-1.51)* | 924 | 1.11(1.04-1.19)* | 496 | 1.01(0.92-1.1) | 3,231 | 1.26(1.22-1.31)* |
| **Non-cancer causes of deaths** | 945 | 1(0.94-1.07) | 3,041 | 0.79(0.76-0.82)* | 3,628 | 0.90(0.87-0.93)* | 3,413 | 1.07(1.04-1.11)* | 11,027 | 0.92(0.9-0.94)* |
| **In situ, benign or unknown behavior neoplasm** | 12 | 1.66(0.86-2.9) | 35 | 1.2(0.84-1.67) | 30 | 1.03(0.69-1.47) | 25 | 1.18(0.76-1.74) | 102 | 1.18(0.96-1.43) |
| **Septicemia** | 35 | 1.83(1.28-3)* | 77 | 1.03(1-1.28) | 81 | 1.13(0.9-1.41) | 52 | 1.06(0.79-1.39) | 245 | 1.14(1-1.29)* |
| **Other Infectious and Parasitic Diseases including HIV** | 10 | 1.22(0.58-2) | 28 | 0.83(1-1.2) | 37 | 1.13(0.79-1.55) | 31 | 1.47(1-2.09) | 106 | 1.11(0.91-1.34) |
| **Diabetes Mellitus** | 56 | 1.50(1.13-2)* | 162 | 1.17(1-1.36) | 144 | 1.18(0.99-1.39) | 105 | 1.35(1.1-1.63)* | 467 | 1.24(1.13-1.36)* |
| **Alzheimers (ICD-9 and 10 only)** | 24 | 0.44(0.28-0.65)* | 123 | 0.47(0.39-0.56)* | 274 | 0.82(0.72-0.92)* | 383 | 1.18(1.06-1.3)* | 804 | 0.82(0.77-0.88)* |
| **Diseases of Heart** | 360 | 1.13(1.01-1.25)* | 1,004 | 0.81(0.76-0.86)* | 1,175 | 0.95(0.89-1) | 1,019 | 1.06(1-1.13) | 3,558 | 0.94(0.91-0.98)* |
| **Hypertension without Heart Disease** | 13 | 0.86(0.46-1.48) | 62 | 0.96(0.73-1.23) | 84 | 1.16(0.92-1.43) | 94 | 1.53(1.24-1.87)* | 253 | 1.18(1.04-1.34)* |
| **Cerebrovascular Diseases** | 92 | 1.05(0.84-1.28) | 284 | 0.82(0.73-0.92)* | 313 | 0.9(0.8-1) | 307 | 1.1(0.98-1.23) | 996 | 0.94(0.88-1)* |
| **Atherosclerosis** | 5 | 0.84(0.27-1.97) | 27 | 1.28(0.84-1.86) | 17 | 0.93(0.54-1.49) | 18 | 1.44(0.85-2.27) | 67 | 1.16(0.9-1.47) |
| **Aortic Aneurysm and Dissection** | 2 | 0.36(0.04-1.28) | 15 | 0.72(0.41-1.19) | 7 | 0.39(0.16-0.8)* | 7 | 0.6(0.24-1.24) | 31 | 0.55(0.38-0.79)* |
| **Other Diseases of Arteries, Arterioles, Capillaries** | 8 | 1.48(0.64-2.93) | 20 | 0.95(0.58-1.47) | 15 | 0.73(0.41-1.21) | 12 | 0.79(0.41-1.38) | 55 | 0.89(0.67-1.15) |
| **Pneumonia and Influenza** | 18 | 0.58(0.34-0.92)* | 95 | 0.77(0.63-0.95)* | 114 | 0.94(0.78-1.13) | 94 | 1.03(0.83-1.26) | 321 | 0.88(0.78-0.98)* |
| **Chronic Obstructive Pulmonary Disease and Allied Cond** | 41 | 0.50(0.36-0.68)* | 140 | 0.42(0.35-0.5)* | 194 | 0.60(0.52-0.69)* | 150 | 0.68(0.58-0.8)* | 525 | 0.55(0.5-0.6)* |
| **Stomach and Duodenal Ulcers** | 4 | 2.25(0.61-6) | 11 | 1.68(1-3) | 8 | 1.33(0.58-2.63) | 3 | 0.7(0.14-2.04) | 26 | 1.4(0.91-2.05) |
| **Chronic Liver Disease and Cirrhosis** | 10 | 1.32(0.64-2.44) | 33 | 1.19(0.82-1.67) | 19 | 0.88(0.53-1.38) | 10 | 0.89(0.43-1.64) | 72 | 1.06(0.83-1.33) |
| **Nephritis, Nephrotic Syndrome and Nephrosis** | 29 | 1.21(0.81-1.74) | 107 | 1.11(0.91-1.34) | 107 | 1.12(0.91-1.35) | 83 | 1.24(0.98-1.53) | 326 | 1.15(1.03-1.28)* |
| **Complications of Pregnancy, Childbirth, Puerperium** | 0 | 0(0-4,227.15) | 0 | 0(0-1,849.24) | 0 | 0(0-5,234.33) | 0 | 0(0-21,836.43) | 0 | 0(0-986.03) |
| **Congenital Anomalies** | 1 | 1.13(0.03-6.27) | 0 | 0(0-1.16) | 3 | 1.16(0.24-3.38) | 3 | 1.81(0.37-5.29) | 7 | 0.84(0.34-1.73) |
| **Certain Conditions Originating in Perinatal Period** | 0 | 0(0-2,031.29) | 0 | 0(0-604.49) | 0 | 0(0-687.25) | 0 | 0(0-1,033.86) | 0 | 0(0-218.87) |
| **Symptoms, Signs and Ill-Defined Conditions** | 11 | 0.73(0.36-1.3) | 56 | 0.86(0.65-1.12) | 59 | 0.78(0.6-1.01) | 42 | 0.76(0.55-1.03) | 168 | 0.80(0.68-0.93)* |
| **Accidents and Adverse Effects** | 19 | 0.79(0.48-1.23) | 60 | 0.59(0.45-0.75)* | 75 | 0.67(0.53-0.84)* | 75 | 0.8(0.63-1.01) | 229 | 0.69(0.6-0.79)* |
| **Suicide and Self-Inflicted Injury** | 0 | 0(0-2.32) | 1 | 0.18(0-0.98)* | 2 | 0.5(0.06-1.8) | 4 | 2.08(0.57-5.34) | 7 | 0.53(0.21-1.09) |
| **Homicide and Legal Intervention** | 0 | 0(0-6.71) | 2 | 1.04(0.13-3.76) | 2 | 1.36(0.16-4.91) | 2 | 2.59(0.31-9.37) | 6 | 1.27(0.47-2.77) |
| **Other Cause of Death** | 166 | 0.89(0.76-1.03) | 591 | 0.72(0.66-0.78)* | 763 | 0.81(0.75-0.87)* | 798 | 1(0.93-1.08) | 2,318 | 0.84(0.81-0.88)* |

Abbreviation: SMR, standardized mortality ratio. CI: confidence interval

Values indicate the number of patients with cancer who died from each cause of death.

* P value less than .05

**Supporting Table 5.** Standardized-mortality ratios (SMRs) for each cause of death following Endometrial Cancer diagnosis in Non-Hispanic White.

| **Timing of deaths after diagnosis** | | | | | | | | | | |
| --- | --- | --- | --- | --- | --- | --- | --- | --- | --- | --- |
|  | **＜1year** | | **1-5years** | | **5-10years** | | **＞10years** | | **Total** | |
| **Causes of death** | **No.observed** | **SMR(95% CI)** | **No.observed** | **SMR(95% CI)** | **No.observed** | **SMR(95% CI)** | **No.observed** | **SMR(95% CI)** | **No.observed** | **SMR(95% CI)** |
| **All Causes of Death** | 6231 | 3.99(3.89-4.09)* | 13612 | 2.14(2.11-2.18)* | 7807 | 1.21(1.18-1.24)* | 6,021 | 1.14(1.11-1.17)* | 33,671 | 1.71(1.69-1.73)* |
| **Endometrial Cancer deaths** | 4150 | 273.63(265.36-282.08)* | 7305 | 117.30(114.63-120.02)* | 1501 | 25.82(24.53-27.16)* | 307 | 7.40(6.6-8.38)* | 13,263 | 74.90(73.64-76.19)* |
| **Other cancer causes of deaths** | 672 | 1.81(1.67-1.95)* | 2118 | 1.43(1.37-1.5)* | 1473 | 1.08(1.02-1.13)* | 976 | 0.99(0.93-1.06) | 5,239 | 1.25(1.21-1.28) |
| **Non-cancer causes of deaths** | 1409 | 1.20(1.14-1.26)* | 4189 | 0.87(0.84-0.9)* | 4833 | 0.96(0.93-0.99)* | 4,738 | 1.11(1.08-1.94)* | 15,169 | 0.99(0.98-1.01) |
| **In situ, benign or unknown behavior neoplasm** | 19 | 2.06(1.24-3.22)* | 62 | 1.66(1.27-2.12)* | 42 | 1.1(0.8-1.49) | 44 | 1.43(1.04-1.02)* | 167 | 1.45(1.24-1.68)* |
| **Septicemia** | 57 | 2.43(1.84-3.15)* | 124 | 1.29(1.07-1.53)* | 124 | 1.29(1.07-1.53)* | 87 | 1.14(0.92-1.41) | 392 | 1.34(1.21-1.48)* |
| **Other Infectious and Parasitic Diseases including HIV** | 18 | 1.49(0.89-2.36) | 54 | 1.08(0.81-1.4) | 58 | 1.18(0.9-1.52) | 47 | 1.34(0.98-1.78) | 177 | 1.21(1.04-1.4)* |
| **Diabetes Mellitus** | 65 | 1.45(1.12-1.85)* | 251 | 1.42(1.25-1.61)* | 235 | 1.42(1.24-1.61)* | 174 | 1.40(1.2-1.22)* | 725 | 1.42(1.32-1.52)* |
| **Alzheimers (ICD-9 and 10 only)** | 34 | 0.53(0.37-0.74)* | 144 | 0.49(0.41-0.58)* | 307 | 0.85(0.75-0.95)* | 410 | 1.11(1-1.72)* | 895 | 0.82(0.77-0.87)* |
| **Diseases of Heart** | 495 | 1.28(1.17-1.4)* | 1324 | 0.88(0.83-0.92)* | 1531 | 1.01(0.96-1.06) | 1,392 | 1.12(1.06-1..8)* | 4,742 | 1.02(0.99-1.05) |
| **Hypertension without Heart Disease** | 24 | 1.44(0.92-2.14) | 81 | 1.11(0.88-1.38) | 90 | 1.1(0.88-1.35) | 112 | 1.51(1.25-1..2)* | 307 | 1.25(1.11-1.4)* |
| **Cerebrovascular Diseases** | 131 | 1.28(1.07-1.52)* | 336 | 0.84(0.75-0.93)* | 363 | 0.89(0.8-0.99)* | 373 | 1.07(0.96-1.19) | 1,203 | 0.95(0.9-1.01) |
| **Atherosclerosis** | 8 | 1.15(0.49-2.26) | 27 | 1.11(0.73-1.62) | 18 | 0.87(0.51-1.37) | 22 | 1.47(0.92-2.23) | 75 | 1.12(0.88-1.4) |
| **Aortic Aneurysm and Dissection** | 2 | 0.3(0.04-1.08) | 14 | 0.55(0.3-0.93)* | 10 | 0.43(0.21-0.8)* | 12 | 0.7(0.36-1.22) | 38 | 0.53(0.37-0.72)* |
| **Other Diseases of Arteries, Arterioles, Capillaries** | 9 | 1.4(0.64-2.66) | 25 | 0.98(0.63-1.45) | 20 | 0.79(0.48-1.21) | 14 | 0.67(0.37-1.13) | 68 | 0.87(0.68-1.1) |
| **Pneumonia and Influenza** | 39 | 1.04(0.74-1.42) | 121 | 0.82(0.68-0.98)* | 158 | 1.09(0.93-1.27) | 116 | 0.99(0.82-1.19) | 434 | 0.97(0.88-1.07) |
| **Chronic Obstructive Pulmonary Disease and Allied Cond** | 72 | 0.67(0.52-0.84)* | 196 | 0.43(0.37-0.5)* | 283 | 0.61(0.54-0.69)* | 256 | 0.69(0.6-0.57)* | 807 | 0.58(0.54-0.62)* |
| **Stomach and Duodenal Ulcers** | 5 | 2.2(0.72-5.15) | 14 | 1.63(0.89-2.74) | 10 | 1.25(0.6-2.29) | 10 | 1.57(0.75-2.89) | 39 | 1.55(1.1-2.11)* |
| **Chronic Liver Disease and Cirrhosis** | 21 | 1.41(0.87-2.15) | 60 | 0.99(0.75-1.27) | 46 | 0.85(0.62-1.13) | 37 | 1.01(0.71-1.39) | 164 | 0.98(0.84-1.15) |
| **Nephritis, Nephrotic Syndrome and Nephrosis** | 34 | 1.27(0.88-1.77) | 120 | 1.07(0.89-1.28) | 138 | 1.19(1-1.41)* | 136 | 1.47(1.24-1.(4)* | 428 | 1.23(1.12-1.36)* |
| **Complications of Pregnancy, Childbirth, Puerperium** | 1 | 13.1(0.33-72.97) | 0 | 0(0-14.45) | 1 | 6.7(0.17-37.33) | 0 | 0(0-73.6) | 2 | 3.77(0.46-13.61) |
| **Congenital Anomalies** | 3 | 1.55(0.32-4.52) | 4 | 0.53(0.15-1.37) | 7 | 1.11(0.45-2.28) | 6 | 1.44(0.53-3.13) | 20 | 1(0.61-1.55) |
| **Certain Conditions Originating in Perinatal Period** | 0 | 0(0-670.41) | 0 | 0(0-171.42) | 0 | 0(0-191.85) | 0 | 0(0-296.08) | 0 | 0(0-62.83) |
| **Symptoms, Signs and Ill-Defined Conditions** | 29 | 1.50(1-2.15)* | 78 | 0.97(0.77-1.21) | 85 | 0.95(0.76-1.17) | 60 | 0.87(0.67-1.12) | 252 | 0.98(0.86-1.1) |
| **Accidents and Adverse Effects** | 35 | 0.89(0.62-1.24) | 124 | 0.75(0.63-0.9)* | 124 | 0.74(0.62-0.88)* | 123 | 0.88(0.73-1.05) | 406 | 0.80(0.72-0.88)* |
| **Suicide and Self-Inflicted Injury** | 8 | 1.3(0.56-2.57) | 15 | 0.63(0.35-1.04) | 20 | 1.06(0.65-1.64) | 12 | 1.12(0.58-1.95) | 55 | 0.93(0.7-1.2) |
| **Homicide and Legal Intervention** | 0 | 0(0-2.71) | 3 | 0.6(0.12-1.75) | 2 | 0.5(0.06-1.82) | 2 | 0.84(0.1-3.03) | 7 | 0.55(0.22-1.13) |
| **Other Cause of Death** | 256 | 1.07(0.95-1.21) | 871 | 0.84(0.78-0.9)* | 1039 | 0.89(0.83-0.94)* | 1124 | 1.07(1.01-1.83)* | 3290 | 0.94(0.91-0.97)* |

Abbreviation: SMR, standardized mortality ratio. CI: confidence interval

Values indicate the number of patients with cancer who died from each cause of death.

* P value less than .05

**Supporting Table 6.** Standardized-mortality ratios (SMRs) for each cause of death following Endometrial Cancer diagnosis in Non-Hispanic Black.

| **Timing of deaths after diagnosis** | | | | | | | | | | |
| --- | --- | --- | --- | --- | --- | --- | --- | --- | --- | --- |
|  | **＜1year** | | **1-5years** | | **5-10years** | | **＞10years** | | **Total** | |
| **Causes of death** | **No.observed** | **SMR(95% CI)** | **No.observed** | **SMR(95% CI)** | **No.observed** | **SMR(95% CI)** | **No.observed** | **SMR(95% CI)** | **No.observed** | **SMR(95% CI)** |
| **All Causes of Death** | 1730 | 9.39(8.96-9.85)* | 2719 | 4.54(4.37-4.72)* | 837 | 1.71(1.59-1.83)* | 420 | 1.30(1.18-1.43)* | 5706 | 3.57(3.48-3.67)* |
| **Endometrial Cancer deaths** | 1164 | 343.95(324.47-364.29)* | 1708 | 146.37(139.51-153.48)* | 254 | 28.27(24.9-31.97)* | 21 | 4.15(2.57-6.43) | 3,147 | 108.14(104.39-111.98)* |
| **Other cancer causes of deaths** | 206 | 4.81(4.17-5.51)* | 435 | 3.13(2.84-3.44)* | 169 | 1.60(1.37-1.86)* | 66 | 1.07(0.83-1.37) | 876 | 2.51(2.35-2.68)* |
| **Non-cancer causes of deaths** | 360 | 2.61(2.35-2.89)* | 576 | 1.29(1.18-1.4)* | 414 | 1.1(1-1.21) | 333 | 1.30(1.16-1.41)* | 1,683 | 1.38(1.32-1.45)* |
| **In situ, benign or unknown behavior neoplasm** | 8 | 9.34(4.03-18.4)* | 4 | 1.43(0.39-3.66) | 5 | 2.13(0.69-4.98) | 1 | 0.64(0.02-0.69) | 18 | 2.38(1.41-3.76)* |
| **Septicemia** | 25 | 5.39(3.49-7.96)* | 21 | 1.4(0.87-2.14) | 16 | 1.34(0.77-2.18) | 11 | 1.51(0.75-0.77) | 73 | 1.88(1.47-2.36)* |
| **Other Infectious and Parasitic Diseases including HIV** | 3 | 1.31(0.27-3.83) | 6 | 0.83(0.3-1.81) | 6 | 1.2(0.44-2.61) | 2 | 0.76(0.09-0.44) | 17 | 0.99(0.58-1.59) |
| **Diabetes Mellitus** | 29 | 2.85(1.91-4.09)* | 48 | 1.49(1.1-1.97)* | 32 | 1.29(0.88-1.81) | 27 | 1.77(1.17-1.52)* | 136 | 1.65(1.38-1.95)* |
| **Alzheimers (ICD-9 and 10 only)** | 6 | 1.41(0.52-3.08) | 15 | 0.96(0.54-1.59) | 15 | 0.85(0.47-1.4) | 18 | 1.08(0.64-0.47) | 54 | 1(0.75-1.3) |
| **Diseases of Heart** | 133 | 2.72(2.27-3.22)* | 180 | 1.17(1.01-1.36)* | 128 | 1.04(0.86-1.23) | 104 | 1.28(1.04-1.52)* | 545 | 1.34(1.23-1.46)* |
| **Hypertension without Heart Disease** | 11 | 2.94(1.47-5.26)* | 15 | 1.21(0.68-2) | 16 | 1.5(0.86-2.43) | 12 | 1.6(0.83-0.86) | 54 | 1.57(1.18-2.05)* |
| **Cerebrovascular Diseases** | 25 | 1.93(1.25-2.85)* | 37 | 0.9(0.63-1.24) | 32 | 0.93(0.64-1.32) | 27 | 1.12(0.74-0.64) | 121 | 1.08(0.89-1.29) |
| **Atherosclerosis** | 1 | 1.88(0.05-10.47) | 0 | 0(0-2.49) | 2 | 1.8(0.22-6.52) | 2 | 2.83(0.34-0.22) | 5 | 1.31(0.42-3.05) |
| **Aortic Aneurysm and Dissection** | 0 | 0(0-5.59) | 2 | 0.99(0.12-3.57) | 0 | 0(0-2.44) | 0 | 0(0-0) | 2 | 0.39(0.05-1.41) |
| **Other Diseases of Arteries, Arterioles, Capillaries** | 3 | 3.12(0.64-9.12) | 5 | 1.64(0.53-3.83) | 3 | 1.19(0.24-3.47) | 1 | 0.59(0.01-0.24) | 12 | 1.45(0.75-2.54) |
| **Pneumonia and Influenza** | 6 | 1.69(0.62-3.67) | 17 | 1.49(0.87-2.39) | 13 | 1.37(0.73-2.34) | 10 | 1.58(0.76-0.73) | 46 | 1.49(1.09-1.99)* |
| **Chronic Obstructive Pulmonary Disease and Allied Cond** | 11 | 1.83(0.91-3.27) | 22 | 1.05(0.66-1.59) | 10 | 0.56(0.27-1.02) | 13 | 1.09(0.58-0.27) | 56 | 0.99(0.75-1.28) |
| **Stomach and Duodenal Ulcers** | 0 | 0(0-19.56) | 1 | 1.71(0.04-9.51) | 1 | 2.27(0.06-12.67) | 0 | 0(0-0.06) | 2 | 1.35(0.16-4.86) |
| **Chronic Liver Disease and Cirrhosis** | 0 | 0(0-3.17) | 0 | 0.00(0-0.97)* | 1 | 0.38(0.01-2.14) | 0 | 0(0-0.01) | 1 | 0.11(0-0.63)* |
| **Nephritis, Nephrotic Syndrome and Nephrosis** | 15 | 2.36(1.32-3.89)* | 35 | 1.68(1.17-2.33)* | 25 | 1.45(0.94-2.14) | 20 | 1.82(1.11-1.85)* | 95 | 1.71(1.39-2.09)* |
| **Complications of Pregnancy, Childbirth, Puerperium** | 0 | 0(0-133.55) | 1 | 11.11(0.28-61.88) | 0 | 0(0-72.69) | 0 | 0(0-0) | 1 | 5.3(0.13-29.52) |
| **Congenital Anomalies** | 0 | 0(0-23.2) | 0 | 0(0-7.2) | 0 | 0(0-10.13) | 0 | 0(0-0) | 0 | 0(0-2.99) |
| **Certain Conditions Originating in Perinatal Period** | 0 | 0(0-9291.93) | 0 | 0(0-2,363.43) | 0 | 0(0-2,769.88) | 0 | 0(0-0) | 0 | 0(0-834.04) |
| **Symptoms, Signs and Ill-Defined Conditions** | 3 | 1.51(0.31-4.41) | 16 | 2.53(1.45-4.11)* | 7 | 1.25(0.5-2.58) | 2 | 0.56(0.07-0.5) | 28 | 1.60(1.07-2.32)* |
| **Accidents and Adverse Effects** | 4 | 1.34(0.36-3.42) | 9 | 0.88(0.4-1.67) | 10 | 1.25(0.6-2.3) | 4 | 0.79(0.22-0.6) | 27 | 1.03(0.68-1.49) |
| **Suicide and Self-Inflicted Injury** | 0 | 0(0-23.44) | 0 | 0(0-7.01) | 0 | 0(0-10.7) | 0 | 0(0-0) | 0 | 0(0-3.09) |
| **Homicide and Legal Intervention** | 1 | 3.15(0.08-17.53) | 2 | 2.01(0.24-7.28) | 1 | 1.61(0.04-8.99) | 0 | 0(0-0.04) | 4 | 1.8(0.49-4.6) |
| **Other Cause of Death** | 66 | 2.65(2.05-3.37)* | 107 | 1.26(1.03-1.52)* | 79 | 1.02(0.8-1.27) | 72 | 1.26(0.99-0.8) | 324 | 1.32(1.18-1.48)* |

Abbreviation: SMR, standardized mortality ratio. CI: confidence interval

Values indicate the number of patients with cancer who died from each cause of death.

* P value less than .05

**Supporting Table 7.** Standardized-mortality ratios (SMRs) for each cause of death following Endometrial Cancer diagnosis in Non-Hispanic American Indian/Alaska Native.

| **Timing of deaths after diagnosis** | | | | | | | | | | |
| --- | --- | --- | --- | --- | --- | --- | --- | --- | --- | --- |
|  | **＜1year** | | **1-5years** | | **5-10years** | | **＞10years** | | **Total** | |
| **Causes of death** | **No.observed** | **SMR(95% CI)** | **No.observed** | **SMR(95% CI)** | **No.observed** | **SMR(95% CI)** | **No.observed** | **SMR(95% CI)** | **No.observed** | **SMR(95% CI)** |
| **All Causes of Death** | 52 | 12.42(9.27-16.28)* | 91 | 5.01(4.04-6.15)* | 63 | 4.08(3.13-5.21)* | 33 | 2.99(2.06-4.19)* | 239 | 4.89(4.29-5.55)* |
| **Endometrial Cancer deaths** | 33 | 477.85(328.93-671.08)* | 42 | 142.93(103.01-193.2)* | 9 | 36.85(16.85-69.96)* | 3 | 19.87(4.1-58.06)* | 87 | 114.76(91.92-141.55)* |
| **Other cancer causes of deaths** | 7 | 6.02(2.42-12.4)* | 19 | 3.88(2.34-6.06)* | 14 | 3.41(1.87-5.73)* | 5 | 1.87(0.61-4.36) | 45 | 3.31(5.56-4.69)* |
| **Non-cancer causes of deaths** | 12 | 4.06(2.1-7.09)* | 30 | 2.31(1.56-3.3)* | 40 | 3.60(2.57-4.9)* | 25 | 3.04(1.97-4.49)* | 107 | 3.03(2.49-3.67)* |
| **In situ, benign or unknown behavior neoplasm** | 0 | 0(0-144.89) | 0 | 0(0-33.17) | 0 | 0(0-37.64) | 0 | 0(0-53.4) | 0 | 0(0-12.14) |
| **Septicemia** | 0 | 0(0-62.58) | 0 | 0(0-14.35) | 0 | 0(0-16.92) | 1 | 6.64(0.17-36.99) | 1 | 1.16(1.04-8.14)* |
| **Other Infectious and Parasitic Diseases including HIV** | 1 | 18.77(0.48-104.59)* | 0 | 0(0-17.45) | 0 | 0(0-21.68) | 0 | 0(0-34.51) | 1 | 1.85(0.05-10.28) |
| **Diabetes Mellitus** | 2 | 9.70(1.17-35.04)* | 4 | 4.50(1.23-11.53)* | 4 | 5.30(1.44-13.56)* | 1 | 1.87(0.05-10.4) | 11 | 4.41(1.3-8.25)* |
| **Alzheimers (ICD-9 and 10 only)** | 0 | 0(0-37.44) | 0 | 0(0-7.12) | 0 | 0(0-7.33) | 2 | 4.2(0.51-15.16) | 2 | 1.15(2.15-4.52)* |
| **Diseases of Heart** | 2 | 2.3(0.28-8.3)* | 10 | 2.67(1.28-4.9)* | 11 | 3.51(1.75-6.28)* | 4 | 1.75(0.48-4.48) | 27 | 2.29(4.77-3.91)* |
| **Hypertension without Heart Disease** | 0 | 0(0-57.14) | 0 | 0(0-11.95) | 1 | 3.54(0.09-19.75) | 0 | 0(0-15.93) | 1 | 1.13(0.03-6.28) |
| **Cerebrovascular Diseases** | 0 | 0(0-10.9) | 0 | 0(0-2.56) | 4 | 3.32(0.91-8.51) | 2 | 2.24(0.27-8.08) | 6 | 1.15(2.57-3.37)* |
| **Atherosclerosis** | 0 | 0(0-383.49) | 0 | 0(0-93.26) | 0 | 0(0-120.98) | 1 | 45.64(1.16-254.31)* | 1 | 9.85(0.25-54.85)* |
| **Aortic Aneurysm and Dissection** | 0 | 0(0-185.77) | 0 | 0(0-44.87) | 0 | 0(0-55.13) | 0 | 0(0-79.87) | 0 | 0(0-17.14) |
| **Other Diseases of Arteries, Arterioles, Capillaries** | 0 | 0(0-273.11) | 0 | 0(0-63.47) | 0 | 0(0-76.67) | 0 | 0(0-103.72) | 0 | 0(0-23.75) |
| **Pneumonia and Influenza** | 0 | 0(0-32.28) | 1 | 1.95(0.05-10.87)* | 2 | 4.58(0.55-16.54) | 1 | 3.02(0.08-16.84) | 4 | 2.27(1.78-7.34)* |
| **Chronic Obstructive Pulmonary Disease and Allied Cond** | 0 | 0(0-27.8) | 1 | 1.71(0.04-9.53)* | 0 | 0(0-7.24) | 2 | 5.31(0.64-19.2) | 3 | 1.17(2.39-5.47)* |
| **Stomach and Duodenal Ulcers** | 0 | 0(0-476.9) | 1 | 31.25(0.79-174.12)* | 0 | 0(0-141.48) | 0 | 0(0-191.7) | 1 | 11.76(0.3-65.51) |
| **Chronic Liver Disease and Cirrhosis** | 2 | 27.93(3.38-100.91)* | 1 | 3.4(0.09-18.93)* | 0 | 0(0-15.36) | 2 | 14.10(1.71-50.92)* | 5 | 6.68(2.17-15.6)* |
| **Nephritis, Nephrotic Syndrome and Nephrosis** | 0 | 0(0-39.81) | 1 | 2.47(0.06-13.78)* | 1 | 2.79(0.07-15.53) | 3 | 11.46(2.36-33.48)* | 5 | 4.47(1.45-10.44)* |
| **Complications of Pregnancy, Childbirth, Puerperium** | 0 | 0(0-2901.47) | 0 | 0(0-838.48) | 0 | 0(0-1,446.98) | 0 | 0(0-6,275.35) | 0 | 0(0-418.81) |
| **Congenital Anomalies** | 0 | 0(0-630.45) | 0 | 0(0-160.28) | 0 | 0(0-210.26) | 0 | 0(0-378.32) | 0 | 0(0-65.68) |
| **Certain Conditions Originating in Perinatal Period** | 0 | 0(0-400124.69) | 0 | 0(0-79,747.01) | 0 | 0(0-126,195.18) | 0 | 0(0-549,899.41) | 0 | 0(0-40,352.48) |
| **Symptoms, Signs and Ill-Defined Conditions** | 0 | 0(0-108.13) | 1 | 6.68(0.17-37.22)* | 1 | 7.9(0.2-44) | 0 | 0(0-41.65) | 2 | 5.01(0.61-18.11) |
| **Accidents and Adverse Effects** | 0 | 0(0-26.98) | 1 | 1.74(0.04-9.71)* | 3 | 6.42(1.32-18.77)* | 1 | 3.2(0.08-17.83) | 5 | 3.36(1.09-7.83)* |
| **Suicide and Self-Inflicted Injury** | 0 | 0(0-121.97) | 0 | 0(0-31.72) | 0 | 0(0-41.94) | 0 | 0(0-82.39) | 0 | 0(0-13.21) |
| **Homicide and Legal Intervention** | 0 | 0(0-318.34) | 0 | 0(0-88.92) | 0 | 0(0-124.1) | 0 | 0(0-246.85) | 0 | 0(0-37.74) |
| **Other Cause of Death** | 5 | 9.06(2.94-21.15)* | 9 | 3.55(1.62-6.74)* | 13 | 5.70(3.04-9.75)* | 5 | 2.84(0.92-6.63) | 32 | 4.49(5.07-6.34)* |

Abbreviation: SMR, standardized mortality ratio. CI: confidence interval

Values indicate the number of patients with cancer who died from each cause of death.

* P value less than .05

**Supporting Table 8.** Standardized-mortality ratios (SMRs) for each cause of death following Endometrial Cancer diagnosis in Non-Hispanic Asian or Pacific Islander.

| **Timing of deaths after diagnosis** | | | | | | | | | | |
| --- | --- | --- | --- | --- | --- | --- | --- | --- | --- | --- |
|  | **＜1year** | | **1-5years** | | **5-10years** | | **＞10years** | | **Total** | |
| **Causes of death** | **No.observed** | **SMR(95% CI)** | **No.observed** | **SMR(95% CI)** | **No.observed** | **SMR(95% CI)** | **No.observed** | **SMR(95% CI)** | **No.observed** | **SMR(95% CI)** |
| **All Causes of Death** | 605 | 9.95(9.17-10.77)* | 1245 | 4.91(4.64-5.19)* | 498 | 1.95(1.78-2.13)* | 331 | 1.65(1.47-1.83)* | 2679 | 3.48(3.35-3.61)* |
| **Endometrial Cancer deaths** | 444 | 447.99(407.29-491.67)* | 793 | 193.18(179.96-207.1)* | 169 | 45.30(38.73-52.67)* | 29 | 11.70(7.84-16.8)* | 1435 | 126.93(120.45-133.67)* |
| **Other cancer causes of deaths** | 65 | 3.84(2.96-4.89)* | 150 | 2.17(1.83-2.54)* | 109 | 1.68(1.38-2.02)* | 63 | 1.39(1.07-1.78)* | 387 | 1.97(1.78-2.18)* |
| **Non-cancer causes of deaths** | 96 | 2.24(1.81-2.73)* | 302 | 1.67(1.49-1.87)* | 220 | 1.18(1.03-1.35)* | 239 | 1.56(1.37-1.77)* | 857 | 1.52(1.42-1.63)* |
| **In situ, benign or unknown behavior neoplasm** | 0 | 0(0-9.91) | 2 | 1.27(0.15-4.6) | 4 | 2.49(0.68-6.37) | 2 | 1.62(0.2-5.85) | 8 | 1.67(0.72-3.29) |
| **Septicemia** | 5 | 5.78(1.88-13.48)* | 9 | 2.49(1.14-4.72)* | 7 | 1.97(0.79-4.06) | 5 | 1.88(0.61-4.39) | 26 | 2.43(1.59-3.56)* |
| **Other Infectious and Parasitic Diseases including HIV** | 0 | 0(0-4.77) | 9 | 2.97(1.36-5.63)* | 3 | 1.1(0.23-3.21) | 4 | 2.2(0.6-5.64) | 16 | 1.92(1.09-3.11)* |
| **Diabetes Mellitus** | 6 | 1.99(0.73-4.33) | 15 | 1.2(0.67-1.98) | 14 | 1.14(0.62-1.91) | 17 | 1.80(1.05-2.89)* | 52 | 1.40(1.04-1.83)* |
| **Alzheimers (ICD-9 and 10 only)** | 2 | 1.46(0.18-5.26) | 6 | 0.86(0.31-1.87) | 6 | 0.65(0.24-1.41) | 15 | 1.47(0.82-2.42) | 29 | 1.04(0.7-1.5) |
| **Diseases of Heart** | 30 | 2.35(1.59-3.36)* | 73 | 1.39(1.09-1.75)* | 63 | 1.19(0.91-1.52) | 65 | 1.52(1.17-1.94)* | 231 | 1.43(1.26-1.63)* |
| **Hypertension without Heart Disease** | 0 | 0(0-3.96) | 8 | 1.88(0.81-3.7) | 6 | 1.23(0.45-2.68) | 7 | 1.57(0.63-3.23) | 21 | 1.44(0.89-2.21) |
| **Cerebrovascular Diseases** | 8 | 1.61(0.7-3.17) | 28 | 1.38(0.92-2) | 20 | 0.98(0.6-1.52) | 29 | 1.74(1.16-2.49)* | 85 | 1.36(1.09-1.69)* |
| **Atherosclerosis** | 1 | 6.95(0.18-38.71) | 1 | 1.81(0.05-10.08) | 1 | 1.89(0.05-10.54) | 0 | 0(0-8.58) | 3 | 1.81(0.37-5.3) |
| **Aortic Aneurysm and Dissection** | 1 | 3.43(0.09-19.11) | 1 | 0.86(0.02-4.8) | 0 | 0(0-3.31) | 2 | 2.44(0.3-8.8) | 4 | 1.18(0.32-3.02) |
| **Other Diseases of Arteries, Arterioles, Capillaries** | 0 | 0(0-19.07) | 1 | 1.23(0.03-6.84) | 1 | 1.24(0.03-6.92) | 0 | 0(0-5.52) | 2 | 0.81(0.1-2.91) |
| **Pneumonia and Influenza** | 1 | 0.6(0.02-3.33) | 5 | 0.7(0.23-1.64) | 6 | 0.8(0.29-1.73) | 6 | 0.95(0.35-2.06) | 18 | 0.79(0.47-1.26) |
| **Chronic Obstructive Pulmonary Disease and Allied Cond** | 2 | 1.04(0.13-3.74) | 6 | 0.73(0.27-1.59) | 2 | 0.23(0.03-0.85)* | 9 | 1.32(0.61-2.52)* | 19 | 0.75(0.45-1.17)* |
| **Stomach and Duodenal Ulcers** | 0 | 0(0-32.53) | 1 | 2.21(0.06-12.34) | 1 | 2.26(0.06-12.62) | 0 | 0(0-10.43) | 2 | 1.47(0.18-5.31) |
| **Chronic Liver Disease and Cirrhosis** | 0 | 0(0-3.6) | 3 | 0.72(0.15-2.12) | 4 | 1.12(0.3-2.86) | 2 | 0.89(0.11-3.2) | 9 | 0.82(0.37-1.55) |
| **Nephritis, Nephrotic Syndrome and Nephrosis** | 3 | 2.24(0.46-6.54) | 13 | 2.28(1.22-3.9)* | 8 | 1.35(0.58-2.67) | 7 | 1.48(0.6-3.06) | 31 | 1.76(1.19-2.49)* |
| **Complications of Pregnancy, Childbirth, Puerperium** | 0 | 0(0-272.69) | 1 | 22.59(0.57-125.88) | 0 | 0(0-166.59) | 0 | 0(0-627.89) | 1 | 11.65(0.3-64.93) |
| **Congenital Anomalies** | 1 | 12.31(0.31-68.6) | 1 | 3.12(0.08-17.41) | 0 | 0(0-14.17) | 0 | 0(0-23.64) | 2 | 2.45(0.3-8.84) |
| **Certain Conditions Originating in Perinatal Period** | 0 | 0(0-52065.03) | 0 | 0(0-8,875.43) | 0 | 0(0-14,175.12) | 0 | 0(0-59,862.94) | 0 | 0(0-4,563.54) |
| **Symptoms, Signs and Ill-Defined Conditions** | 0 | 0(0-7.54) | 6 | 2.95(1.08-6.43)* | 0 | 0(0-1.72) | 0 | 0(0-2.17) | 6 | 0.94(0.35-2.05) |
| **Accidents and Adverse Effects** | 2 | 1.03(0.12-3.71) | 3 | 0.38(0.08-1.11) | 6 | 0.82(0.3-1.78) | 6 | 1.1(0.4-2.4) | 17 | 0.75(0.44-1.2) |
| **Suicide and Self-Inflicted Injury** | 0 | 0(0-8.83) | 0 | 0(0-2.32) | 0 | 0(0-2.98) | 3 | 4.41(0.91-12.87) | 3 | 0.76(0.16-2.23) |
| **Homicide and Legal Intervention** | 0 | 0(0-23.91) | 2 | 3.58(0.43-12.92) | 0 | 0(0-8.71) | 0 | 0(0-15.94) | 2 | 1.46(0.18-5.28) |
| **Other Cause of Death** | 15 | 1.89(1.06-3.11)* | 50 | 1.43(1.06-1.89)* | 42 | 1.09(0.79-1.48) | 44 | 1.33(0.97-1.78) | 151 | 1.32(1.12-1.55)* |

Abbreviation: SMR, standardized mortality ratio. CI: confidence interval

Values indicate the number of patients with cancer who died from each cause of death.

* P value less than .05

**Supporting Table 9.** Standardized-mortality ratios (SMRs) for each cause of death following Endometrial Cancer diagnosis in Hispanic (All Races).

| **Timing of deaths after diagnosis** | | | | | | | | | | |
| --- | --- | --- | --- | --- | --- | --- | --- | --- | --- | --- |
|  | **＜1year** | | **1-5years** | | **5-10years** | | **＞10years** | | **Total** | |
| **Causes of death** | **No.observed** | **SMR(95% CI)** | **No.observed** | **SMR(95% CI)** | **No.observed** | **SMR(95% CI)** | **No.observed** | **SMR(95% CI)** | **No.observed** | **SMR(95% CI)** |
| **All Causes of Death** | 926 | 6.27(5.87-6.69)* | 1952 | 3.28(3.14-3.43)* | 867 | 1.50(1.4-1.6)* | 564 | 1.26(1.16-1.37)* | 4309 | 2.44(2.37-2.51)* |
| **Endometrial Cancer deaths** | 647 | 344.51(318.47-372.11)* | 1183 | 155.64(146.9-164.77)* | 225 | 33.65(29.4-38.35)* | 44 | 10.00(7.27-13.42)* | 2099 | 102.06(97.74-106.52)* |
| **Other cancer causes of deaths** | 116 | 2.87(2.37-3.44)* | 320 | 2.02(1.8-2.25)* | 189 | 1.35(1.16-1.55)* | 98 | 1.04(0.85-1.27) | 723 | 1.67(1.55-1.79)* |
| **Non-cancer causes of deaths** | 163 | 1.55(1.32-1.8)* | 449 | 1.05(0.95-1.15) | 453 | 1.05(0.95-1.15) | 422 | 1.21(1.1-1.33)* | 1487 | 1.13(1.08-1.19)* |
| **In situ, benign or unknown behavior neoplasm** | 3 | 3.49(0.72-10.2) | 4 | 1.16(0.32-2.97) | 3 | 0.89(0.18-2.6) | 2 | 0.78(0.09-2.8) | 12 | 1.17(0.6-2.04) |
| **Septicemia** | 3 | 1.29(0.27-3.77) | 9 | 0.95(0.43-1.8) | 6 | 0.66(0.24-1.43) | 7 | 1.04(0.42-2.14) | 25 | 0.9(0.59-1.33) |
| **Other Infectious and Parasitic Diseases including HIV** | 4 | 2.9(0.79-7.43) | 7 | 1.28(0.52-2.65) | 9 | 1.84(0.84-3.5) | 3 | 0.93(0.19-2.71) | 23 | 1.54(0.98-2.31) |
| **Diabetes Mellitus** | 13 | 2.85(1.52-4.87)* | 42 | 2.34(1.69-3.17)* | 43 | 2.63(1.9-3.54)* | 44 | 3.80(2.76-5.1)* | 142 | 2.82(2.37-3.32)* |
| **Alzheimers (ICD-9 and 10 only)** | 0 | 0.00(0-0.8)* | 12 | 0.56(0.29-0.98)* | 19 | 0.71(0.43-1.11) | 25 | 0.92(0.6-1.36) | 56 | 0.70(0.53-0.91)* |
| **Diseases of Heart** | 49 | 1.52(1.12-2.01)* | 119 | 0.95(0.78-1.13) | 130 | 1.05(0.87-1.24) | 107 | 1.08(0.88-1.3) | 405 | 1.06(0.96-1.17) |
| **Hypertension without Heart Disease** | 2 | 1.38(0.17-4.97) | 7 | 1.12(0.45-2.31) | 16 | 2.34(1.34-3.81)* | 10 | 1.68(0.81-3.1) | 35 | 1.71(1.19-2.38)* |
| **Cerebrovascular Diseases** | 8 | 0.95(0.41-1.88) | 32 | 0.97(0.66-1.37) | 30 | 0.9(0.61-1.29) | 40 | 1.44(1.03-1.97)* | 110 | 1.08(0.88-1.3) |
| **Atherosclerosis** | 0 | 0(0-7.76) | 2 | 1.23(0.15-4.43) | 1 | 0.68(0.02-3.78) | 2 | 1.83(0.22-6.63) | 5 | 1.07(0.35-2.5) |
| **Aortic Aneurysm and Dissection** | 0 | 0(0-6.28) | 0 | 0(0-1.66) | 0 | 0(0-1.83) | 1 | 0.68(0.02-3.81) | 1 | 0.16(0-0.89)* |
| **Other Diseases of Arteries, Arterioles, Capillaries** | 0 | 0(0-6.58) | 2 | 0.9(0.11-3.26) | 2 | 0.92(0.11-3.32) | 4 | 2.33(0.64-5.97) | 8 | 1.2(0.52-2.36) |
| **Pneumonia and Influenza** | 4 | 1.32(0.36-3.38) | 13 | 1.09(0.58-1.86) | 8 | 0.68(0.29-1.33) | 14 | 1.49(0.81-2.5) | 39 | 1.08(0.77-1.47) |
| **Chronic Obstructive Pulmonary Disease and Allied Cond** | 5 | 0.48(0.16-1.12) | 19 | 0.44(0.26-0.68)* | 11 | 0.26(0.13-0.46)* | 12 | 0.37(0.19-0.65)* | 47 | 0.36(0.27-0.48)* |
| **Stomach and Duodenal Ulcers** | 0 | 0(0-18.29) | 3 | 3.86(0.8-11.29) | 0 | 0(0-5.09) | 1 | 1.82(0.05-10.15) | 4 | 1.78(0.48-4.55) |
| **Chronic Liver Disease and Cirrhosis** | 5 | 2.5(0.81-5.84) | 16 | 1.97(1.13-3.2)* | 18 | 2.62(1.55-4.14)* | 7 | 1.64(0.66-3.37) | 46 | 2.16(1.58-2.89)* |
| **Nephritis, Nephrotic Syndrome and Nephrosis** | 7 | 2.79(1.12-5.76)* | 12 | 1.17(0.6-2.04) | 7 | 0.68(0.27-1.4) | 12 | 1.52(0.79-2.66) | 38 | 1.23(0.87-1.69) |
| **Complications of Pregnancy, Childbirth, Puerperium** | 0 | 0(0-126.07) | 2 | 20.17(2.44-72.87)* | 0 | 0(0-66.35) | 0 | 0(0-207.45) | 2 | 9.91(1.2-35.8)* |
| **Congenital Anomalies** | 0 | 0(0-14.21) | 0 | 0(0-3.69) | 0 | 0(0-4.66) | 0 | 0(0-7.72) | 0 | 0(0-1.46) |
| **Certain Conditions Originating in Perinatal Period** | 0 | 0(0-3674.31) | 0 | 0(0-958.15) | 0 | 0(0-1,214.11) | 0 | 0(0-2,131.72) | 0 | 0(0-383.35) |
| **Symptoms, Signs and Ill-Defined Conditions** | 2 | 1.19(0.14-4.32) | 3 | 0.45(0.09-1.32) | 5 | 0.72(0.23-1.68) | 5 | 0.96(0.31-2.24) | 15 | 0.73(0.41-1.21) |
| **Accidents and Adverse Effects** | 4 | 0.85(0.23-2.16) | 17 | 0.88(0.51-1.41) | 14 | 0.8(0.44-1.35) | 5 | 0.39(0.13-0.92)* | 40 | 0.74(0.53-1.01) |
| **Suicide and Self-Inflicted Injury** | 0 | 0(0-3.46) | 1 | 0.25(0.01-1.37) | 0 | 0(0-1.23) | 0 | 0(0-2.38) | 1 | 0.10(0-0.58)* |
| **Homicide and Legal Intervention** | 0 | 0(0-15.52) | 1 | 1.17(0.03-6.52) | 0 | 0(0-6.05) | 1 | 3.16(0.08-17.6) | 2 | 0.99(0.12-3.58) |
| **Other Cause of Death** | 30 | 1.38(0.93-1.97) | 72 | 0.77(0.6-0.97)* | 92 | 0.92(0.74-1.13) | 88 | 1.04(0.83-1.28) | 282 | 0.94(0.84-1.06) |

Abbreviation: SMR, standardized mortality ratio. CI: confidence interval

Values indicate the number of patients with cancer who died from each cause of death.

* P value less than .05

**Supporting Table 10.** Standardized-mortality ratios (SMRs) for each cause of death following localized Endometrial Cancer diagnosis.

| **Timing of deaths after diagnosis** | | | | | | | | | | |
| --- | --- | --- | --- | --- | --- | --- | --- | --- | --- | --- |
|  | **＜1year** | | **1-5years** | | **5-10years** | | **＞10years** | | **Total** | |
| **Causes of death** | **No.observed** | **SMR(95% CI)** | **No.observed** | **SMR(95% CI)** | **No.observed** | **SMR(95% CI)** | **No.observed** | **SMR(95% CI)** | **No.observed** | **SMR(95% CI)** |
| **All Causes of Death** | 1,494 | 1.40(1.33-1.47)* | 6,631 | 1.35(1.32-1.39)* | 5,925 | 1.08(1.06-1.11)* | 5,183 | 1.09(1.06-1.12)* | 19,233 | 1.19(1.17-1.2)* |
| **Endometrial Cancer deaths** | 630 | 52.58(48.55-56.85)* | 2,576 | 48.35(46.5-50.25)* | 839 | 15.58(14.54-16.67)* | 183 | 4.57(3.93-5.28)* | 4,228 | 26.57(25.77-27.38)* |
| **Other cancer causes of deaths** | 156 | 0.58(0.49-0.68)* | 1,221 | 1.05(0.99-1.11) | 1,178 | 1(0.94-1.06) | 857 | 0.96(0.9-1.03) | 3,412 | 0.97(0.94-1.01) |
| **Non-cancer causes of deaths** | 708 | 0.90(0.83-0.96)* | 2,834 | 0.77(0.74-0.8)* | 3,908 | 0.92(0.89-0.95)* | 4,143 | 1.08(1.05-1.12)* | 11,593 | 0.93(0.91-0.94)* |
| **In situ, benign or unknown behavior neoplasm** | 7 | 1.13(0.45-2.32) | 35 | 1.23(0.86-1.71) | 35 | 1.09(0.76-1.52) | 29 | 1.06(0.71-1.52) | 106 | 1.13(0.92-1.36) |
| **Septicemia** | 23 | 1.36(0.86-2.04) | 70 | 0.9(0.7-1.14) | 98 | 1.16(0.94-1.41) | 86 | 1.23(0.98-1.51) | 277 | 1.11(0.98-1.25) |
| **Other Infectious and Parasitic Diseases including HIV** | 10 | 1.09(0.52-2.01) | 33 | 0.8(0.55-1.12) | 54 | 1.24(0.93-1.62) | 40 | 1.24(0.88-1.68) | 137 | 1.08(0.91-1.28) |
| **Diabetes Mellitus** | 42 | 1.22(0.88-1.65) | 189 | 1.26(1.09-1.45)* | 200 | 1.30(1.13-1.5)* | 191 | 1.57(1.36-1.81)* | 622 | 1.35(1.25-1.46)* |
| **Alzheimers (ICD-9 and 10 only)** | 16 | 0.42(0.24-0.68)* | 92 | 0.44(0.36-0.54)* | 215 | 0.74(0.64-0.84)* | 359 | 1.11(0.99-1.23) | 682 | 0.79(0.73-0.85)* |
| **Diseases of Heart** | 263 | 1.01(0.89-1.14) | 929 | 0.80(0.75-0.85)* | 1,251 | 0.98(0.93-1.04) | 1,205 | 1.08(1.02-1.14)* | 3,648 | 0.96(0.93-0.99)* |
| **Hypertension without Heart Disease** | 9 | 0.76(0.35-1.44) | 55 | 0.93(0.7-1.21) | 83 | 1.14(0.9-1.41) | 99 | 1.41(1.15-1.72)* | 246 | 1.15(1.01-1.3)* |
| **Cerebrovascular Diseases** | 76 | 1.09(0.86-1.37) | 238 | 0.76(0.67-0.87)* | 292 | 0.84(0.75-0.94)* | 331 | 1.04(0.93-1.16) | 937 | 0.89(0.84-0.95)* |
| **Atherosclerosis** | 5 | 1.13(0.37-2.65) | 16 | 0.89(0.51-1.45) | 16 | 0.94(0.54-1.52) | 18 | 1.37(0.81-2.16) | 55 | 1.05(0.79-1.36) |
| **Aortic Aneurysm and Dissection** | 1 | 0.22(0.01-1.21) | 11 | 0.56(0.28-1) | 8 | 0.41(0.18-0.81)* | 12 | 0.77(0.4-1.35) | 32 | 0.54(0.37-0.76)* |
| **Other Diseases of Arteries, Arterioles, Capillaries** | 5 | 1.13(0.37-2.65) | 17 | 0.86(0.5-1.37) | 15 | 0.69(0.39-1.14) | 12 | 0.64(0.33-1.11) | 49 | 0.76(0.56-1)* |
| **Pneumonia and Influenza** | 12 | 0.49(0.25-0.85)* | 75 | 0.67(0.53-0.84)* | 124 | 1.01(0.84-1.2) | 110 | 1.03(0.85-1.25) | 321 | 0.88(0.78-0.98)* |
| **Chronic Obstructive Pulmonary Disease and Allied Cond** | 30 | 0.43(0.29-0.61)* | 122 | 0.37(0.31-0.44)* | 220 | 0.59(0.51-0.67)* | 219 | 0.68(0.59-0.77)* | 591 | 0.54(0.5-0.58)* |
| **Stomach and Duodenal Ulcers** | 0 | 0(0-2.39) | 11 | 1.67(0.83-2.99) | 7 | 1.03(0.41-2.12) | 5 | 0.87(0.28-2.04) | 23 | 1.11(0.71-1.67) |
| **Chronic Liver Disease and Cirrhosis** | 12 | 1.09(0.56-1.9) | 47 | 0.97(0.71-1.29) | 48 | 1.02(0.75-1.36) | 31 | 0.93(0.63-1.32) | 138 | 0.99(0.83-1.17) |
| **Nephritis, Nephrotic Syndrome and Nephrosis** | 17 | 0.86(0.5-1.37) | 90 | 0.97(0.78-1.2) | 113 | 1.08(0.89-1.3) | 116 | 1.32(1.09-1.58)* | 336 | 1.1(0.99-1.23) |
| **Complications of Pregnancy, Childbirth, Puerperium** | 0 | 0(0-43.43) | 0 | 0(0-12.21) | 0 | 0(0-19.37) | 0 | 0(0-54.6) | 0 | 0(0-5.72) |
| **Congenital Anomalies** | 1 | 0.7(0.02-3.93) | 3 | 0.51(0.1-1.49) | 2 | 0.37(0.04-1.34) | 4 | 1.07(0.29-2.74) | 10 | 0.61(0.29-1.12) |
| **Certain Conditions Originating in Perinatal Period** | 0 | 0(0-923.18) | 0 | 0(0-219.8) | 0 | 0(0-225.77) | 0 | 0(0-325.04) | 0 | 0(0-76.11) |
| **Symptoms, Signs and Ill-Defined Conditions** | 9 | 0.72(0.33-1.37) | 42 | 0.70(0.5-0.94)* | 65 | 0.88(0.68-1.12) | 42 | 0.69(0.5-0.94)* | 158 | 0.76(0.65-0.89)* |
| **Accidents and Adverse Effects** | 16 | 0.59(0.34-0.96)* | 83 | 0.66(0.52-0.82)* | 104 | 0.74(0.61-0.9)* | 103 | 0.83(0.68-1.01) | 306 | 0.73(0.65-0.82)* |
| **Suicide and Self-Inflicted Injury** | 1 | 0.22(0.01-1.21) | 7 | 0.37(0.15-0.76)* | 13 | 0.8(0.43-1.37) | 10 | 1.03(0.49-1.89) | 31 | 0.63(0.43-0.89)* |
| **Homicide and Legal Intervention** | 0 | 0(0-3.04) | 3 | 0.64(0.13-1.87) | 2 | 0.51(0.06-1.85) | 2 | 0.83(0.1-3.01) | 7 | 0.57(0.23-1.18) |
| **Other Cause of Death** | 139 | 0.89(0.75-1.05) | 565 | 0.73(0.67-0.79)* | 830 | 0.85(0.79-0.91)* | 971 | 1.04(0.98-1.11) | 2,505 | 0.88(0.85-0.92)* |

Abbreviation: SMR, standardized mortality ratio. CI: confidence interval

Values indicate the number of patients with cancer who died from each cause of death.

*P value less than .05

**Supporting Table 11.** Standardized-mortality ratios (SMRs) for each cause of death following regional Endometrial Cancer diagnosis.

| **Timing of deaths after diagnosis** | | | | | | | | | | |
| --- | --- | --- | --- | --- | --- | --- | --- | --- | --- | --- |
|  | **＜1year** | | **1-5years** | | **5-10years** | | **＞10years** | | **Total** | |
| **Causes of death** | **No.observed** | **SMR(95% CI)** | **No.observed** | **SMR(95% CI)** | **No.observed** | **SMR(95% CI)** | **No.observed** | **SMR(95% CI)** | **No.observed** | **SMR(95% CI)** |
| **All Causes of Death** | 2,029 | 5.69(5.44-5.94)* | 5,671 | 4.66(4.54-4.78)* | 2,067 | 1.97(1.88-2.05)* | 1,088 | 1.56(1.47-1.65)* | 10,855 | 3.27(3.2-3.33)* |
| **Endometrial Cancer deaths** | 1,457 | 387.78(368.12-408.21)* | 3,821 | 291.45(282.28-300.84)* | 718 | 67.90(63.02-73.05)* | 116 | 18.07(14.93-21.68)* | 6,112 | 180.50(176-185.09)* |
| **Other cancer causes of deaths** | 177 | 2.12(1.82-2.46)* | 695 | 2.46(2.28-2.65)* | 372 | 1.65(1.48-1.82)* | 174 | 1.27(1.09-1.48)* | 1,418 | 1.95(1.85-2.05)* |
| **Non-cancer causes of deaths** | 395 | 1.46(1.32-1.62)* | 1,155 | 1.25(1.18-1.33)* | 977 | 1.20(1.13-1.28)* | 798 | 1.44(1.34-1.54)* | 3,325 | 1.30(1.25-1.34)* |
| **In situ, benign or unknown behavior neoplasm** | 5 | 2.42(0.79-5.65) | 15 | 2.12(1.19-3.49)* | 9 | 1.47(0.67-2.79) | 12 | 2.98(1.54-5.21)* | 41 | 2.13(1.53-2.88)* |
| **Septicemia** | 19 | 3.30(1.99-5.16)* | 38 | 1.94(1.37-2.66)* | 28 | 1.70(1.13-2.46)* | 13 | 1.24(0.66-2.11) | 98 | 1.87(1.52-2.28)* |
| **Other Infectious and Parasitic Diseases including HIV** | 7 | 2.38(0.96-4.91) | 21 | 2.06(1.27-3.14)* | 14 | 1.68(0.92-2.81) | 8 | 1.63(0.7-3.21) | 50 | 1.89(1.41-2.5)* |
| **Diabetes Mellitus** | 28 | 2.43(1.62-3.52)* | 62 | 1.64(1.26-2.11)* | 57 | 1.90(1.44-2.46)* | 40 | 2.14(1.53-2.91)* | 187 | 1.91(1.64-2.2)* |
| **Alzheimers (ICD-9 and 10 only)** | 9 | 0.63(0.29-1.2) | 34 | 0.63(0.43-0.88)* | 76 | 1.34(1.06-1.68)* | 58 | 1.28(0.97-1.65) | 177 | 1.04(0.89-1.2) |
| **Diseases of Heart** | 134 | 1.50(1.26-1.78)* | 334 | 1.15(1.03-1.28)* | 285 | 1.17(1.03-1.31)* | 211 | 1.30(1.13-1.49)* | 964 | 1.23(1.15-1.31)* |
| **Hypertension without Heart Disease** | 6 | 1.38(0.51-3.01) | 26 | 1.69(1.1-2.47)* | 24 | 1.67(1.07-2.49)* | 24 | 2.34(1.5-3.48)* | 80 | 1.80(1.43-2.24)* |
| **Cerebrovascular Diseases** | 27 | 1.12(0.74-1.62) | 93 | 1.18(0.95-1.45) | 85 | 1.26(1.01-1.56)* | 73 | 1.59(1.24-1.99)* | 278 | 1.29(1.14-1.45)* |
| **Atherosclerosis** | 0 | 0(0-2.4) | 9 | 2.05(0.94-3.89) | 2 | 0.63(0.08-2.28) | 5 | 2.71(0.88-6.33) | 16 | 1.46(0.84-2.37) |
| **Aortic Aneurysm and Dissection** | 0 | 0(0-2.43) | 4 | 0.83(0.23-2.13) | 2 | 0.54(0.07-1.95) | 0 | 0(0-1.61) | 6 | 0.49(0.18-1.06) |
| **Other Diseases of Arteries, Arterioles, Capillaries** | 2 | 1.32(0.16-4.77) | 5 | 1(0.33-2.34) | 2 | 0.48(0.06-1.73) | 5 | 1.81(0.59-4.22) | 14 | 1.04(0.57-1.75) |
| **Pneumonia and Influenza** | 13 | 1.52(0.81-2.61) | 35 | 1.25(0.87-1.74) | 23 | 0.98(0.62-1.47) | 22 | 1.43(0.89-2.16) | 93 | 1.23(1-1.51) |
| **Chronic Obstructive Pulmonary Disease and Allied Cond** | 19 | 0.84(0.5-1.31) | 47 | 0.58(0.43-0.77)* | 31 | 0.44(0.3-0.62)* | 31 | 0.66(0.45-0.93)* | 128 | 0.58(0.48-0.69)* |
| **Stomach and Duodenal Ulcers** | 4 | 7.90(2.15-20.24)* | 2 | 1.24(0.15-4.49) | 2 | 1.54(0.19-5.58) | 2 | 2.36(0.29-8.51) | 10 | 2.35(1.13-4.32)* |
| **Chronic Liver Disease and Cirrhosis** | 5 | 1.57(0.51-3.67) | 9 | 0.79(0.36-1.5) | 10 | 1.1(0.53-2.02) | 7 | 1.29(0.52-2.66) | 31 | 1.07(0.72-1.51) |
| **Nephritis, Nephrotic Syndrome and Nephrosis** | 17 | 2.43(1.42-3.89)* | 52 | 2.19(1.63-2.87)* | 40 | 1.96(1.4-2.67)* | 29 | 2.21(1.48-3.17)* | 138 | 2.15(1.8-2.54)* |
| **Complications of Pregnancy, Childbirth, Puerperium** | 1 | 48.98(1.24-272.88)* | 0 | 0(0-56.89) | 1 | 28.34(0.72-157.92) | 0 | 0(0-334.51) | 2 | 15.20(1.84-54.91)* |
| **Congenital Anomalies** | 1 | 2.47(0.06-13.76) | 1 | 0.73(0.02-4.05) | 3 | 2.88(0.59-8.4) | 2 | 3.33(0.4-12.03) | 7 | 2.04(0.82-4.21) |
| **Certain Conditions Originating in Perinatal Period** | 0 | 0(0-3,403.28) | 0 | 0(0-941.39) | 0 | 0(0-1,136.65) | 0 | 0(0-1,981.26) | 0 | 0(0-364.88) |
| **Symptoms, Signs and Ill-Defined Conditions** | 4 | 0.91(0.25-2.33) | 26 | 1.72(1.12-2.52)* | 20 | 1.44(0.88-2.22) | 11 | 1.28(0.64-2.3) | 61 | 1.45(1.11-1.86)* |
| **Accidents and Adverse Effects** | 9 | 1.05(0.48-2) | 30 | 0.97(0.66-1.39) | 21 | 0.78(0.48-1.18) | 13 | 0.71(0.38-1.22) | 73 | 0.86(0.68-1.08) |
| **Suicide and Self-Inflicted Injury** | 1 | 0.82(0.02-4.57) | 2 | 0.47(0.06-1.68) | 4 | 1.26(0.34-3.23) | 3 | 1.82(0.38-5.32) | 10 | 0.97(0.46-1.78) |
| **Homicide and Legal Intervention** | 0 | 0(0-10.82) | 1 | 0.91(0.02-5.09) | 1 | 1.31(0.03-7.28) | 1 | 2.51(0.06-14) | 3 | 1.15(0.24-3.37) |
| **Other Cause of Death** | 68 | 1.26(0.98-1.6) | 228 | 1.16(1.01-1.32)* | 194 | 1.03(0.89-1.19) | 193 | 1.43(1.24-1.65)* | 683 | 1.19(1.1-1.28)* |

Abbreviation: SMR, standardized mortality ratio. CI: confidence interval

Values indicate the number of patients with cancer who died from each cause of death.

*P value less than .05

**Supporting Table 12.** Standardized-mortality ratios (SMRs) for each cause of death following distant Endometrial Cancer diagnosis.

| **Timing of deaths after diagnosis** | | | | | | | | | | |
| --- | --- | --- | --- | --- | --- | --- | --- | --- | --- | --- |
|  | **＜1year** | | **1-5years** | | **5-10years** | | **＞10years** | | **Total** | |
| **Causes of death** | **No.observed** | **SMR(95% CI)** | **No.observed** | **SMR(95% CI)** | **No.observed** | **SMR(95% CI)** | **No.observed** | **SMR(95% CI)** | **No.observed** | **SMR(95% CI)** |
| **All Causes of Death** | 2,886 | 35.77(34.48-37.1)* | 2,454 | 18.68(17.95-19.44)* | 270 | 3.99(3.53-4.5)* | 92 | 1.68(1.35-2.06)* | 5,702 | 17.04(16.61-17.49)* |
| **Endometrial Cancer deaths** | 2,354 | 2,607.41(2,503.13-2,714.91)* | 1,882 | 1,223.05(1,168.41-1,279.58)* | 148 | 193.91(163.92-227.78)* | 26 | 54.67(35.71-80.11)* | 4,410 | 1,198.24(1,163.13-1,234.14)* |
| **Other cancer causes of deaths** | 336 | 17.38(15.57-19.34)* | 391 | 12.12(10.95-13.38)* | 50 | 3.15(2.34-4.15)* | 20 | 1.94(1.19-3)* | 797 | 10.25(9.55-10.99)* |
| **Non-cancer causes of deaths** | 196 | 3.24(2.8-3.73)* | 181 | 1.86(1.59-2.15)* | 72 | 1.41(1.1-1.78)* | 46 | 1.04(0.76-1.39) | 495 | 1.96(1.79-2.14)* |
| **In situ, benign or unknown behavior neoplasm** | 5 | 10.73(3.49-25.05)* | 4 | 5.23(1.43-13.4)* | 0 | 0(0-9.22) | 0 | 0(0-11.76) | 9 | 4.63(2.12-8.79)* |
| **Septicemia** | 14 | 10.45(5.71-17.54)* | 10 | 4.64(2.22-8.53)* | 4 | 3.70(1.01-9.47)* | 2 | 2.48(0.3-8.95) | 30 | 5.57(3.76-7.95)* |
| **Other Infectious and Parasitic Diseases including HIV** | 2 | 2.92(0.35-10.57) | 1 | 0.87(0.02-4.84) | 0 | 0(0-6.4) | 1 | 2.62(0.07-14.58) | 4 | 1.43(0.39-3.67) |
| **Diabetes Mellitus** | 8 | 2.93(1.26-5.77)* | 7 | 1.64(0.66-3.37) | 5 | 2.47(0.8-5.76) | 2 | 1.42(0.17-5.12) | 22 | 2.11(1.32-3.19)* |
| **Alzheimers (ICD-9 and 10 only)** | 1 | 0.33(0.01-1.83) | 1 | 0.19(0-1.06) | 1 | 0.31(0.01-1.75) | 2 | 0.53(0.06-1.92) | 5 | 0.33(0.11-0.77)* |
| **Diseases of Heart** | 55 | 2.76(2.08-3.59)* | 40 | 1.31(0.94-1.79) | 20 | 1.33(0.81-2.05) | 11 | 0.85(0.42-1.52) | 126 | 1.61(1.34-1.91)* |
| **Hypertension without Heart Disease** | 2 | 2.01(0.24-7.25) | 3 | 1.86(0.38-5.44) | 2 | 2.32(0.28-8.39) | 2 | 2.47(0.3-8.9) | 9 | 2.1(0.96-3.99) |
| **Cerebrovascular Diseases** | 10 | 1.85(0.89-3.4) | 10 | 1.21(0.58-2.22) | 5 | 1.21(0.39-2.81) | 5 | 1.36(0.44-3.17) | 30 | 1.39(0.94-1.99) |
| **Atherosclerosis** | 1 | 3.06(0.08-17.06) | 2 | 4.55(0.55-16.42) | 0 | 0(0-19.74) | 0 | 0(0-24.64) | 3 | 2.72(0.56-7.95) |
| **Aortic Aneurysm and Dissection** | 1 | 2.89(0.07-16.1) | 1 | 1.89(0.05-10.51) | 0 | 0(0-14.94) | 0 | 0(0-20.89) | 2 | 1.54(0.19-5.56) |
| **Other Diseases of Arteries, Arterioles, Capillaries** | 3 | 8.75(1.81-25.58)* | 1 | 1.89(0.05-10.52) | 1 | 3.82(0.1-21.3) | 0 | 0(0-16.98) | 5 | 3.70(1.2-8.64)* |
| **Pneumonia and Influenza** | 3 | 1.61(0.33-4.69) | 10 | 3.45(1.65-6.34)* | 5 | 3.47(1.13-8.11)* | 0 | 0(0-3.03) | 18 | 2.42(1.44-3.83)* |
| **Chronic Obstructive Pulmonary Disease and Allied Cond** | 6 | 1.17(0.43-2.54) | 9 | 1.02(0.46-1.93) | 4 | 0.84(0.23-2.14) | 3 | 0.83(0.17-2.42) | 22 | 0.98(0.62-1.49) |
| **Stomach and Duodenal Ulcers** | 0 | 0(0-32.84) | 0 | 0(0-21.19) | 2 | 23.69(2.87-85.57)* | 1 | 15.17(0.38-84.51) | 3 | 6.87(1.42-20.07)* |
| **Chronic Liver Disease and Cirrhosis** | 1 | 1.38(0.03-7.7) | 1 | 0.75(0.02-4.19) | 0 | 0(0-5.45) | 1 | 2.45(0.06-13.65) | 3 | 0.96(0.2-2.79) |
| **Nephritis, Nephrotic Syndrome and Nephrosis** | 8 | 4.89(2.11-9.63)* | 8 | 3.09(1.33-6.09)* | 0 | 0(0-2.82) | 1 | 0.99(0.03-5.51) | 17 | 2.60(1.51-4.16)* |
| **Complications of Pregnancy, Childbirth, Puerperium** | 0 | 0(0-806.49) | 2 | 244.79(29.65-884.26)* | 0 | 0(0-1,166.38) | 0 | 0(0-3,467.94) | 2 | 117.85(14.27-425.71)* |
| **Congenital Anomalies** | 0 | 0(0-40.89) | 0 | 0(0-23.44) | 1 | 13.25(0.34-73.85) | 0 | 0(0-78.99) | 1 | 2.7(0.07-15.07) |
| **Certain Conditions Originating in Perinatal Period** | 0 | 0(0-15,491.93) | 0 | 0(0-8,408.08) | 0 | 0(0-14,588.78) | 0 | 0(0-24,146.87) | 0 | 0(0-3,407.83) |
| **Symptoms, Signs and Ill-Defined Conditions** | 7 | 7.35(2.96-15.15)* | 6 | 3.97(1.46-8.65)* | 0 | 0(0-4.42) | 1 | 1.44(0.04-8.04) | 14 | 3.51(1.92-5.89)* |
| **Accidents and Adverse Effects** | 4 | 2.12(0.58-5.42) | 0 | 0(0-1.1) | 0 | 0(0-2.05) | 3 | 2.05(0.42-5.99) | 7 | 0.82(0.33-1.69) |
| **Suicide and Self-Inflicted Injury** | 1 | 3.85(0.1-21.45) | 1 | 2(0.05-11.14) | 0 | 0(0-15) | 0 | 0(0-28.71) | 2 | 1.76(0.21-6.37) |
| **Homicide and Legal Intervention** | 0 | 0(0-46.83) | 0 | 0(0-27.97) | 0 | 0(0-62.06) | 0 | 0(0-120.1) | 0 | 0(0-12.26) |
| **Other Cause of Death** | 40 | 3.32(2.37-4.53)* | 33 | 1.61(1.11-2.26)* | 18 | 1.55(0.92-2.45) | 8 | 0.74(0.32-1.47) | 99 | 1.80(1.46-2.19)* |

Abbreviation: SMR, standardized mortality ratio. CI: confidence interval

Values indicate the number of patients with cancer who died from each cause of death.

*P value less than .05

**Supporting Table 13.** Standardized-mortality ratios (SMRs) for each cause of death following N0 Endometrial Cancer diagnosis.

| **Timing of deaths after diagnosis** | | | | | | | | | | |
| --- | --- | --- | --- | --- | --- | --- | --- | --- | --- | --- |
|  | **＜1year** | | **1-5years** | | **5-10years** | | **＞10years** | | **Total** | |
| **Causes of death** | **No.observed** | **SMR(95% CI)** | **No.observed** | **SMR(95% CI)** | **No.observed** | **SMR(95% CI)** | **No.observed** | **SMR(95% CI)** | **No.observed** | **SMR(95% CI)** |
| **All Causes of Death** | 2,147 | 3.27(3.13-3.41)* | 5,909 | 2.13(2.07-2.18)* | 2,130 | 1.39(1.33-1.45)* | - | - | 10,186 | 2.05(2.01-2.09)* |
| **Endometrial Cancer deaths** | 1,377 | 161.90(153.46-170.68)* | 3,164 | 88.85.78-92)* | 505 | 28.14(25.73-30.7)* | - | - | 5,046 | 81.30(79.07-83.58)* |
| **Other cancer causes of deaths** | 240 | 1.46(1.28-1.66)* | 897 | 1.35(1.26-1.44)* | 410 | 1.23(1.11-1.35)* | - | - | 1,547 | 1.33(1.26-1.4)* |
| **Non-cancer causes of deaths** | 530 | 1.10(1-1.19)* | 1,848 | 0.89(0.85-0.93)* | 1,215 | 1.03(0.97-1.09) | - | - | 3,593 | 0.96(0.93-0.99)* |
| **In situ, benign or unknown behavior neoplasm** | 5 | 1.31(0.43-3.06) | 26 | 1.63(1.07-2.39)* | 15 | 1.75(0.98-2.89) | - | - | 46 | 1.63(1.19-2.17)* |
| **Septicemia** | 31 | 2.85(1.93-4.04)* | 55 | 1.2(0.91-1.56) | 44 | 1.85(1.34-2.48)* | - | - | 130 | 1.62(1.35-1.92)* |
| **Other Infectious and Parasitic Diseases including HIV** | 9 | 1.42(0.65-2.7) | 29 | 1.2(0.81-1.73) | 13 | 1.17(0.62-1.99) | - | - | 51 | 1.23(0.91-1.61) |
| **Diabetes Mellitus** | 41 | 2.01(1.44-2.72)* | 127 | 1.51(1.26-1.8)* | 75 | 1.70(1.34-2.13)* | - | - | 243 | 1.63(1.44-1.85)* |
| **Alzheimers (ICD-9 and 10 only)** | 13 | 0.49(0.26-0.84)* | 62 | 0.48(0.36-0.61)* | 77 | 0.87(0.68-1.08) | - | - | 152 | 0.62(0.52-0.73)* |
| **Diseases of Heart** | 146 | 1.04(0.88-1.22) | 525 | 0.89(0.81-0.97)* | 351 | 1.05(0.94-1.17) | - | - | 1,022 | 0.96(0.9-1.02) |
| **Hypertension without Heart Disease** | 10 | 1.22(0.59-2.24) | 41 | 1.14(0.82-1.54) | 22 | 1.03(0.64-1.56) | - | - | 73 | 1.11(0.87-1.4) |
| **Cerebrovascular Diseases** | 40 | 1.07(0.76-1.46) | 132 | 0.82(0.68-0.97)* | 96 | 1.02(0.82-1.24) | - | - | 268 | 0.91(0.81-1.03) |
| **Atherosclerosis** | 1 | 0.59(0.01-3.29) | 5 | 0.79(0.26-1.84) | 2 | 0.65(0.08-2.34) | - | - | 8 | 0.72(0.31-1.42) |
| **Aortic Aneurysm and Dissection** | 1 | 0.45(0.01-2.52) | 1 | 0.11(0-0.61)* | 1 | 0.2(0.01-1.13) | - | - | 3 | 0.18(0.04-0.54)* |
| **Other Diseases of Arteries, Arterioles, Capillaries** | 2 | 0.81(0.1-2.91) | 12 | 1.14(0.59-1.99) | 5 | 0.85(0.28-1.98) | - | - | 19 | 1.1(0.6-1.57) |
| **Pneumonia and Influenza** | 13 | 0.97(0.51-1.65) | 45 | 0.79(0.58-1.06) | 33 | 0.07(0.73-1.5) | - | - | 91 | 0.9(0.72-1.1) |
| **Chronic Obstructive Pulmonary Disease and Allied Cond** | 25 | 0.55(0.36-0.81)* | 84 | 0.43(0.34-0.53)* | 52 | 0.48(0.36-0.63)* | - | - | 161 | 0.46(0.39-0.54)* |
| **Stomach and Duodenal Ulcers** | 0 | 0(0.0-4.68) | 6 | 1.77(0.65-3.85) | 0 | 0.0(0-1.91) | - | - | 6 | 0.98(0.36-2.14) |
| **Chronic Liver Disease and Cirrhosis** | 6 | 0.75(0.28-1.64) | 26 | 0.78(0.51-1.14) | 17 | 0.04(0.61-1.67) | - | - | 49 | 0.85(0.63-1.12) |
| **Nephritis, Nephrotic Syndrome and Nephrosis** | 15 | 1.21(0.67-1.99) | 61 | 1.17(0.89-1.5) | 37 | 1.29(0.91-1.78) | - | - | 113 | 1.21(0.1-1.46) |
| **Complications of Pregnancy, Childbirth, Puerperium** | 1 | 12.05(0.31-67.15) | 2 | 8.17(0.99-29.5) | 0 | 12.0(0-71.99) | - | - | 3 | 7.91(0.63-23.13)* |
| **Congenital Anomalies** | 1 | 1.11(0.03-6.16) | 1 | 0.27(0.01-1.5) | 3 | 1.7(0.35-4.97) | - | - | 5 | 0.78(0.25-1.83) |
| **Certain Conditions Originating in Perinatal Period** | 0 | 0(0.0-1,023.40) | 0 | 0(0-259.38) | 0 | 0.0(0-589.28) | - | - | 0 |  |
| **Symptoms, Signs and Ill-Defined Conditions** | 8 | 0.97(0.42-1.91) | 25 | 0.82(0.53-1.21) | 17 | 0.06(0.62-1.7) | - | - | 50 | 0.91(0.68-1.2) |
| **Accidents and Adverse Effects** | 13 | 0.68(0.36-1.17) | 75 | 0.92(0.73-1.16) | 42 | 0.96(0.69-1.3) | - | - | 130 | 0.9(0.75-1.07) |
| **Suicide and Self-Inflicted Injury** | 3 | 0.88(0.18-2.56) | 6 | 0.46(0.17-0.99)* | 4 | 0.72(0.2-1.86) | - | - | 13 | 0.59(0.31-1.01) |
| **Homicide and Legal Intervention** | 0 | 0(0.0-4.84) | 5 | 1.73(0.56-4.03) | 2 | 0.55(0.19-5.61) | - | - | 7 | 1.42(0.57-2.92) |
| **Other Cause of Death** | 119 | 1.07(0.89-1.28) | 390 | 0.80(0.72-0.89)* | 250 | 0.88(0.77-0.99)* | - | - | 759 | 0.86(0.8-0.92)* |

Abbreviation: SMR, standardized mortality ratio. CI: confidence interval

Values indicate the number of patients with cancer who died from each cause of death.

*P value less than .05

**Supporting Table 14.** Standardized-mortality ratios (SMRs) for each cause of death following N1 Endometrial Cancer diagnosis.

| **Timing of deaths after diagnosis** | | | | | | | | | | |
| --- | --- | --- | --- | --- | --- | --- | --- | --- | --- | --- |
|  | **＜1year** | | **1-5years** | | **5-10years** | | **＞10years** | | **Total** | |
| **Causes of death** | **No.observed** | **SMR(95% CI)** | **No.observed** | **SMR(95% CI)** | **No.observed** | **SMR(95% CI)** | **No.observed** | **SMR(95% CI)** | **No.observed** | **SMR(95% CI)** |
| **All Causes of Death** | 705 | 15.44(14.32-16.63)* | 1,101 | 8.00(7.54-8.49)* | 163 | 2.72(2.32-3.17)* | - | - | 1,969 | 8.10(7.75-8.47)* |
| **Endometrial Cancer deaths** | 557 | 917.61(842.98-997.08)* | 829 | 459.28(428.54-491.64)* | 97 | 136.82(110.95-166.91)* | - | - | 1,483 | 475.18(3-499.99)* |
| **Other cancer causes of deaths** | 69 | 6.04(4.7-7.64)* | 145 | 4.37(3.68-5.14)* | 23 | 1.76(1.11-2.64)* | - | - | 237 | 4.11(6-4.66)* |
| **Non-cancer causes of deaths** | 79 | 2.35(1.86-2.93)* | 127 | 1.24(1.03-1.47)* | 43 | 0.93(0.68-1.26) | - | - | 249 | 1.37(1.2-1.55)* |
| **In situ, benign or unknown behavior neoplasm** | 1 | 3.77(0.1-21.02) | 2 | 2.53(0.31-9.14) | 2 | 6(0.73-21.68) | - | - | 5 | 3.60(1.17-8.4)* |
| **Septicemia** | 7 | 8.92(3.59-18.39)* | 3 | 1.29(0.27-3.78)* | 1 | 1.06(0.03-5.93) | - | - | 11 | 2.72(1.36-4.87)* |
| **Other Infectious and Parasitic Diseases including HIV** | 2 | 4.53(0.55-16.37) | 4 | 3.31(0.9-8.47) | 0 | 0(0-8.41) | - | - | 6 | 2.87(1.05-6.25)* |
| **Diabetes Mellitus** | 1 | 0.67(0.02-3.75) | 5 | 1.16(0.38-2.7) | 2 | 1.14(0.14-4.1) | - | - | 8 | 1.06(0.46-2.08) |
| **Alzheimers (ICD-9 and 10 only)** | 0 | 0(0-2.05) | 3 | 0.48(0.1-1.4) | 1 | 0.29(0.01-1.61) | - | - | 4 | 0.35(0.09-0.89)* |
| **Diseases of Heart** | 22 | 2.23(1.4-3.38)* | 42 | 1.43(1.03-1.94)* | 12 | 0.92(0.47-1.61) | - | - | 76 | 1.45(1.15-1.82)* |
| **Hypertension without Heart Disease** | 2 | 3.4(0.41-12.27) | 1 | 0.55(0.01-3.06) | 1 | 1.17(0.03-6.54) | - | - | 4 | 1.23(0.33-3.14) |
| **Cerebrovascular Diseases** | 2 | 0.76(0.09-2.74) | 6 | 0.75(0.27-1.62) | 4 | 1.08(0.29-2.76) | - | - | 12 | 0.83(0.43-1.46) |
| **Atherosclerosis** | 0 | 0(0-31.85) | 0 | 0.0(0-11.84) | 0 | 0(0-30.74) | - | - | 0 | 0.0(0-6.74) |
| **Aortic Aneurysm and Dissection** | 0 | 0(0-23.81) | 0 | 0.0(0-8.1) | 0 | 0(0-19.17) | - | - | 0 | 0.0(0-4.6) |
| **Other Diseases of Arteries, Arterioles, Capillaries** | 0 | 0(0-20.91) | 0 | 0.0(0-6.97) | 1 | 4.3(0.11-23.96) | - | - | 1 | 1.07(0.03-5.94) |
| **Pneumonia and Influenza** | 2 | 2.17(0.26-7.84) | 4 | 1.43(0.39-3.66) | 1 | 0.83(0.02-4.62) | - | - | 7 | 1.42(0.57-2.93) |
| **Chronic Obstructive Pulmonary Disease and Allied Cond** | 3 | 0.96(0.2-2.81) | 3 | 0.31(0.06-0.91)* | 1 | 0.24(0.01-1.34) | - | - | 7 | 0.41(0.17-0.85)* |
| **Stomach and Duodenal Ulcers** | 0 | 0(0-68.47) | 0 | 0.0(0-22.22) | 1 | 13.32(0.34-74.2) | - | - | 1 | 3.39(0.09-18.89) |
| **Chronic Liver Disease and Cirrhosis** | 0 | 0(0-7.13) | 0 | 0.0(0-2.31) | 2 | 3.16(0.38-11.4) | - | - | 2 | 0.73(0.09-2.63) |
| **Nephritis, Nephrotic Syndrome and Nephrosis** | 1 | 1.09(0.03-6.06) | 3 | 1.12(0.23-3.26) | 3 | 2.62(0.54-7.67) | - | - | 7 | 1.47(0.59-3.04) |
| **Complications of Pregnancy, Childbirth, Puerperium** | 0 | 0(0-865.8) | 0 | 0.0(0-376.89) | 0 | 0(0-2,073.05) | - | - | 0 | 0.0(0-233.06) |
| **Congenital Anomalies** | 1 | 17.37(0.44-96.75) | 0 | 0.0(0-21.11) | 0 | 0(0-54.13) | - | - | 1 | 3.33(0.08-18.54) |
| **Certain Conditions Originating in Perinatal Period** | 0 | 0(0-17,141.47) | 0 | 0.0(0-5,772.15) | 0 | 0(0-15,103.19) | - | - | 0 | 0.0(0-3,358.01) |
| **Symptoms, Signs and Ill-Defined Conditions** | 2 | 3.58(0.43-12.94) | 3 | 2.01(0.41-5.88) | 0 | 0(0-5.9) | - | - | 5 | 1.87(0.61-4.36) |
| **Accidents and Adverse Effects** | 3 | 2.42(0.5-7.08) | 2 | 0.52(0.06-1.87) | 3 | 1.78(0.37-5.21) | - | - | 8 | 1.18(0.51-2.32) |
| **Suicide and Self-Inflicted Injury** | 0 | 0(0-18.18) | 0 | 0.0(0-6.19) | 0 | 0(0-17.46) | - | - | 0 | 0.0(0-3.65) |
| **Homicide and Legal Intervention** | 0 | 0(0-75.11) | 0 | 0.0(0-26.49) | 0 | 0(0-73.09) | - | - | 0 | 0.0(0-15.44) |
| **Other Cause of Death** | 25 | 3.27(2.11-4.82)* | 30 | 1.25(0.85-1.79)* | 6 | 0.54(0.2-1.17) | - | - | 61 | 1.43(1.09-1.84)* |

Abbreviation: SMR, standardized mortality ratio. CI: confidence interval

Values indicate the number of patients with cancer who died from each cause of death.

*P value less than .05

**Supporting Table 15.** Standardized-mortality ratios (SMRs) for each cause of death following N2 Endometrial Cancer diagnosis.

| **Timing of deaths after diagnosis** | | | | | | | | | | |
| --- | --- | --- | --- | --- | --- | --- | --- | --- | --- | --- |
|  | **＜1year** | | **1-5years** | | **5-10years** | | **＞10years** | | **Total** | |
| **Causes of death** | **No.observed** | **SMR(95% CI)** | **No.observed** | **SMR(95% CI)** | **No.observed** | **SMR(95% CI)** | **No.observed** | **SMR(95% CI)** | **No.observed** | **SMR(95% CI)** |
| **All Causes of Death** | 549 | 20.70(19-22.5)* | 837 | 11.41(10.65-12.21)* | 97 | 3.20(2.59-3.9)* | - | - | 1,483 | 11.39(10.82-11.98)* |
| **Endometrial Cancer deaths** | 441 | 1,134.08(1,030.69-1,245.03)* | 661 | 638,14.590.42-688.7)* | 46 | 117,51.86.03-156.74)* | - | - | 1,148 | 632,11.07-669.76)* |
| **Other cancer causes of deaths** | 57 | 8.07(6.11-10.46)* | 78 | 4.20(3.32-5.24)* | 16 | 2.29(1.31-3.72)* | - | - | 151 | 4.63(92-5.43)* |
| **Non-cancer causes of deaths** | 51 | 2.67(1.99-3.52)* | 98 | 1.82(1.48-2.22)* | 35 | 1.53(1.06-2.12)* | - | - | 184 | 1.92(65-2.22)* |
| **In situ, benign or unknown behavior neoplasm** | 4 | 25.93(7.07-66.39)* | 4 | 9.46(2.58-24.23)* | 1 | 5.85(0.15-32.61) | - | - | 9 | 12.04(5-22.85)* |
| **Septicemia** | 3 | 6.43(1.33-18.8)* | 6 | 4.76(1.75-10.37)* | 3 | 6.15(1.27-17.98)* | - | - | 12 | 5.42(8-9.47)* |
| **Other Infectious and Parasitic Diseases including HIV** | 0 | 0(0-13.73) | 2 | 3(0.36-10.84) | 0 | 0(0-16.06) | - | - | 2 | 1.72(0.21-6.2) |
| **Diabetes Mellitus** | 2 | 2.2(0.27-7.96) | 0 | 0(0-1.55) | 1 | 1.07(0.03-5.95) | - | - | 3 | 0.71(0.15-2.07) |
| **Alzheimers (ICD-9 and 10 only)** | 0 | 0(0-4.09) | 1 | 0.33(0.01-1.82) | 1 | 0.63(0.02-3.5) | - | - | 2 | 0.36(0.04-1.3) |
| **Diseases of Heart** | 14 | 2.52(1.38-4.23)* | 16 | 1.05(0.6-1.7) | 8 | 1.24(0.53-2.44) | - | - | 38 | 1.39(0.99-1.91) |
| **Hypertension without Heart Disease** | 0 | 0(0-11.14) | 3 | 3.19(0.66-9.33) | 1 | 2.4(0.06-13.37) | - | - | 4 | 2.37(0.65-6.07) |
| **Cerebrovascular Diseases** | 0 | 0(0-2.49) | 8 | 1.91(0.83-3.77) | 1 | 0.54(0.01-3.03) | - | - | 9 | 1.2(0.55-2.28) |
| **Atherosclerosis** | 0 | 0(0-59.68) | 1 | 6.41(0.16-35.7) | 0 | 0(0-16.06) | - | - | 1 | 3.64(0.09-20.29) |
| **Aortic Aneurysm and Dissection** | 0 | 0(0-40.56) | 0 | 0(0-15.09) | 1 | 10.07(0.25-56.1) | - | - | 1 | 2.3(0.06-12.82) |
| **Other Diseases of Arteries, Arterioles, Capillaries** | 1 | 9.99(0.25-55.64) | 0 | 0(0-13.27) | 0 | 1(1-16.16) | - | - | 1 | 2.02(0.05-11.28) |
| **Pneumonia and Influenza** | 2 | 3.87(0.47-13.98) | 6 | 4.12(1.51-8.97)* | 0 | 2(2-16.26) | - | - | 8 | 3.11(1.34-6.13)* |
| **Chronic Obstructive Pulmonary Disease and Allied Cond** | 1 | 0.54(0.01-3.02) | 3 | 0.57(0.12-1.67) | 3 | 1.39(0.29-4.06) | - | - | 7 | 0.76(0.3-1.56) |
| **Stomach and Duodenal Ulcers** | 0 | 0(0-117.19) | 0 | 0(0-41.42) | 0 | 0(0-16.06) | - | - | 0 | 0(0-23.19) |
| **Chronic Liver Disease and Cirrhosis** | 0 | 0(0-11.31) | 1 | 1.1(0.03-6.13) | 0 | 0(0-16.06) | - | - | 1 | 0.64(0.02-3.54) |
| **Nephritis, Nephrotic Syndrome and Nephrosis** | 1 | 1.85(0.05-10.33) | 6 | 4.15(1.52-9.04)* | 3 | 5.07(1.010-14.83)* | - | - | 10 | 3.88(1.86-7.14)* |
| **Complications of Pregnancy, Childbirth, Puerperium** | 0 | 0(0-1,606.45) | 0 | 0(0-746.08) | 0 | 0(0-16.06) | - | - | 0 | 0(0-452.41) |
| **Congenital Anomalies** | 0 | 0(0-103.57) | 0 | 0(0-37.56) | 0 | 0(0-16.06) | - | - | 0 | 0(0-21.74) |
| **Certain Conditions Originating in Perinatal Period** | 0 | 0(0-29,088.73) | 0 | 0(0-10,409.22) | 0 | 0(0-16.06) | - | - | 0 | 0(0-6,116.23) |
| **Symptoms, Signs and Ill-Defined Conditions** | 1 | 3.32(0.08-18.49) | 3 | 4.01(0.83-11.71) | 1 | 3.33(1.05-18.57) | - | - | 5 | 3.70(1.2-8.64)* |
| **Accidents and Adverse Effects** | 1 | 1.39(0.04-7.75) | 2 | 0.97(0.12-3.51) | 0 | 1(1-16.16) | - | - | 3 | 0.83(0.17-2.41) |
| **Suicide and Self-Inflicted Injury** | 2 | 15.89(1.92-57.39)* | 1 | 2.97(0.08-16.57) | 1 | 8.97(0.23-49.99) | - | - | 4 | 6.97(1.9-17.85)* |
| **Homicide and Legal Intervention** | 0 | 0(0-120.32) | 0 | 0(0-47.49) | 0 | 0(0-16.06) | - | - | 0 | 0(0-27.24) |
| **Other Cause of Death** | 11 | 2.57(1.28-4.6)* | 22 | 1.77(1.11-2.69)* | 7 | 1.28(0.51-2.63) | - | - | 40 | 1.81(1.29-2.46)* |

Abbreviation: SMR, standardized mortality ratio. CI: confidence interval

Values indicate the number of patients with cancer who died from each cause of death.

*P value less than .05

**Supporting Table 16.** Standardized-mortality ratios (SMRs) for each cause of death following Endometrial Cancer diagnosis in patients who tumor stage is Well differentiated; Grade I.

| **Timing of deaths after diagnosis** | | | | | | | | | | |
| --- | --- | --- | --- | --- | --- | --- | --- | --- | --- | --- |
|  | **＜1year** | | **1-5years** | | **5-10years** | | **＞10years** | | **Total** | |
| **Causes of death** | **No.observed** | **SMR(95% CI)** | **No.observed** | **SMR(95% CI)** | **No.observed** | **SMR(95% CI)** | **No.observed** | **SMR(95% CI)** | **No.observed** | **SMR(95% CI)** |
| **All Causes of Death** | 650 | 1.21(1.12-1.31)* | 2,817 | 1.10(1.06-1.15)* | 3083 | 1.05(1.01-1.08)* | 2,866 | 1.10(1.06-1.15)* | 9,416 | 1.09(1.07-1.11)* |
| **Endometrial Cancer deaths** | 269 | 41.49(36.68-46.76)* | 825 | 27.61(25.76-29.56)* | 380 | 12.25(11.05-13.55)* | 121 | 5.23(4.34-6.24)* | 1,595 | 17.62(16.76-18.51)* |
| **Other cancer causes of deaths** | 63 | 0.44(0.34-0.57)* | 541 | 0.85(0.78-0.92)* | 607 | 0.91(0.84-0.99)* | 502 | 0.99(0.91-1.08) | 1,713 | 0.88(0.84-0.92)* |
| **Non-cancer causes of deaths** | 318 | 0.82(0.73-0.92)* | 1,451 | 0.77(0.73-0.81)* | 2096 | 0.93(0.89-0.97)* | 2,243 | 1.09(1.04-1.13)* | 6,108 | 0.93(0.9-0.95)* |
| **In situ, benign or unknown behavior neoplasm** | 2 | 0.64(0.08-2.32) | 16 | 1.08(0.62-1.75) | 16 | 0.93(0.53-1.51) | 21 | 1.4(0.87-2.14) | 55 | 1.1(0.83-1.43) |
| **Septicemia** | 7 | 0.83(0.33-1.71) | 41 | 1.01(0.73-1.38) | 56 | 1.22(0.92-1.59) | 49 | 1.27(0.94-1.68) | 153 | 1.15(0.97-1.34) |
| **Other Infectious and Parasitic Diseases including HIV** | 4 | 0.82(0.22-2.1) | 21 | 0.93(0.57-1.42) | 31 | 1.28(0.87-1.82) | 24 | 1.33(0.85-1.97) | 80 | 1.15(0.91-1.43) |
| **Diabetes Mellitus** | 16 | 0.93(0.53-1.51) | 100 | 1.27(1.03-1.54)* | 120 | 1.43(1.19-1.71)* | 124 | 1.85(1.54-2.2)* | 360 | 1.46(1.31-1.62)* |
| **Alzheimers (ICD-9 and 10 only)** | 6 | 0.35(0.13-0.75)* | 39 | 0.39(0.28-0.53)* | 115 | 0.78(0.64-0.93)* | 185 | 1.08(0.93-1.25) | 345 | 0.79(0.71-0.88)* |
| **Diseases of Heart** | 118 | 0.95(0.78-1.13) | 468 | 0.80(0.73-0.88)* | 664 | 0.99(0.92-1.07) | 654 | 1.09(1.01-1.17)* | 1,904 | 0.96(0.92-1.01) |
| **Hypertension without Heart Disease** | 7 | 1.26(0.51-2.59) | 33 | 1.13(0.78-1.59) | 42 | 1.11(0.8-1.50) | 59 | 1.58(1.2-2.04)* | 141 | 1.28(1.08-1.51)* |
| **Cerebrovascular Diseases** | 38 | 1.15(0.81-1.57) | 121 | 0.78(0.64-0.93)* | 158 | 0.87(0.74-1.01) | 179 | 1.05(0.9-1.22) | 496 | 0.92(0.84-1) |
| **Atherosclerosis** | 4 | 1.98(0.54-5.07) | 8 | 0.92(0.4-1.82) | 8 | 0.92(0.4-1.80) | 12 | 1.72(0.89-3.01) | 32 | 1.21(0.83-1.71) |
| **Aortic Aneurysm and Dissection** | 1 | 0.44(0.01-2.45) | 3 | 0.30(0.06-0.87)* | 2 | 0.19(0.02-0.69)* | 4 | 0.47(0.13-1.2) | 10 | 0.32(0.15-0.59)* |
| **Other Diseases of Arteries, Arterioles, Capillaries** | 1 | 0.47(0.01-2.62) | 7 | 0.7(0.28-1.44) | 8 | 0.7(0.3-1.37) | 6 | 0.59(0.22-1.28) | 22 | 0.65(0.41-0.98)* |
| **Pneumonia and Influenza** | 5 | 0.43(0.14-0.99)* | 33 | 0.59(0.41-0.83)* | 60 | 0.93(0.71-1.20) | 48 | 0.84(0.62-1.11) | 146 | 0.77(0.65-0.91)* |
| **Chronic Obstructive Pulmonary Disease and Allied Cond** | 10 | 0.28(0.13-0.51)* | 68 | 0.39(0.3-0.49)* | 114 | 0.55(0.46-0.66)* | 117 | 0.65(0.54-0.78)* | 309 | 0.52(0.46-0.58)* |
| **Stomach and Duodenal Ulcers** | 0 | 0(0-4.8) | 4 | 1.17(0.32-2.99) | 4 | 1.09(0.3-2.78) | 3 | 0.95(0.2-2.78) | 11 | 1(0.5-1.78) |
| **Chronic Liver Disease and Cirrhosis** | 13 | 2.04(1.09-3.49)* | 27 | 0.94(0.62-1.37) | 30 | 1.06(0.71-1.51) | 16 | 0.8(0.46-1.3) | 86 | 1.03(0.82-1.27) |
| **Nephritis, Nephrotic Syndrome and Nephrosis** | 9 | 0.94(0.43-1.79) | 63 | 1.35(1.03-1.72)* | 64 | 1.16(0.89-1.48) | 75 | 1.58(1.24-1.98)* | 211 | 1.33(1.15-1.52)* |
| **Complications of Pregnancy, Childbirth, Puerperium** | 0 | 0(0-59.43) | 0 | 0(0-16.73) | 0 | 0(0-26.69) | 0 | 0(0-77.58) | 0 | 0(0-7.88) |
| **Congenital Anomalies** | 1 | 1.22(0.03-6.82) | 2 | 0.57(0.07-2.07) | 1 | 0.31(0.01-1.73) | 1 | 0.45(0.01-2.51) | 5 | 0.51(0.17-1.2) |
| **Certain Conditions Originating in Perinatal Period** | 0 | 0(0-1,482.92) | 0 | 0(0-346.19) | 0 | 0(0-356.44) | 0 | 0(0-530.76) | 0 | 0(0-121.17) |
| **Symptoms, Signs and Ill-Defined Conditions** | 2 | 0.33(0.04-1.2) | 15 | 0.50(0.28-0.82)* | 43 | 1.12(0.81-1.50) | 26 | 0.8(0.53-1.18) | 86 | 0.80(0.64-0.99)* |
| **Accidents and Adverse Effects** | 10 | 0.68(0.32-1.24) | 52 | 0.74(0.55-0.97)* | 46 | 0.59(0.43-0.78)* | 56 | 0.82(0.62-1.06) | 164 | 0.71(0.6-0.82)* |
| **Suicide and Self-Inflicted Injury** | 0 | 0(0-1.27) | 3 | 0.25(0.05-0.72)* | 11 | 1.05(0.53-1.89) | 9 | 1.47(0.67-2.79) | 23 | 0.73(0.46-1.09) |
| **Homicide and Legal Intervention** | 0 | 0(0-5.03) | 0 | 0(0-1.29) | 1 | 0.42(0.01-2.32) | 2 | 1.38(0.17-4.98) | 3 | 0.4(0.08-1.18) |
| **Other Cause of Death** | 58 | 0.75(0.57-0.98)* | 291 | 0.73(0.65-0.82)* | 440 | 0.85(0.77-0.94)* | 496 | 0.98(0.9-1.08) | 1,285 | 0.86(0.81-0.91)* |

Abbreviation: SMR, standardized mortality ratio. CI: confidence interval

Values indicate the number of patients with cancer who died from each cause of death.

*P value less than .05

**Supporting Table 17.** Standardized-mortality ratios (SMRs) for each cause of death following Endometrial Cancer diagnosis in patients who tumor stage is Moderately differentiated; Grade II

| **Timing of deaths after diagnosis** | | | | | | | | | | |
| --- | --- | --- | --- | --- | --- | --- | --- | --- | --- | --- |
|  | **＜1year** | | **1-5years** | | **5-10years** | | **＞10years** | | **Total** | |
| **Causes of death** | **No.observed** | **SMR(95% CI)** | **No.observed** | **SMR(95% CI)** | **No.observed** | **SMR(95% CI)** | **No.observed** | **SMR(95% CI)** | **No.observed** | **SMR(95% CI)** |
| **All Causes of Death** | 1,157 | 2.38(2.24-2.52)* | 4,058 | 1.91(1.85-1.97)* | 2,994 | 1.30(1.25-1.34)* | 2,304 | 1.17(1.12-1.22)* | 10,513 | 1.52(1.5-1.55)* |
| **Endometrial Cancer deaths** | 676 | 130.65(120.99-140.89)* | 2,047 | 92.52(88.55-96.61)* | 625 | 28.40(26.21-30.71)* | 122 | 7.48(6.21-8.93)* | 3,470 | 52.88(51.13-54.67)* |
| **Other cancer causes of deaths** | 117 | 0.99(0.82-1.18) | 622 | 1.25(1.16-1.36)* | 576 | 1.18(1.08-1.28)* | 374 | 1.02(0.92-1.13) | 1,689 | 1.15(1.09-1.2)* |
| **Non-cancer causes of deaths** | 364 | 1(0.9-1.11) | 1,389 | 0.86(0.82-0.91)* | 1,793 | 1(0.95-1.05) | 1,808 | 1.14(1.08-1.19)* | 5,354 | 1(0.97-1.03) |
| **In situ, benign or unknown behavior neoplasm** | 1 | 0.35(0.01-1.97) | 15 | 1.21(0.68-2) | 18 | 1.33(0.79-2.1) | 10 | 0.87(0.42-1.61) | 44 | 1.09(0.8-1.47) |
| **Septicemia** | 21 | 2.75(1.7-4.2)* | 42 | 1.25(0.9-1.7) | 51 | 1.43(1.07-1.88)* | 36 | 1.24(0.87-1.71) | 150 | 1.42(1.2-1.66)* |
| **Other Infectious and Parasitic Diseases including HIV** | 4 | 0.99(0.27-2.55) | 20 | 1.13(0.69-1.75) | 23 | 1.27(0.8-1.9) | 13 | 0.97(0.52-1.66) | 60 | 1.13(0.86-1.45) |
| **Diabetes Mellitus** | 25 | 1.62(1.05-2.39)* | 92 | 1.43(1.15-1.75)* | 95 | 1.48(1.2-1.81)* | 68 | 1.36(1.05-1.72)* | 280 | 1.44(1.28-1.62)* |
| **Alzheimers (ICD-9 and 10 only)** | 7 | 0.39(0.16-0.8)* | 54 | 0.58(0.43-0.75)* | 107 | 0.85(0.69-1.02) | 150 | 1.1(0.93-1.29) | 318 | 0.85(0.76-0.95)* |
| **Diseases of Heart** | 129 | 1.07(0.9-1.27) | 439 | 0.86(0.78-0.94)* | 555 | 1.02(0.94-1.11) | 520 | 1.11(1.02-1.21)* | 1,643 | 1(0.95-1.05) |
| **Hypertension without Heart Disease** | 4 | 0.73(0.2-1.88) | 28 | 1.08(0.72-1.56) | 39 | 1.26(0.89-1.72) | 38 | 1.3(0.92-1.79) | 109 | 1.19(0.98-1.44) |
| **Cerebrovascular Diseases** | 35 | 1.08(0.75-1.5) | 114 | 0.83(0.68-0.99)* | 150 | 1.01(0.86-1.19) | 145 | 1.09(0.92-1.29) | 444 | 0.98(0.89-1.08) |
| **Atherosclerosis** | 1 | 0.47(0.01-2.64) | 15 | 1.85(1.03-3.04)* | 8 | 1.08(0.47-2.14) | 8 | 1.45(0.63-2.86) | 32 | 1.38(0.95-1.95) |
| **Aortic Aneurysm and Dissection** | 0 | 0(0-1.73) | 7 | 0.81(0.33-1.67) | 7 | 0.84(0.34-1.74) | 7 | 1.08(0.43-2.23) | 21 | 0.82(0.51-1.26) |
| **Other Diseases of Arteries, Arterioles, Capillaries** | 4 | 1.97(0.54-5.04) | 11 | 1.26(0.63-2.26) | 4 | 0.43(0.12-1.11) | 7 | 0.89(0.36-1.84) | 26 | 0.93(0.61-1.37) |
| **Pneumonia and Influenza** | 6 | 0.52(0.19-1.14) | 40 | 0.81(0.58-1.1) | 55 | 1.05(0.79-1.37) | 53 | 1.2(0.9-1.56) | 154 | 0.98(0.83-1.14) |
| **Chronic Obstructive Pulmonary Disease and Allied Cond** | 20 | 0.63(0.38-0.97)* | 58 | 0.41(0.31-0.53)* | 92 | 0.58(0.47-0.71)* | 88 | 0.66(0.53-0.81)* | 258 | 0.55(0.49-0.62)* |
| **Stomach and Duodenal Ulcers** | 2 | 2.82(0.34-10.18) | 6 | 2.09(0.77-4.55) | 4 | 1.4(0.38-3.58) | 1 | 0.42(0.01-2.35) | 13 | 1.48(0.79-2.52) |
| **Chronic Liver Disease and Cirrhosis** | 3 | 0.64(0.13-1.87) | 19 | 0.96(0.58-1.5) | 21 | 1.11(0.69-1.7) | 16 | 1.19(0.68-1.93) | 59 | 1.04(0.79-1.34) |
| **Nephritis, Nephrotic Syndrome and Nephrosis** | 12 | 1.33(0.69-2.32) | 33 | 0.82(0.56-1.15) | 50 | 1.13(0.84-1.49) | 51 | 1.40(1.04-1.84)* | 146 | 1.12(0.95-1.32) |
| **Complications of Pregnancy, Childbirth, Puerperium** | 0 | 0(0-116.69) | 0 | 0(0-33.35) | 1 | 14.72(0.37-82.03) | 0 | 0(0-153.44) | 1 | 4.27(0.11-23.79) |
| **Congenital Anomalies** | 1 | 1.65(0.04-9.18) | 2 | 0.82(0.1-2.97) | 5 | 2.29(0.74-5.35) | 3 | 1.98(0.41-5.79) | 11 | 1.63(0.81-2.92) |
| **Certain Conditions Originating in Perinatal Period** | 0 | 0(0-2,242.52) | 0 | 0(0-551.4) | 0 | 0(0-574.55) | 0 | 0(0-817.07) | 0 | 0(0-191.43) |
| **Symptoms, Signs and Ill-Defined Conditions** | 6 | 1.04(0.38-2.27) | 27 | 1.02(0.67-1.48) | 25 | 0.79(0.51-1.16) | 21 | 0.83(0.51-1.27) | 79 | 0.88(0.7-1.1) |
| **Accidents and Adverse Effects** | 4 | 0.33(0.09-0.86)* | 32 | 0.60(0.41-0.85)* | 50 | 0.86(0.64-1.13) | 46 | 0.9(0.66-1.2) | 132 | 0.76(0.63-0.9)* |
| **Suicide and Self-Inflicted Injury** | 1 | 0.53(0.01-2.96) | 3 | 0.4(0.08-1.17) | 5 | 0.78(0.25-1.83) | 4 | 1.04(0.28-2.67) | 13 | 0.66(0.35-1.13) |
| **Homicide and Legal Intervention** | 0 | 0(0-7.27) | 1 | 0.53(0.01-2.95) | 2 | 1.29(0.16-4.65) | 1 | 1.04(0.03-5.8) | 4 | 0.81(0.22-2.09) |
| **Other Cause of Death** | 66 | 0.92(0.71-1.18) | 264 | 0.78(0.69-0.88)* | 372 | 0.90(0.81-0.99)* | 445 | 1.14(1.04-1.26)* | 1,147 | 0.95(0.89-1) |

Abbreviation: SMR, standardized mortality ratio. CI: confidence interval

Values indicate the number of patients with cancer who died from each cause of death.

*P value less than .05

**Supporting Table 18.** Standardized-mortality ratios (SMRs) for each cause of death following Endometrial Cancer diagnosis in patients who tumor stage is Poorly differentiated; Grade III

| **Timing of deaths after diagnosis** | | | | | | | | | | |
| --- | --- | --- | --- | --- | --- | --- | --- | --- | --- | --- |
|  | **＜1year** | | **1-5years** | | **5-10years** | | **＞10years** | | **Total** | |
| **Causes of death** | **No.observed** | **SMR(95% CI)** | **No.observed** | **SMR(95% CI)** | **No.observed** | **SMR(95% CI)** | **No.observed** | **SMR(95% CI)** | **No.observed** | **SMR(95% CI)** |
| **All Causes of Death** | 3,227 | 8.49(8.2-8.79)* | 5,858 | 4.68(4.56-4.8)* | 1,771 | 1.62(1.55-1.7)* | 1,013 | 1.26(1.19-1.34)* | 11,869 | 3.37(3.31-3.43)* |
| **Endometrial Cancer deaths** | 2,430 | 635.96(610.92-661.76)* | 3,940 | 315.14(305.37-325.13)* | 556 | 55.84(51.29-60.68)* | 75 | 11.81(9.29-14.8)* | 7,001 | 214.55(209.55-219.63)* |
| **Other cancer causes of deaths** | 343 | 4.00(3.59-4.45)* | 868 | 3.16(2.95-3.38)* | 338 | 1.54(1.38-1.71)* | 139 | 0.98(0.82-1.16) | 1,688 | 2.34(2.23-2.45)* |
| **Non-cancer causes of deaths** | 454 | 1.56(1.42-1.71)* | 1,050 | 1.09(1.02-1.16)* | 877 | 1.02(0.95-1.08) | 799 | 1.22(1.14-1.31)* | 3,180 | 1.15(1.11-1.19)* |
| **In situ, benign or unknown behavior neoplasm** | 10 | 4.55(2.18-8.37)* | 17 | 2.34(1.36-3.74)* | 6 | 0.94(0.35-2.05) | 7 | 1.52(0.61-3.14) | 40 | 1.96(1.4-2.67)* |
| **Septicemia** | 21 | 3.40(2.1-5.2)* | 21 | 1.04(0.64-1.59) | 20 | 1.17(0.72-1.81) | 14 | 1.18(0.65-1.99) | 76 | 1.38(1.08-1.72)* |
| **Other Infectious and Parasitic Diseases including HIV** | 9 | 3.01(1.38-5.72)* | 13 | 1.3(0.69-2.23) | 9 | 1.08(0.5-2.06) | 10 | 1.89(0.91-3.47) | 41 | 1.54(1.11-2.1)* |
| **Diabetes Mellitus** | 25 | 2.02(1.31-2.98)* | 53 | 1.36(1.02-1.78)* | 36 | 1.16(0.81-1.61) | 36 | 1.74(1.22-2.41)* | 150 | 1.46(1.23-1.71)* |
| **Alzheimers (ICD-9 and 10 only)** | 7 | 0.45(0.18-0.92)* | 26 | 0.44(0.29-0.64)* | 59 | 0.93(0.71-1.2) | 71 | 1.24(0.97-1.56) | 163 | 0.83(0.71-0.97)* |
| **Diseases of Heart** | 151 | 1.54(1.31-1.81)* | 320 | 1.03(0.92-1.15) | 284 | 1.08(0.96-1.21) | 223 | 1.15(1.01-1.32)* | 978 | 1.13(1.06-1.2)* |
| **Hypertension without Heart Disease** | 5 | 1.04(0.34-2.44) | 19 | 1.14(0.69-1.79) | 24 | 1.52(0.98-2.27) | 23 | 1.84(1.17-2.76)* | 71 | 1.43(1.12-1.8)* |
| **Cerebrovascular Diseases** | 32 | 1.2(0.82-1.7) | 89 | 1.05(0.84-1.29) | 53 | 0.73(0.54-0.95)* | 76 | 1.37(1.08-1.72)* | 250 | 1.04(0.92-1.18) |
| **Atherosclerosis** | 1 | 0.58(0.01-3.21) | 2 | 0.41(0.05-1.47) | 2 | 0.56(0.07-2.02) | 3 | 1.31(0.27-3.82) | 8 | 0.64(0.28-1.26) |
| **Aortic Aneurysm and Dissection** | 1 | 0.61(0.02-3.38) | 6 | 1.19(0.44-2.59) | 1 | 0.26(0.01-1.43) | 1 | 0.39(0.01-2.15) | 9 | 0.68(0.31-1.3) |
| **Other Diseases of Arteries, Arterioles, Capillaries** | 4 | 2.41(0.66-6.18) | 3 | 0.57(0.12-1.66) | 6 | 1.34(0.49-2.91) | 4 | 1.23(0.34-3.15) | 17 | 1.16(0.67-1.85) |
| **Pneumonia and Influenza** | 13 | 1.39(0.74-2.37) | 35 | 1.17(0.81-1.63) | 29 | 1.15(0.77-1.65) | 25 | 1.36(0.88-2.01) | 102 | 1.23(1-1.49)* |
| **Chronic Obstructive Pulmonary Disease and Allied Cond** | 16 | 0.67(0.39-1.1) | 42 | 0.52(0.37-0.7)* | 45 | 0.63(0.46-0.84)* | 40 | 0.77(0.55-1.05) | 143 | 0.63(0.53-0.74)* |
| **Stomach and Duodenal Ulcers** | 2 | 3.68(0.45-13.29) | 3 | 1.8(0.37-5.27) | 3 | 2.24(0.46-6.56) | 4 | 4.21(1.15-10.77)* | 12 | 2.67(1.38-4.66)* |
| **Chronic Liver Disease and Cirrhosis** | 2 | 0.67(0.08-2.41) | 8 | 0.81(0.35-1.6) | 6 | 0.77(0.28-1.68) | 6 | 1.23(0.45-2.68) | 22 | 0.86(0.54-1.31) |
| **Nephritis, Nephrotic Syndrome and Nephrosis** | 16 | 2.09(1.19-3.39)* | 42 | 1.66(1.2-2.25)* | 28 | 1.28(0.85-1.84) | 17 | 1.11(0.65-1.77) | 103 | 1.47(1.2-1.78)* |
| **Complications of Pregnancy, Childbirth, Puerperium** | 1 | 79.46(2.01-442.71)* | 2 | 55.75(6.75-201.37)* | 0 | 0(0-190.86) | 0 | 0(0-532.22) | 3 | 40.15(8.28-117.33)* |
| **Congenital Anomalies** | 0 | 0(0-9.63) | 0 | 0(0-3.06) | 0 | 0(0-4.08) | 2 | 3.56(0.43-12.84) | 2 | 0.65(0.08-2.36) |
| **Certain Conditions Originating in Perinatal Period** | 0 | 0(0-4,023.05) | 0 | 0(0-1,231.94) | 0 | 0(0-1,467.34) | 0 | 0(0-2,279.88) | 0 | 0(0-458.63) |
| **Symptoms, Signs and Ill-Defined Conditions** | 10 | 2.12(1.02-3.89)* | 24 | 1.49(0.96-2.22) | 16 | 1.04(0.6-1.69) | 5 | 0.48(0.15-1.11) | 55 | 1.18(0.89-1.53) |
| **Accidents and Adverse Effects** | 8 | 0.94(0.41-1.86) | 23 | 0.79(0.5-1.18) | 25 | 0.95(0.61-1.4) | 14 | 0.69(0.38-1.16) | 70 | 0.83(0.65-1.05) |
| **Suicide and Self-Inflicted Injury** | 2 | 1.99(0.24-7.21) | 2 | 0.61(0.07-2.22) | 1 | 0.42(0.01-2.35) | 0 | 0(0-2.81) | 5 | 0.63(0.2-1.47) |
| **Homicide and Legal Intervention** | 0 | 0(0-12.07) | 3 | 3.29(0.68-9.62) | 0 | 0(0-5.78) | 0 | 0(0-10.38) | 3 | 1.36(0.28-3.97) |
| **Other Cause of Death** | 89 | 1.55(1.24-1.9)* | 210 | 1.03(0.89-1.18) | 192 | 0.96(0.83-1.11) | 189 | 1.18(1.02-1.36)* | 680 | 1.09(1.01-1.18)* |

Abbreviation: SMR, standardized mortality ratio. CI: confidence interval

Values indicate the number of patients with cancer who died from each cause of death.

*P value less than .05

**Supporting Table 19.** Standardized-mortality ratios (SMRs) for each cause of death following Endometrial Cancer diagnosis in patients who tumor stage is Undifferentiated; anaplastic; Grade IV

| **Timing of deaths after diagnosis** | | | | | | | | | | |
| --- | --- | --- | --- | --- | --- | --- | --- | --- | --- | --- |
|  | **＜1year** | | **1-5years** | | **5-10years** | | **＞10years** | | **Total** | |
| **Causes of death** | **No.observed** | **SMR(95% CI)** | **No.observed** | **SMR(95% CI)** | **No.observed** | **SMR(95% CI)** | **No.observed** | **SMR(95% CI)** | **No.observed** | **SMR(95% CI)** |
| **All Causes of Death** | 1,375 | 12.98(12.31-13.69)* | 2,023 | 6.32(6.05-6.6)* | 414 | 1.72(1.56-1.9)* | 180 | 1.34(1.15-1.55)* | 3,992 | 4.98(4.83-5.14)* |
| **Endometrial Cancer deaths** | 1,066 | 915.94(861.77-972.62)* | 1,467 | 428.80(407.13-451.31)* | 144 | 64.80(54.65-76.29)* | 7 | 6.33(2.55-13.04)* | 2,684 | 339.18(326.47-352.26)* |
| **Other cancer causes of deaths** | 146 | 5.92(5-6.97)* | 276 | 3.88(3.44-4.37)* | 79 | 1.66(1.32-2.07)* | 36 | 1.49(1.04-2.06)* | 537 | 3.21(2.94-3.49)* |
| **Non-cancer causes of deaths** | 163 | 2.04(1.73-2.37)* | 280 | 1.14(1.01-1.28)* | 191 | 1(0.86-1.15) | 137 | 1.25(1.05-1.48)* | 771 | 1.23(1.15-1.32)* |
| **In situ, benign or unknown behavior neoplasm** | 4 | 6.51(1.77-16.66)* | 6 | 3.23(1.19-7.04)* | 4 | 2.87(0.78-7.35) | 3 | 3.91(0.81-11.42) | 17 | 3.67(2.14-5.88)* |
| **Septicemia** | 7 | 3.95(1.59-8.14)* | 14 | 2.66(1.45-4.46)* | 3 | 0.8(0.16-2.33) | 2 | 1(0.12-3.61) | 26 | 2.03(1.33-2.98)* |
| **Other Infectious and Parasitic Diseases including HIV** | 2 | 2.26(0.27-8.15) | 1 | 0.39(0.01-2.15) | 5 | 2.81(0.91-6.55) | 2 | 2.24(0.27-8.11) | 10 | 1.63(0.78-2.99) |
| **Diabetes Mellitus** | 12 | 3.41(1.76-5.95)* | 13 | 1.29(0.69-2.2) | 11 | 1.59(0.8-2.85) | 5 | 1.41(0.46-3.3) | 41 | 1.70(1.22-2.31)* |
| **Alzheimers (ICD-9 and 10 only)** | 6 | 1.38(0.51-3) | 8 | 0.52(0.22-1.02) | 11 | 0.76(0.38-1.36) | 13 | 1.35(0.72-2.32) | 38 | 0.87(0.61-1.19) |
| **Diseases of Heart** | 54 | 2.08(1.56-2.71)* | 76 | 0.99(0.78-1.24) | 53 | 0.92(0.69-1.2) | 30 | 0.93(0.63-1.33) | 213 | 1.11(0.96-1.27) |
| **Hypertension without Heart Disease** | 1 | 0.72(0.02-4.04) | 4 | 0.91(0.25-2.33) | 4 | 1.11(0.3-2.84) | 5 | 2.37(0.77-5.52) | 14 | 1.22(0.67-2.04) |
| **Cerebrovascular Diseases** | 8 | 1.13(0.49-2.23) | 17 | 0.81(0.47-1.29) | 21 | 1.3(0.8-1.98) | 9 | 0.97(0.44-1.84) | 55 | 1.03(0.77-1.34) |
| **Atherosclerosis** | 0 | 0(0-9.14) | 2 | 1.84(0.22-6.64) | 0 | 0(0-4.97) | 0 | 0(0-9.9) | 2 | 0.77(0.09-2.77) |
| **Aortic Aneurysm and Dissection** | 0 | 0(0-8.48) | 0 | 0(0-3.02) | 0 | 0(0-4.41) | 0 | 0(0-8.5) | 0 | 0(0-1.26) |
| **Other Diseases of Arteries, Arterioles, Capillaries** | 1 | 2.23(0.06-12.45) | 2 | 1.5(0.18-5.43) | 0 | 0(0-3.74) | 0 | 0(0-6.76) | 3 | 0.91(0.19-2.65) |
| **Pneumonia and Influenza** | 4 | 1.63(0.44-4.18) | 12 | 1.64(0.85-2.86) | 8 | 1.44(0.62-2.84) | 6 | 1.97(0.72-4.28) | 30 | 1.63(1.1-2.33)* |
| **Chronic Obstructive Pulmonary Disease and Allied Cond** | 9 | 1.32(0.61-2.51) | 10 | 0.48(0.23-0.88)* | 4 | 0.26(0.07-0.66)* | 8 | 0.92(0.4-1.82) | 31 | 0.60(0.4-0.85)* |
| **Stomach and Duodenal Ulcers** | 0 | 0(0-26.01) | 0 | 0(0-9.02) | 0 | 0(0-12.64) | 0 | 0(0-23.03) | 0 | 0(0-3.68) |
| **Chronic Liver Disease and Cirrhosis** | 0 | 0(0-4.15) | 3 | 1.14(0.24-3.34) | 1 | 0.6(0.02-3.33) | 1 | 1.19(0.03-6.62) | 5 | 0.83(0.27-1.94) |
| **Nephritis, Nephrotic Syndrome and Nephrosis** | 5 | 2.28(0.74-5.31) | 12 | 1.82(0.94-3.17) | 11 | 2.25(1.12-4.02)* | 3 | 1.15(0.24-3.37) | 31 | 1.90(1.29-2.7)* |
| **Complications of Pregnancy, Childbirth, Puerperium** | 0 | 0(0-1,008.66) | 0 | 0(0-460.6) | 0 | 0(0-1,069.04) | 0 | 0(0-3,263.58) | 0 | 0(0-227.05) |
| **Congenital Anomalies** | 0 | 0(0-34.43) | 0 | 0(0-12.04) | 0 | 0(0-19.19) | 0 | 0(0-38.07) | 0 | 0(0-5.25) |
| **Certain Conditions Originating in Perinatal Period** | 0 | 0(0-13,743.29) | 0 | 0(0-4,608.13) | 0 | 0(0-6,661.24) | 0 | 0(0-13,145.85) | 0 | 0(0-1,938.13) |
| **Symptoms, Signs and Ill-Defined Conditions** | 2 | 1.54(0.19-5.57) | 8 | 2.01(0.87-3.95) | 1 | 0.31(0.01-1.72) | 2 | 1.17(0.14-4.23) | 13 | 1.27(0.68-2.17) |
| **Accidents and Adverse Effects** | 7 | 2.90(1.17-5.98)* | 6 | 0.79(0.29-1.73) | 4 | 0.69(0.19-1.76) | 3 | 0.88(0.18-2.59) | 20 | 1.04(0.64-1.61) |
| **Suicide and Self-Inflicted Injury** | 0 | 0(0-12.68) | 2 | 2.4(0.29-8.68) | 0 | 0(0-7.51) | 0 | 0(0-16.13) | 2 | 1.08(0.13-3.92) |
| **Homicide and Legal Intervention** | 0 | 0(0-42.1) | 0 | 0(0-15.77) | 0 | 0(0-27.17) | 0 | 0(0-59.64) | 0 | 0(0-7.1) |
| **Other Cause of Death** | 34 | 2.05(1.42-2.87)* | 61 | 1.14(0.87-1.46) | 38 | 0.85(0.6-1.17) | 42 | 1.57(1.13-2.13)* | 175 | 1.24(1.06-1.43)* |

Abbreviation: SMR, standardized mortality ratio. CI: confidence interval

Values indicate the number of patients with cancer who died from each cause of death.

*P value less than .05

**Supporting Table 20.** Standardized-mortality ratios (SMRs) for each cause of death following Endometrial Cancer diagnosis in patients who received radiotherapy.

| **Timing of deaths after diagnosis** | | | | | | | | | | |
| --- | --- | --- | --- | --- | --- | --- | --- | --- | --- | --- |
|  | **＜1year** | | **1-5years** | | **5-10years** | | **＞10years** | | **Total** | |
| **Causes of death** | **No.observed** | **SMR(95% CI)** | **No.observed** | **SMR(95% CI)** | **No.observed** | **SMR(95% CI)** | **No.observed** | **SMR(95% CI)** | **No.observed** | **SMR(95% CI)** |
| **All Causes of Death** | 1,680 | 3.85(3.67-4.04)* | 5,726 | 3.30(3.21-3.38)* | 2,644 | 1.53(1.48-1.59)* | 1,800 | 1.33(1.27-1.4)* | 11,850 | 2.26(2.22-2.3)* |
| **Endometrial Cancer deaths** | 1,274 | 260.48(246.38-275.19)* | 3,728 | 196.91(190.64-203.33)* | 784 | 47.06(43.82-50.47)* | 137 | 12.37(10.38-14.62)* | 5,923 | 114.87(111.97-117.84)* |
| **Other cancer causes of deaths** | 147 | 1.34(1.14-1.58)* | 727 | 1.75(1.62-1.88)* | 503 | 1.36(1.25-1.49)* | 299 | 1.20(1.07-1.34)* | 1,676 | 1.47(1.4-1.54)* |
| **Non-cancer causes of deaths** | 259 | 0.80(0.71-0.91)* | 1,271 | 0.98(0.92-1.03) | 1,357 | 1.01(0.96-1.07) | 1,364 | 1.25(1.19-1.32)* | 4,251 | 1.05(1.02-1.08)* |
| **In situ, benign or unknown behavior neoplasm** | 4 | 1.57(0.43-4.02) | 18 | 1.77(1.05-2.79)* | 13 | 1.28(0.68-2.19) | 14 | 1.79(0.98-3.01) | 49 | 1.60(1.18-2.11)* |
| **Septicemia** | 11 | 1.55(0.77-2.77) | 44 | 1.57(1.14-2.1)* | 39 | 1.45(1.03-1.98)* | 25 | 1.25(0.81-1.85) | 119 | 1.45(1.2-1.73)* |
| **Other Infectious and Parasitic Diseases including HIV** | 3 | 0.82(0.17-2.39) | 17 | 1.17(0.68-1.88) | 24 | 1.78(1.14-2.65)* | 14 | 1.54(0.84-2.59) | 58 | 1.42(1.08-1.84)* |
| **Diabetes Mellitus** | 20 | 1.38(0.84-2.13) | 73 | 1.34(1.05-1.69)* | 76 | 1.56(1.23-1.95)* | 64 | 1.86(1.43-2.38)* | 233 | 1.53(1.34-1.74)* |
| **Alzheimers (ICD-9 and 10 only)** | 4 | 0.26(0.07-0.66)* | 33 | 0.45(0.31-0.63)* | 77 | 0.82(0.65-1.03) | 102 | 1.08(0.88-1.32) | 216 | 0.78(0.68-0.89)* |
| **Diseases of Heart** | 99 | 0.94(0.76-1.14) | 377 | 0.92(0.83-1.02) | 400 | 0.99(0.9-1.1) | 378 | 1.18(1.07-1.31)* | 1,254 | 1.01(0.96-1.07) |
| **Hypertension without Heart Disease** | 3 | 0.6(0.12-1.75) | 25 | 1.17(0.76-1.73) | 29 | 1.24(0.83-1.78) | 33 | 1.64(1.13-2.31)* | 90 | 1.29(1.04-1.58)* |
| **Cerebrovascular Diseases** | 20 | 0.7(0.43-1.08) | 91 | 0.82(0.66-1.01) | 105 | 0.95(0.77-1.15) | 107 | 1.17(0.96-1.42) | 323 | 0.95(0.85-1.05) |
| **Atherosclerosis** | 2 | 1.16(0.14-4.17) | 6 | 0.98(0.36-2.13) | 4 | 0.75(0.21-1.93) | 5 | 1.34(0.43-3.12) | 17 | 1.01(0.59-1.61) |
| **Aortic Aneurysm and Dissection** | 1 | 0.52(0.01-2.92) | 4 | 0.57(0.15-1.45) | 3 | 0.48(0.1-1.41) | 5 | 1.13(0.37-2.64) | 13 | 0.66(0.35-1.13) |
| **Other Diseases of Arteries, Arterioles, Capillaries** | 2 | 1.1(0.13-3.96) | 8 | 1.13(0.49-2.22) | 3 | 0.43(0.09-1.27) | 7 | 1.3(0.52-2.67) | 20 | 0.94(0.58-1.46) |
| **Pneumonia and Influenza** | 6 | 0.61(0.22-1.32) | 30 | 0.77(0.52-1.1) | 37 | 0.96(0.68-1.32) | 35 | 1.16(0.81-1.61) | 108 | 0.92(0.75-1.11) |
| **Chronic Obstructive Pulmonary Disease and Allied Cond** | 12 | 0.41(0.21-0.72)* | 62 | 0.52(0.4-0.67)* | 65 | 0.54(0.42-0.69)* | 59 | 0.65(0.49-0.83)* | 198 | 0.55(0.48-0.63)* |
| **Stomach and Duodenal Ulcers** | 1 | 1.62(0.04-9.03) | 3 | 1.3(0.27-3.8) | 3 | 1.41(0.29-4.12) | 5 | 3.10(1.01-7.22)* | 12 | 1.8(0.93-3.15) |
| **Chronic Liver Disease and Cirrhosis** | 4 | 0.95(0.26-2.43) | 11 | 0.68(0.34-1.21) | 8 | 0.58(0.25-1.14) | 13 | 1.46(0.78-2.49) | 36 | 0.83(0.58-1.15) |
| **Nephritis, Nephrotic Syndrome and Nephrosis** | 7 | 0.83(0.33-1.71) | 56 | 1.66(1.25-2.15)* | 52 | 1.55(1.16-2.03)* | 41 | 1.63(1.17-2.21)* | 156 | 1.54(1.31-1.81)* |
| **Complications of Pregnancy, Childbirth, Puerperium** | 0 | 0(0-179.36) | 1 | 15.49(0.39-86.3) | 1 | 27.21(0.69-151.62) | 0 | 0(0-293.13) | 2 | 14.87(1.8-53.73)* |
| **Congenital Anomalies** | 1 | 1.91(0.05-10.63) | 0 | 0(0-1.91) | 1 | 0.64(0.02-3.55) | 5 | 4.99(1.62-11.63)* | 7 | 1.39(0.56-2.87) |
| **Certain Conditions Originating in Perinatal Period** | 0 | 0(0-2,740.16) | 0 | 0(0-728.34) | 0 | 0(0-814.71) | 0 | 0(0-1,268.91) | 0 | 0(0-266.42) |
| **Symptoms, Signs and Ill-Defined Conditions** | 0 | 0.00(0-0.75)* | 26 | 1.26(0.82-1.84) | 18 | 0.79(0.47-1.24) | 13 | 0.76(0.4-1.29) | 57 | 0.87(0.66-1.12) |
| **Accidents and Adverse Effects** | 6 | 0.58(0.21-1.26) | 31 | 0.73(0.5-1.04) | 28 | 0.66(0.44-0.95)* | 40 | 1.16(0.83-1.58) | 105 | 0.81(0.66-0.98)* |
| **Suicide and Self-Inflicted Injury** | 0 | 0(0-2.38) | 2 | 0.35(0.04-1.26) | 2 | 0.46(0.06-1.65) | 4 | 1.64(0.45-4.2) | 8 | 0.57(0.24-1.12) |
| **Homicide and Legal Intervention** | 0 | 0(0-8.64) | 1 | 0.68(0.02-3.8) | 1 | 0.91(0.02-5.05) | 1 | 1.58(0.04-8.83) | 3 | 0.83(0.17-2.42) |
| **Other Cause of Death** | 40 | 0.63(0.45-0.85)* | 283 | 1.03(0.91-1.15) | 319 | 1.03(0.92-1.15) | 342 | 1.28(1.15-1.43)* | 984 | 1.07(1.01-1.14)* |

Abbreviation: SMR, standardized mortality ratio. CI: confidence interval

Values indicate the number of patients with cancer who died from each cause of death.

*P value less than .05

/

**Supporting Table 21.** Standardized-mortality ratios (SMRs) for each cause of death following Endometrial Cancer diagnosis in patients who received chemotherapy.

| **Timing of deaths after diagnosis** | | | | | | | | | | |
| --- | --- | --- | --- | --- | --- | --- | --- | --- | --- | --- |
|  | **＜1year** | | **1-5years** | | **5-10years** | | **＞10years** | | **Total** | |
| **Causes of death** | **No.observed** | **SMR(95% CI)** | **No.observed** | **SMR(95% CI)** | **No.observed** | **SMR(95% CI)** | **No.observed** | **SMR(95% CI)** | **No.observed** | **SMR(95% CI)** |
| **All Causes of Death** | 2,092 | 10.97(10.51-11.45)* | 5,134 | 8.15(7.39-8.38)* | 1,133 | 2.35(2.22-2.49)* | 362 | 1.50(1.35-1.66)* | 8,721 | 5.65(5.53-5.77)* |
| **Endometrial Cancer deaths** | 1,705 | 631.80(602.17-662.52)* | 3,891 | 446.18(432.27-460.42)* | 572 | 98.21(90.33-106.6)* | 52 | 21.37(15.96-28.03)* | 6,220 | 316.12(308.31-324.07)* |
| **Other cancer causes of deaths** | 236 | 4.43(3.88-5.03)* | 741 | 4.41(4.10-4.74)* | 207 | 1.81(1.57-2.07)* | 78 | 1.57(1.24-1.96)* | 1,262 | 3.27(3.1-3.46)* |
| **Non-cancer causes of deaths** | 151 | 1.12(0.95-1.31) | 502 | 1.11(1.01-1.21)* | 354 | 0.98(0.88-1.09) | 232 | 1.23(1.07-1.39)* | 1,239 | 1.09(1.03-1.15)* |
| **In situ, benign or unknown behavior neoplasm** | 4 | 3.61(0.98-9.24) | 15 | 4.09(2.29-6.74)* | 4 | 1.42(0.39-3.65) | 1 | 0.72(0.02-4.03) | 24 | 2.68(1.71-3.98)* |
| **Septicemia** | 14 | 4.22(2.31-7.08)* | 23 | 2.12(1.34-3.18)* | 15 | 1.89(1.06-3.12)* | 5 | 1.35(0.44-3.15) | 57 | 2.21(1.67-2.86)* |
| **Other Infectious and Parasitic Diseases including HIV** | 2 | 1.06(0.13-3.82) | 8 | 1.36(0.59-2.68) | 4 | 1.01(0.28-2.59) | 3 | 1.75(0.36-5.11) | 17 | 1.26(0.74-2.02) |
| **Diabetes Mellitus** | 7 | 1.03(0.41-2.11) | 10 | 0.47(0.22-0.86)* | 16 | 1.07(0.61-1.74) | 10 | 1.48(0.71-2.73) | 43 | 0.86(0.62-1.16) |
| **Alzheimers (ICD-9 and 10 only)** | 1 | 0.18(0-1.01) | 7 | 0.31(0.13-0.64)* | 13 | 0.56(0.3-0.96)* | 7 | 0.46(0.19-0.96)* | 28 | 0.42(0.28-0.61)* |
| **Diseases of Heart** | 41 | 0.99(0.71-1.35) | 113 | 0.85(0.70-1.02) | 109 | 1.05(0.86-1.26) | 56 | 1.03(0.77-1.33) | 319 | 0.96(0.85-1.07) |
| **Hypertension without Heart Disease** | 0 | 0(0-1.69) | 7 | 0.92(0.37-1.89) | 11 | 1.72(0.86-3.07) | 10 | 2.86(1.37-5.25)* | 28 | 1.42(0.94-2.05) |
| **Cerebrovascular Diseases** | 11 | 1(0.5-1.79) | 39 | 1.08(0.77-1.47) | 23 | 0.79(0.5-1.19) | 23 | 1.47(0.93-2.21) | 96 | 1.04(0.85-1.27) |
| **Atherosclerosis** | 0 | 0(0-7.11) | 2 | 1.32(0.16-4.76) | 0 | 0(0-3.3) | 0 | 0(0-6.43) | 2 | 0.54(0.06-1.94) |
| **Aortic Aneurysm and Dissection** | 0 | 0(0-4.83) | 2 | 0.85(0.10-3.08) | 2 | 1.19(0.14-4.31) | 0 | 0(0-4.66) | 4 | 0.72(0.2-1.84) |
| **Other Diseases of Arteries, Arterioles, Capillaries** | 1 | 1.35(0.03-7.52) | 3 | 1.24(0.26-3.62) | 1 | 0.54(0.01-2.99) | 2 | 2.11(0.26-7.61) | 7 | 1.17(0.47-2.41) |
| **Pneumonia and Influenza** | 5 | 1.34(0.44-3.14) | 15 | 1.21(0.68-1.99) | 10 | 1.01(0.49-1.86) | 6 | 1.17(0.43-2.54) | 36 | 1.16(0.81-1.6) |
| **Chronic Obstructive Pulmonary Disease and Allied Cond** | 4 | 0.30(0.08-0.78)* | 18 | 0.40(0.24-0.63)* | 17 | 0.49(0.29-0.79)* | 8 | 0.48(0.21-0.95)* | 47 | 0.43(0.32-0.57)* |
| **Stomach and Duodenal Ulcers** | 0 | 0(0-15) | 0 | 0(0-4.66) | 3 | 5.05(1.04-14.75)* | 2 | 6.75(0.82-24.39) | 5 | 2.59(0.84-6.05) |
| **Chronic Liver Disease and Cirrhosis** | 0 | 0(0-1.6) | 3 | 0.4(0.08-1.17) | 5 | 0.99(0.32-2.32) | 1 | 0.48(0.01-2.68) | 9 | 0.53(0.24-1.01) |
| **Nephritis, Nephrotic Syndrome and Nephrosis** | 3 | 0.78(0.16-2.27) | 19 | 1.5(0.90-2.34) | 13 | 1.37(0.73-2.34) | 9 | 1.96(0.9-3.72) | 44 | 1.44(1.04-1.93)* |
| **Complications of Pregnancy, Childbirth, Puerperium** | 1 | 63.88(1.62-355.91)* | 2 | 49.20(5.96-177.74)* | 0 | 0(0-211.41) | 0 | 0(0-821.12) | 3 | 38.34(7.91-112.05)* |
| **Congenital Anomalies** | 0 | 0(0-13.79) | 0 | 0(0-4.38) | 0 | 0(0-6.8) | 0 | 0(0-16.36) | 0 | 0(0-1.96) |
| **Certain Conditions Originating in Perinatal Period** | 0 | 0(0-4,650.18) | 0 | 0(0-1,392.33) | 0 | 0(0-2,071.61) | 0 | 0(0-5,025.44) | 0 | 0(0-619.21) |
| **Symptoms, Signs and Ill-Defined Conditions** | 4 | 2.04(0.56-5.23) | 12 | 1.85(0.96-3.24) | 5 | 0.95(0.31-2.23) | 2 | 0.73(0.09-2.63) | 23 | 1.4(0.89-2.1) |
| **Accidents and Adverse Effects** | 7 | 1.41(0.57-2.91) | 12 | 0.71(0.37-1.25) | 8 | 0.63(0.27-1.24) | 9 | 1.41(0.65-2.68) | 36 | 0.88(0.62-1.22) |
| **Suicide and Self-Inflicted Injury** | 0 | 0(0-4.07) | 2 | 0.7(0.09-2.54) | 1 | 0.58(0.01-3.22) | 1 | 1.54(0.04-8.59) | 4 | 0.65(0.18-1.67) |
| **Homicide and Legal Intervention** | 0 | 0(0-15.26) | 1 | 1.43(0.04-7.94) | 0 | 0(0-9.04) | 0 | 0(0-24.05) | 1 | 0.66(0.02-3.7) |
| **Other Cause of Death** | 31 | 1.12(0.76-1.58) | 117 | 1.18(0.98-1.42) | 74 | 0.88(0.69-1.11) | 66 | 1.44(1.11-1.83)* | 288 | 1.12(1-1.26) |

Abbreviation: SMR, standardized mortality ratio. CI: confidence interval

Values indicate the number of patients with cancer who died from each cause of death.

*P value less than .05

**Supporting Table 22.** Standardized-mortality ratios (SMRs) for each cause of death following Endometrial Cancer diagnosis in patients who received surgery.

| **Timing of deaths after diagnosis** | | | | | | | | | | |
| --- | --- | --- | --- | --- | --- | --- | --- | --- | --- | --- |
|  | **＜1year** | | **1-5years** | | **5-10years** | | **＞10years** | | **Total** | |
| **Causes of death** | **No.observed** | **SMR(95% CI)** | **No.observed** | **SMR(95% CI)** | **No.observed** | **SMR(95% CI)** | **No.observed** | **SMR(95% CI)** | **No.observed** | **SMR(95% CI)** |
| **All Causes of Death** | 4,535 | 3.21(3.12-3.31)* | 13,455 | 2.22(2.18-2.26)* | 7,948 | 1.22(1.2-1.25)* | 6,257 | 1.15(1.12-1.18)* | 32,195 | 1.66(1.64-1.68)* |
| **Endometrial Cancer deaths** | 3,075 | 192.75(186-199.69)* | 7,555 | 113.61(111.06-116.2)* | 1,613 | 25.06(23.85-26.31)* | 313 | 6.75(6.03-7.54)* | 12,556 | 65.00(63.86-66.14)* |
| **Other cancer causes of deaths** | 492 | 1.39(1.27-1.52)* | 2,197 | 1.52(1.45-1.58)* | 1,570 | 1.12(1.06-1.17)* | 1,036 | 1.01(0.95-1.07) | 5,295 | 1.25(1.22-1.28)* |
| **Non-cancer causes of deaths** | 968 | 0.93(0.87-0.99)* | 3,703 | 0.81(0.79-0.84)* | 4,765 | 0.95(0.92-0.97)* | 4,908 | 1.12(1.09-1.15)* | 14,344 | 0.96(0.94-0.97)* |
| **In situ, benign or unknown behavior neoplasm** | 13 | 1.58(0.84-2.71) | 51 | 1.44(1.08-1.9)* | 44 | 1.16(0.84-1.55) | 39 | 1.24(0.88-1.69) | 147 | 1.30(1.1-1.53)* |
| **Septicemia** | 47 | 2.08(1.53-2.77)* | 99 | 1.03(0.83-1.25) | 120 | 1.19(0.99-1.42) | 98 | 1.22(0.99-1.48) | 364 | 1.21(1.09-1.34)* |
| **Other Infectious and Parasitic Diseases including HIV** | 14 | 1.16(0.63-1.94) | 50 | 0.97(0.72-1.28) | 63 | 1.22(0.94-1.56) | 48 | 1.29(0.95-1.71) | 175 | 1.15(0.98-1.33) |
| **Diabetes Mellitus** | 53 | 1.16(0.87-1.51) | 225 | 1.20(1.05-1.37)* | 254 | 1.39(1.22-1.57)* | 227 | 1.62(1.42-1.85)* | 759 | 1.37(1.27-1.47)* |
| **Alzheimers (ICD-9 and 10 only)** | 18 | 0.36(0.21-0.57)* | 107 | 0.42(0.34-0.5)* | 279 | 0.80(0.71-0.9)* | 417 | 1.13(1.02-1.24)* | 821 | 0.80(0.75-0.86)* |
| **Diseases of Heart** | 324 | 0.95(0.85-1.06) | 1,131 | 0.79(0.74-0.84)* | 1,498 | 0.99(0.94-1.04) | 1,400 | 1.09(1.04-1.15)* | 4,353 | 0.95(0.92-0.98)* |
| **Hypertension without Heart Disease** | 10 | 0.63(0.3-1.16) | 80 | 1.09(0.86-1.35) | 106 | 1.22(1-1.47) | 123 | 1.54(1.28-1.83)* | 319 | 1.24(1.11-1.39)* |
| **Cerebrovascular Diseases** | 85 | 0.93(0.74-1.15) | 315 | 0.82(0.73-0.91)* | 369 | 0.89(0.8-0.99)* | 403 | 1.11(1-1.22)* | 1,172 | 0.93(0.88-0.99)* |
| **Atherosclerosis** | 4 | 0.7(0.19-1.79) | 24 | 1.1(0.7-1.63) | 18 | 0.89(0.53-1.41) | 23 | 1.54(0.97-2.3) | 69 | 1.1(0.86-1.39) |
| **Aortic Aneurysm and Dissection** | 0 | 0.00(0-0.6)* | 13 | 0.54(0.28-0.91)* | 10 | 0.43(0.21-0.79)* | 12 | 0.67(0.35-1.18) | 35 | 0.49(0.34-0.68)* |
| **Other Diseases of Arteries, Arterioles, Capillaries** | 6 | 1.03(0.38-2.25) | 20 | 0.81(0.5-1.26) | 17 | 0.66(0.38-1.05) | 17 | 0.79(0.46-1.26) | 60 | 0.77(0.59-0.99)* |
| **Pneumonia and Influenza** | 20 | 0.62(0.38-0.96)* | 108 | 0.78(0.64-0.95)* | 146 | 1(0.85-1.18) | 131 | 1.08(0.9-1.28) | 405 | 0.93(0.84-1.02) |
| **Chronic Obstructive Pulmonary Disease and Allied Cond** | 42 | 0.45(0.33-0.61)* | 156 | 0.38(0.32-0.45)* | 237 | 0.53(0.47-0.6)* | 251 | 0.68(0.6-0.77)* | 686 | 0.52(0.48-0.56)* |
| **Stomach and Duodenal Ulcers** | 4 | 1.98(0.54-5.06) | 13 | 1.6(0.85-2.73) | 11 | 1.36(0.68-2.44) | 7 | 1.07(0.43-2.2) | 35 | 1.41(0.98-1.96) |
| **Chronic Liver Disease and Cirrhosis** | 13 | 0.9(0.48-1.54) | 55 | 0.92(0.69-1.19) | 55 | 0.98(0.74-1.28) | 36 | 0.93(0.65-1.29) | 159 | 0.94(0.8-1.1) |
| **Nephritis, Nephrotic Syndrome and Nephrosis** | 34 | 1.28(0.89-1.79) | 133 | 1.15(0.97-1.37) | 149 | 1.20(1.01-1.41)* | 145 | 1.44(1.21-1.69)* | 461 | 1.26(1.14-1.38)* |
| **Complications of Pregnancy, Childbirth, Puerperium** | 1 | 9.66(0.24-53.82) | 2 | 5.66(0.69-20.44) | 1 | 4.65(0.12-25.93) | 0 | 0(0-50.28) | 4 | 5.37(1.46-13.74)* |
| **Congenital Anomalies** | 2 | 1.09(0.13-3.93) | 4 | 0.55(0.15-1.41) | 6 | 0.93(0.34-2.03) | 6 | 1.38(0.51-3.01) | 18 | 0.91(0.54-1.43) |
| **Certain Conditions Originating in Perinatal Period** | 0 | 0(0-720.55) | 0 | 0(0-178.99) | 0 | 0(0-189.34) | 0 | 0(0-280.77) | 0 | 0(0-63.22) |
| **Symptoms, Signs and Ill-Defined Conditions** | 18 | 1.1(0.65-1.75) | 66 | 0.89(0.69-1.14) | 84 | 0.96(0.77-1.19) | 53 | 0.77(0.58-1) | 221 | 0.9(0.78-1.02) |
| **Accidents and Adverse Effects** | 24 | 0.68(0.43-1) | 107 | 0.69(0.56-0.83)* | 120 | 0.72(0.6-0.86)* | 119 | 0.84(0.7-1.01) | 370 | 0.74(0.67-0.82)* |
| **Suicide and Self-Inflicted Injury** | 3 | 0.51(0.1-1.48) | 10 | 0.43(0.21-0.79)* | 17 | 0.88(0.51-1.4) | 13 | 1.15(0.61-1.96) | 43 | 0.72(0.52-0.97)* |
| **Homicide and Legal Intervention** | 0 | 0(0-2.35) | 4 | 0.7(0.19-1.78) | 3 | 0.65(0.13-1.89) | 3 | 1.08(0.22-3.16) | 10 | 0.68(0.33-1.25) |
| **Other Cause of Death** | 191 | 0.92(0.8-1.07) | 742 | 0.77(0.72-0.83)* | 1,004 | 0.87(0.81-0.92)* | 1,155 | 1.08(1.02-1.15)* | 3,092 | 0.91(0.88-0.94)* |

Abbreviation: SMR, standardized mortality ratio. CI: confidence interval

Values indicate the number of patients with cancer who died from each cause of death.

*P value less than .05

**Supporting Table 23.** Standardized-mortality ratios (SMRs) for each cause of death following Endometrial Cancer diagnosis in patients who was single.

| **Timing of deaths after diagnosis** | | | | | | | | | | |
| --- | --- | --- | --- | --- | --- | --- | --- | --- | --- | --- |
|  | **＜1year** | | **1-5years** | | **5-10years** | | **＞10years** | | **Total** | |
| **Causes of death** | **No.observed** | **SMR(95% CI)** | **No.observed** | **SMR(95% CI)** | **No.observed** | **SMR(95% CI)** | **No.observed** | **SMR(95% CI)** | **No.observed** | **SMR(95% CI)** |
| **All Causes of Death** | 1,271 | 7.26(6.87-7.67)* | 2,614 | 3.59(3.45-3.73)* | 1,333 | 1.79(1.7-1.89)* | 893 | 1.57(1.47-1.68)* | 6,111 | 2.76(2.69-2.83)* |
| **Endometrial Cancer deaths** | 913 | 363.73(340.51-388.11)* | 1,526 | 146.85(139.57-154.4)* | 307 | 31.05(27.67-34.72)* | 50 | 7.40(5.49-9.75)* | 2,796 | 94.62(91.14-98.19)* |
| **Other cancer causes of deaths** | 121 | 2.52(2.09-3.02)* | 371 | 1.91(1.72-2.11)* | 256 | 1.39(1.22-1.57)* | 160 | 1.25(1.07-1.46)* | 908 | 1.64(1.53-1.75)* |
| **Non-cancer causes of deaths** | 237 | 1.90(1.67-2.16)* | 717 | 1.37(1.27-1.47)* | 770 | 1.40(1.31-1.51)* | 683 | 1.57(1.46-1.69)* | 2,407 | 1.48(1.42-1.54)* |
| **In situ, benign or unknown behavior neoplasm** | 2 | 2.04(0.25-7.37) | 9 | 2.20(1.01-4.18)* | 5 | 1.19(0.39-2.77) | 2 | 0.62(0.08-2.24) | 18 | 1.44(0.85-2.28) |
| **Septicemia** | 5 | 1.69(0.55-3.95) | 22 | 1.79(1.12-2.71)* | 28 | 2.27(1.51-3.28)* | 16 | 1.75(1-2.85)* | 71 | 1.93(1.51-2.44)* |
| **Other Infectious and Parasitic Diseases including HIV** | 4 | 2.09(0.57-5.35) | 12 | 1.58(0.82-2.77) | 14 | 2.02(1.1-3.39)* | 6 | 1.32(0.48-2.88) | 36 | 1.72(1.2-2.38)* |
| **Diabetes Mellitus** | 23 | 3.76(2.39-5.65)* | 45 | 1.84(1.34-2.46)* | 61 | 2.60(1.99-3.34)* | 48 | 2.85(2.1-3.77)* | 177 | 2.49(2.14-2.89)* |
| **Alzheimers (ICD-9 and 10 only)** | 0 | 0.00(0-0.76)* | 14 | 0.59(0.32-1)* | 26 | 0.85(0.55-1.24) | 28 | 0.93(0.62-1.34) | 68 | 0.76(0.59-0.97)* |
| **Diseases of Heart** | 76 | 1.94(1.53-2.43)* | 222 | 1.40(1.22-1.6)* | 243 | 1.51(1.33-1.72)* | 204 | 1.64(1.42-1.88)* | 745 | 1.54(1.44-1.66)* |
| **Hypertension without Heart Disease** | 4 | 2.08(0.57-5.32) | 13 | 1.54(0.82-2.63) | 17 | 1.81(1.05-2.9)* | 14 | 1.78(0.97-2.99) | 48 | 1.74(1.28-2.3)* |
| **Cerebrovascular Diseases** | 14 | 1.36(0.74-2.28) | 36 | 0.86(0.6-1.19) | 41 | 0.95(0.68-1.29) | 43 | 1.24(0.9-1.67) | 134 | 1.03(0.87-1.22) |
| **Atherosclerosis** | 1 | 1.78(0.04-9.89) | 4 | 1.95(0.53-5) | 3 | 1.63(0.34-4.78) | 4 | 3.06(0.84-7.85) | 12 | 2.09(1.08-3.65)* |
| **Aortic Aneurysm and Dissection** | 0 | 0(0-5.39) | 0 | 0(0-1.37) | 1 | 0.39(0.01-2.19) | 2 | 1.08(0.13-3.9) | 3 | 0.39(0.08-1.13) |
| **Other Diseases of Arteries, Arterioles, Capillaries** | 2 | 2.94(0.36-10.63) | 0 | 0(0-1.32) | 3 | 1.06(0.22-3.09) | 5 | 2.27(0.74-5.3) | 10 | 1.18(0.56-2.16) |
| **Pneumonia and Influenza** | 5 | 1.4(0.45-3.26) | 15 | 1.02(0.57-1.68) | 23 | 1.53(0.97-2.29) | 22 | 1.87(1.17-2.82)* | 65 | 1.44(1.11-1.83)* |
| **Chronic Obstructive Pulmonary Disease and Allied Cond** | 10 | 0.92(0.44-1.7) | 27 | 0.57(0.38-0.83)* | 36 | 0.71(0.5-0.99)* | 36 | 0.91(0.64-1.26) | 109 | 0.74(0.6-0.89)* |
| **Stomach and Duodenal Ulcers** | 0 | 0(0-15.53) | 4 | 4.24(1.15-10.85)* | 2 | 2.18(0.26-7.87) | 0 | 0(0-5.3) | 6 | 2.15(0.79-4.67) |
| **Chronic Liver Disease and Cirrhosis** | 5 | 2.13(0.69-4.96) | 12 | 1.22(0.63-2.13) | 9 | 0.99(0.45-1.87) | 11 | 1.82(0.91-3.26) | 37 | 1.35(0.95-1.87) |
| **Nephritis, Nephrotic Syndrome and Nephrosis** | 7 | 2.09(0.84-4.3) | 35 | 2.50(1.74-3.48)* | 25 | 1.73(1.12-2.56)* | 23 | 2.11(1.34-3.17)* | 90 | 2.11(1.7-2.59)* |
| **Complications of Pregnancy, Childbirth, Puerperium** | 0 | 0(0-91.41) | 1 | 7.18(0.18-39.99) | 1 | 11.2(0.28-62.41) | 0 | 0(0-108.07) | 2 | 6.6(0.8-23.84) |
| **Congenital Anomalies** | 0 | 0(0-12) | 1 | 0.82(0.02-4.58) | 3 | 2.86(0.59-8.34) | 1 | 1.51(0.04-8.41) | 5 | 1.55(0.5-3.61) |
| **Certain Conditions Originating in Perinatal Period** | 0 | 0(0-3,349.11) | 0 | 0(0-825.59) | 0 | 0(0-936.09) | 0 | 0(0-1,471.28) | 0 | 0(0-306.96) |
| **Symptoms, Signs and Ill-Defined Conditions** | 10 | 5.18(2.48-9.52)* | 6 | 0.75(0.27-1.62) | 17 | 1.95(1.14-3.12)* | 3 | 0.47(0.1-1.37) | 36 | 1.43(1-1.98)* |
| **Accidents and Adverse Effects** | 5 | 0.92(0.3-2.16) | 28 | 1.24(0.82-1.79) | 29 | 1.32(0.89-1.9) | 17 | 1.06(0.62-1.7) | 79 | 1.2(0.95-1.49) |
| **Suicide and Self-Inflicted Injury** | 1 | 0.84(0.02-4.68) | 1 | 0.21(0.01-1.19) | 4 | 1.04(0.28-2.67) | 4 | 1.85(0.5-4.74) | 10 | 0.84(0.4-1.55) |
| **Homicide and Legal Intervention** | 0 | 0(0-10.64) | 0 | 0(0-2.98) | 2 | 2.13(0.26-7.69) | 0 | 0(0-7.14) | 2 | 0.66(0.08-2.38) |
| **Other Cause of Death** | 51 | 2.06(1.53-2.7)* | 154 | 1.40(1.19-1.64)* | 152 | 1.23(1.04-1.44)* | 164 | 1.59(1.35-1.85)* | 521 | 1.44(1.32-1.57)* |

Abbreviation: SMR, standardized mortality ratio. CI: confidence interval

Values indicate the number of patients with cancer who died from each cause of death.

*P value less than .05

**Supporting Table 24.** Standardized-mortality ratios (SMRs) for each cause of death following Endometrial Cancer diagnosis in patients who was married.

| **Timing of deaths after diagnosis** | | | | | | | | | | |
| --- | --- | --- | --- | --- | --- | --- | --- | --- | --- | --- |
|  | **＜1year** | | **1-5years** | | **5-10years** | | **＞10years** | | **Total** | |
| **Causes of death** | **No.observed** | **SMR(95% CI)** | **No.observed** | **SMR(95% CI)** | **No.observed** | **SMR(95% CI)** | **No.observed** | **SMR(95% CI)** | **No.observed** | **SMR(95% CI)** |
| **All Causes of Death** | 2,417 | 3.89(3.73-4.05)* | 6,332 | 2.25(2.19-2.31)* | 3,697 | 1.12(1.09-1.16)* | 3,125 | 0.99(0.95-1.02) | 15,571 | 1.57(1.55-1.6)* |
| **Endometrial Cancer deaths** | 1,727 | 209.08(199.33-219.18)* | 3,763 | 105.23(101.89-108.64)* | 921 | 25.16(23.56-26.84)* | 196 | 6.89(5.96-7.93)* | 6,607 | 60.58(59.13-62.06)* |
| **Other cancer causes of deaths** | 261 | 1.46(1.29-1.65)* | 1,069 | 1.40(1.32-1.49)* | 772 | 0.98(0.91-1.05) | 563 | 0.90(0.82-0.97)* | 2,665 | 1.13(1.09-1.17)* |
| **Non-cancer causes of deaths** | 429 | 0.99(0.9-1.09) | 1,500 | 0.74(0.71-0.78)* | 2,004 | 0.81(0.78-0.85)* | 2,366 | 0.94(0.91-0.98)* | 6,299 | 0.85(0.83-0.87)* |
| **In situ, benign or unknown behavior neoplasm** | 10 | 2.75(1.32-5.05)* | 21 | 1.27(0.79-1.94) | 19 | 0.97(0.59-1.52) | 23 | 1.24(0.79-1.87) | 73 | 1.25(0.98-1.58) |
| **Septicemia** | 22 | 2.19(1.37-3.31)* | 42 | 0.92(0.66-1.24) | 51 | 0.98(0.73-1.28) | 48 | 1.01(0.75-1.34) | 163 | 1.05(0.89-1.22) |
| **Other Infectious and Parasitic Diseases including HIV** | 8 | 1.37(0.59-2.7) | 25 | 0.97(0.63-1.44) | 29 | 1.06(0.71-1.52) | 22 | 1(0.63-1.51) | 84 | 1.04(0.83-1.28) |
| **Diabetes Mellitus** | 30 | 1.41(0.95-2.02) | 109 | 1.19(0.98-1.44) | 93 | 0.96(0.78-1.18) | 103 | 1.24(1.02-1.51)* | 335 | 1.15(1.03-1.28)* |
| **Alzheimers (ICD-9 and 10 only)** | 7 | 0.42(0.17-0.86)* | 40 | 0.42(0.3-0.57)* | 124 | 0.80(0.67-0.96)* | 226 | 1.1(0.96-1.25) | 397 | 0.84(0.76-0.93)* |
| **Diseases of Heart** | 133 | 0.97(0.81-1.15) | 437 | 0.71(0.65-0.78)* | 594 | 0.82(0.75-0.88)* | 652 | 0.90(0.83-0.97)* | 1,816 | 0.82(0.79-0.86)* |
| **Hypertension without Heart Disease** | 6 | 0.98(0.36-2.13) | 25 | 0.82(0.53-1.21) | 40 | 0.98(0.7-1.33) | 52 | 1.16(0.86-1.52) | 123 | 1(0.83-1.2) |
| **Cerebrovascular Diseases** | 35 | 0.97(0.67-1.34) | 133 | 0.81(0.68-0.96)* | 179 | 0.9(0.77-1.04) | 206 | 1(0.87-1.14) | 553 | 0.91(0.84-0.99)* |
| **Atherosclerosis** | 1 | 0.5(0.01-2.8) | 8 | 0.96(0.41-1.88) | 7 | 0.77(0.31-1.59) | 12 | 1.45(0.75-2.53) | 28 | 1.01(0.67-1.46) |
| **Aortic Aneurysm and Dissection** | 0 | 0(0-1.37) | 7 | 0.61(0.25-1.26) | 7 | 0.58(0.23-1.2) | 6 | 0.57(0.21-1.24) | 20 | 0.55(0.33-0.84)* |
| **Other Diseases of Arteries, Arterioles, Capillaries** | 3 | 1.24(0.26-3.63) | 9 | 0.83(0.38-1.57) | 7 | 0.55(0.22-1.14) | 6 | 0.48(0.18-1.05) | 25 | 0.65(0.42-0.96)* |
| **Pneumonia and Influenza** | 7 | 0.56(0.23-1.16) | 44 | 0.76(0.55-1.02) | 61 | 0.87(0.67-1.12) | 47 | 0.68(0.5-0.9)* | 159 | 0.76(0.65-0.89)* |
| **Chronic Obstructive Pulmonary Disease and Allied Cond** | 14 | 0.32(0.18-0.54)* | 58 | 0.29(0.22-0.37)* | 94 | 0.39(0.32-0.48)* | 128 | 0.57(0.48-0.68)* | 294 | 0.41(0.37-0.46)* |
| **Stomach and Duodenal Ulcers** | 0 | 0(0-4.21) | 5 | 1.33(0.43-3.1) | 5 | 1.21(0.39-2.82) | 5 | 1.3(0.42-3.02) | 15 | 1.19(0.66-1.96) |
| **Chronic Liver Disease and Cirrhosis** | 11 | 1.37(0.68-2.44) | 23 | 0.67(0.42-1.01) | 32 | 0.96(0.65-1.35) | 19 | 0.78(0.47-1.21) | 85 | 0.85(0.68-1.05) |
| **Nephritis, Nephrotic Syndrome and Nephrosis** | 11 | 0.98(0.49-1.76) | 58 | 1.12(0.85-1.44) | 63 | 1.02(0.78-1.3) | 68 | 1.17(0.91-1.48) | 200 | 1.09(0.94-1.25) |
| **Complications of Pregnancy, Childbirth, Puerperium** | 1 | 17.73(0.45-98.76) | 1 | 5.17(0.13-28.83) | 0 | 0(0-31.6) | 0 | 0(0-97.72) | 2 | 4.95(0.6-17.87) |
| **Congenital Anomalies** | 2 | 2(0.24-7.24) | 3 | 0.74(0.15-2.17) | 1 | 0.27(0.01-1.51) | 2 | 0.76(0.09-2.73) | 8 | 0.7(0.3-1.39) |
| **Certain Conditions Originating in Perinatal Period** | 0 | 0(0-1,293.25) | 0 | 0(0-316.48) | 0 | 0(0-322.68) | 0 | 0(0-462.26) | 0 | 0(0-108.75) |
| **Symptoms, Signs and Ill-Defined Conditions** | 5 | 0.8(0.26-1.87) | 22 | 0.73(0.46-1.11) | 31 | 0.77(0.53-1.1) | 27 | 0.71(0.47-1.03) | 85 | 0.74(0.59-0.92)* |
| **Accidents and Adverse Effects** | 11 | 0.65(0.32-1.16) | 39 | 0.51(0.36-0.7)* | 52 | 0.60(0.45-0.79)* | 57 | 0.69(0.52-0.9)* | 159 | 0.61(0.52-0.71)* |
| **Suicide and Self-Inflicted Injury** | 1 | 0.29(0.01-1.59) | 2 | 0.14(0.02-0.52)* | 10 | 0.84(0.4-1.55) | 5 | 0.7(0.23-1.63) | 18 | 0.49(0.29-0.78)* |
| **Homicide and Legal Intervention** | 0 | 0(0-4.28) | 2 | 0.62(0.08-2.25) | 0 | 0(0-1.38) | 3 | 1.77(0.37-5.17) | 5 | 0.59(0.19-1.38) |
| **Other Cause of Death** | 93 | 1.09(0.88-1.33) | 306 | 0.72(0.64-0.81)* | 432 | 0.77(0.7-0.84)* | 559 | 0.92(0.84-1)* | 1,390 | 0.83(0.78-0.87)* |

Abbreviation: SMR, standardized mortality ratio. CI: confidence interval

Values indicate the number of patients with cancer who died from each cause of death.

*P value less than .05

**Supporting Table 25.** Standardized-mortality ratios (SMRs) for each cause of death following Endometrial Cancer diagnosis in patients who was SWD.

| **Timing of deaths after diagnosis** | | | | | | | | | | |
| --- | --- | --- | --- | --- | --- | --- | --- | --- | --- | --- |
|  | **＜1year** | | **1-5years** | | **5-10years** | | **＞10years** | | **Total** | |
| **Causes of death** | **No.observed** | **SMR(95% CI)** | **No.observed** | **SMR(95% CI)** | **No.observed** | **SMR(95% CI)** | **No.observed** | **SMR(95% CI)** | **No.observed** | **SMR(95% CI)** |
| **All Causes of Death** | 2,721 | 3.83(3.68-3.97)* | 5,810 | 2.15(2.09-2.2)* | 3,232 | 1.27(1.22-1.31)* | 2,345 | 1.32(1.27-1.38)* | 14,108 | 1.82(1.79-1.85)* |
| **Endometrial Cancer deaths** | 1,801 | 306.74(292.73-321.24)* | 2,990 | 137.32(132.44-142.33)* | 477 | 25.50(23.27-27.9)* | 79 | 6.73(5.33-8.39)* | 5,347 | 92.06(89.61-94.56)* |
| **Other cancer causes of deaths** | 287 | 2.00(1.77-2.24)* | 867 | 1.65(1.54-1.77)* | 572 | 1.27(1.17-1.38)* | 328 | 1.16(1.04-1.29)* | 2,054 | 1.47(1.4-1.53)* |
| **Non-cancer causes of deaths** | 633 | 1.13(1.04-1.22)* | 1,953 | 0.90(0.86-0.95)* | 2,183 | 1.05(1.01-1.09)* | 1,938 | 1.31(1.25-1.37)* | 6,707 | 1.07(1.04-1.09)* |
| **In situ, benign or unknown behavior neoplasm** | 5 | 1.21(0.39-2.83) | 24 | 1.53(0.98-2.27) | 20 | 1.35(0.83-2.09) | 16 | 1.58(0.91-2.57) | 65 | 1.45(1.12-1.85)* |
| **Septicemia** | 29 | 2.63(1.76-3.78)* | 54 | 1.31(0.98-1.7) | 51 | 1.36(1.01-1.78)* | 37 | 1.48(1.04-2.04)* | 171 | 1.49(1.27-1.73)* |
| **Other Infectious and Parasitic Diseases including HIV** | 7 | 1.4(0.56-2.88) | 18 | 0.92(0.54-1.45) | 25 | 1.39(0.9-2.04) | 21 | 1.89(1.17-2.9)* | 71 | 1.32(1.03-1.67)* |
| **Diabetes Mellitus** | 25 | 1.18(0.76-1.74) | 104 | 1.36(1.11-1.65)* | 108 | 1.65(1.36-2)* | 82 | 1.96(1.56-2.43)* | 319 | 1.56(1.39-1.74)* |
| **Alzheimers (ICD-9 and 10 only)** | 19 | 0.56(0.34-0.87)* | 73 | 0.49(0.39-0.62)* | 142 | 0.85(0.72-1) | 165 | 1.20(1.02-1.39)* | 399 | 0.82(0.74-0.9)* |
| **Diseases of Heart** | 243 | 1.26(1.11-1.43)* | 644 | 0.91(0.84-0.98)* | 719 | 1.11(1.03-1.19)* | 571 | 1.29(1.19-1.4)* | 2,177 | 1.09(1.05-1.14)* |
| **Hypertension without Heart Disease** | 7 | 0.76(0.31-1.58) | 46 | 1.24(0.91-1.65) | 52 | 1.37(1.02-1.79)* | 59 | 2.09(1.59-2.69)* | 164 | 1.46(1.24-1.7)* |
| **Cerebrovascular Diseases** | 64 | 1.21(0.94-1.55) | 172 | 0.89(0.76-1.03) | 162 | 0.91(0.78-1.06) | 160 | 1.27(1.08-1.48)* | 558 | 1.01(0.93-1.1) |
| **Atherosclerosis** | 4 | 1.08(0.29-2.75) | 15 | 1.21(0.68-2) | 8 | 0.84(0.36-1.66) | 7 | 1.26(0.51-2.59) | 34 | 1.09(0.76-1.52) |
| **Aortic Aneurysm and Dissection** | 2 | 0.64(0.08-2.32) | 9 | 0.83(0.38-1.57) | 2 | 0.22(0.03-0.81)* | 4 | 0.71(0.19-1.82) | 17 | 0.59(0.35-0.95)* |
| **Other Diseases of Arteries, Arterioles, Capillaries** | 5 | 1.58(0.51-3.68) | 14 | 1.19(0.65-2) | 8 | 0.75(0.32-1.48) | 6 | 0.83(0.3-1.8) | 33 | 1.01(0.69-1.41) |
| **Pneumonia and Influenza** | 16 | 0.84(0.48-1.37) | 61 | 0.87(0.66-1.12) | 68 | 1.08(0.84-1.37) | 63 | 1.50(1.15-1.92)* | 208 | 1.07(0.93-1.23) |
| **Chronic Obstructive Pulmonary Disease and Allied Cond** | 31 | 0.71(0.48-1.01) | 93 | 0.55(0.44-0.67)* | 125 | 0.78(0.65-0.93)* | 89 | 0.80(0.65-0.99)* | 338 | 0.70(0.63-0.78)* |
| **Stomach and Duodenal Ulcers** | 4 | 3.81(1.04-9.77)* | 4 | 1.09(0.3-2.79) | 4 | 1.28(0.35-3.29) | 3 | 1.44(0.3-4.21) | 15 | 1.51(0.85-2.5) |
| **Chronic Liver Disease and Cirrhosis** | 2 | 0.44(0.05-1.59) | 22 | 1.3(0.82-1.97) | 17 | 1.2(0.7-1.92) | 9 | 1.03(0.47-1.95) | 50 | 1.13(0.84-1.49) |
| **Nephritis, Nephrotic Syndrome and Nephrosis** | 24 | 1.73(1.11-2.57)* | 57 | 1.08(0.81-1.39) | 65 | 1.31(1.01-1.66)* | 55 | 1.67(1.26-2.17)* | 201 | 1.34(1.16-1.54)* |
| **Complications of Pregnancy, Childbirth, Puerperium** | 0 | 0(0-280.33) | 0 | 0(0-86.92) | 0 | 0(0-161.2) | 0 | 0(0-474.98) | 0 | 0(0-42.77) |
| **Congenital Anomalies** | 0 | 0(0-6.06) | 0 | 0(0-1.69) | 2 | 1.13(0.14-4.09) | 3 | 2.77(0.57-8.09) | 5 | 0.89(0.29-2.07) |
| **Certain Conditions Originating in Perinatal Period** | 0 | 0(0-2,704.39) | 0 | 0(0-735.4) | 0 | 0(0-826.2) | 0 | 0(0-1,282.51) | 0 | 0(0-268.84) |
| **Symptoms, Signs and Ill-Defined Conditions** | 5 | 0.52(0.17-1.21) | 46 | 1.19(0.87-1.58) | 37 | 0.93(0.65-1.28) | 24 | 0.95(0.61-1.42) | 112 | 0.99(0.81-1.19) |
| **Accidents and Adverse Effects** | 13 | 0.85(0.45-1.45) | 46 | 0.75(0.55-1)* | 44 | 0.72(0.52-0.97)* | 45 | 1.01(0.73-1.35) | 148 | 0.81(0.69-0.95)* |
| **Suicide and Self-Inflicted Injury** | 1 | 0.72(0.02-4) | 7 | 1.37(0.55-2.82) | 3 | 0.76(0.16-2.21) | 4 | 1.82(0.5-4.67) | 15 | 1.18(0.66-1.95) |
| **Homicide and Legal Intervention** | 0 | 0(0-8.69) | 2 | 1.38(0.17-4.98) | 1 | 0.9(0.02-5.04) | 0 | 0(0-5.97) | 3 | 0.83(0.17-2.44) |
| **Other Cause of Death** | 103 | 0.92(0.75-1.12) | 366 | 0.79(0.72-0.88)* | 458 | 0.94(0.86-1.03) | 449 | 1.23(1.12-1.35)* | 1,376 | 0.97(0.92-1.02) |

Abbreviation: SMR, standardized mortality ratio. CI: confidence interval. SWD: Marital status at diagnosis is separated, widowed, and divorced.

Values indicate the number of patients with cancer who died from each cause of death.

*P value less than .05

**Supporting Table 26.** Standardized-mortality ratios (SMRs) for each cause of death following Endometrial Cancer diagnosis in patients which ICD codes were grouped as endometrioid subtype

| **Timing of deaths after diagnosis** | | | | | | | | | | |
| --- | --- | --- | --- | --- | --- | --- | --- | --- | --- | --- |
|  | **＜1year** | | **1-5years** | | **5-10years** | | **＞10years** | | **Total** | |
| **Causes of death** | **No.observed** | **SMR(95% CI)** | **No.observed** | **SMR(95% CI)** | **No.observed** | **SMR(95% CI)** | **No.observed** | **SMR(95% CI)** | **No.observed** | **SMR(95% CI)** |
| **All Causes of Death** | 2,365 | 2.45(2.36-2.55)* | 7,566 | 1.76(1.72-1.8)* | 5,539 | 1.20(1.17-1.23)* | 4,174 | 1.16(1.13-1.2)* | 19,644 | 1.46(1.44-1.48)* |
| **Endometrial Cancer deaths** | 1,454 | 133.15(126.4-140.18)* | 3,637 | 76.48(74.01-79)* | 1,057 | 22.80(21.45-24.22)* | 218 | 7.03(6.13-8.03)* | 6,366 | 46.87(45.72-48.03)* |
| **Other cancer causes of deaths** | 213 | 0.88(0.76-1) | 1,207 | 1.17(1.11-1.24)* | 1,047 | 1.04(0.98-1.11) | 678 | 0.99(0.92-1.07) | 3,145 | 1.06(1.03-1.1)* |
| **Non-cancer causes of deaths** | 698 | 0.98(0.91-1.06) | 2,722 | 0.85(0.82-0.88)* | 3,435 | 0.96(0.93-0.99)* | 3,278 | 1.14(1.1-1.18)* | 10,133 | 0.98(0.96-1)* |
| **In situ, benign or unknown behavior neoplasm** | 6 | 1.07(0.39-2.33) | 31 | 1.24(0.84-1.76) | 30 | 1.11(0.75-1.59) | 28 | 1.35(0.9-1.95) | 95 | 1.21(0.98-1.48) |
| **Septicemia** | 32 | 2.10(1.43-2.96)* | 81 | 1.19(0.94-1.48) | 95 | 1.33(1.07-1.62)* | 68 | 1.28(0.99-1.62) | 276 | 1.33(1.17-1.49)* |
| **Other Infectious and Parasitic Diseases including HIV** | 12 | 1.43(0.74-2.49) | 36 | 0.98(0.68-1.35) | 48 | 1.31(0.96-1.73) | 24 | 0.98(0.63-1.45) | 120 | 1.13(0.93-1.35) |
| **Diabetes Mellitus** | 44 | 1.43(1.04-1.92)* | 192 | 1.47(1.27-1.69)* | 180 | 1.39(1.19-1.61)* | 159 | 1.72(1.46-2.01)* | 575 | 1.50(1.38-1.63)* |
| **Alzheimers (ICD-9 and 10 only)** | 14 | 0.40(0.22-0.68)* | 84 | 0.46(0.37-0.57)* | 202 | 0.82(0.71-0.94)* | 265 | 1.09(0.96-1.23) | 565 | 0.80(0.73-0.87)* |
| **Diseases of Heart** | 234 | 1.02(0.89-1.15) | 840 | 0.84(0.78-0.9)* | 1,064 | 1(0.94-1.06) | 942 | 1.12(1.05-1.19)* | 3,080 | 0.98(0.95-1.02) |
| **Hypertension without Heart Disease** | 10 | 0.94(0.45-1.72) | 56 | 1.09(0.82-1.41) | 77 | 1.25(0.99-1.56) | 94 | 1.78(1.44-2.18)* | 237 | 1.34(1.18-1.52)* |
| **Cerebrovascular Diseases** | 75 | 1.22(0.96-1.52) | 229 | 0.85(0.74-0.97)* | 278 | 0.95(0.84-1.07) | 252 | 1.05(0.93-1.19) | 834 | 0.97(0.9-1.04) |
| **Atherosclerosis** | 2 | 0.52(0.06-1.89) | 20 | 1.33(0.81-2.05) | 12 | 0.86(0.44-1.5) | 19 | 1.94(1.17-3.04)* | 53 | 1.24(0.93-1.63) |
| **Aortic Aneurysm and Dissection** | 0 | 0.00(0-0.9)* | 10 | 0.59(0.28-1.09) | 7 | 0.43(0.17-0.88)* | 8 | 0.68(0.29-1.34) | 25 | 0.51(0.33-0.75)* |
| **Other Diseases of Arteries, Arterioles, Capillaries** | 7 | 1.79(0.72-3.68) | 15 | 0.87(0.49-1.44) | 11 | 0.6(0.3-1.08) | 12 | 0.84(0.44-1.47) | 45 | 0.84(0.61-1.12) |
| **Pneumonia and Influenza** | 11 | 0.50(0.25-0.9)* | 70 | 0.73(0.57-0.92)* | 106 | 1.03(0.84-1.25) | 82 | 1.02(0.81-1.27) | 269 | 0.89(0.79-1.01) |
| **Chronic Obstructive Pulmonary Disease and Allied Cond** | 29 | 0.45(0.3-0.65)* | 117 | 0.40(0.33-0.48)* | 174 | 0.55(0.47-0.63)* | 158 | 0.64(0.55-0.75)* | 478 | 0.52(0.47-0.57)* |
| **Stomach and Duodenal Ulcers** | 2 | 1.46(0.18-5.28) | 10 | 1.75(0.84-3.23) | 8 | 1.4(0.6-2.75) | 7 | 1.61(0.65-3.32) | 27 | 1.58(1.04-2.29)* |
| **Chronic Liver Disease and Cirrhosis** | 10 | 0.98(0.47-1.8) | 40 | 0.91(0.65-1.24) | 42 | 1.02(0.74-1.38) | 23 | 0.88(0.56-1.32) | 115 | 0.95(0.78-1.14) |
| **Nephritis, Nephrotic Syndrome and Nephrosis** | 22 | 1.24(0.77-1.87) | 91 | 1.13(0.91-1.39) | 108 | 1.23(1.01-1.49)* | 98 | 1.48(1.2-1.8)* | 319 | 1.26(1.13-1.41)* |
| **Complications of Pregnancy, Childbirth, Puerperium** | 1 | 12.18(0.31-67.86) | 2 | 7.14(0.86-25.78) | 0 | 0(0-22.92) | 0 | 0(0-74.78) | 3 | 5.24(1.08-15.31)* |
| **Congenital Anomalies** | 2 | 1.54(0.19-5.57) | 3 | 0.57(0.12-1.65) | 5 | 1.07(0.35-2.5) | 3 | 1.03(0.21-3.02) | 13 | 0.92(0.49-1.57) |
| **Certain Conditions Originating in Perinatal Period** | 0 | 0(0-986.37) | 0 | 0(0-234.7) | 0 | 0(0-252.32) | 0 | 0(0-417.54) | 0 | 0(0-85.96) |
| **Symptoms, Signs and Ill-Defined Conditions** | 9 | 0.79(0.36-1.51) | 47 | 0.89(0.66-1.19) | 56 | 0.91(0.69-1.18) | 36 | 0.8(0.56-1.1) | 148 | 0.87(0.73-1.02) |
| **Accidents and Adverse Effects** | 17 | 0.68(0.4-1.09) | 81 | 0.72(0.57-0.89)* | 82 | 0.68(0.54-0.85)* | 88 | 0.94(0.75-1.16) | 268 | 0.76(0.67-0.86)* |
| **Suicide and Self-Inflicted Injury** | 3 | 0.69(0.14-2.01) | 7 | 0.40(0.16-0.82)* | 16 | 1.12(0.64-1.81) | 11 | 1.43(0.71-2.56) | 37 | 0.84(0.59-1.16) |
| **Homicide and Legal Intervention** | 0 | 0(0-3.33) | 2 | 0.48(0.06-1.72) | 2 | 0.59(0.07-2.15) | 1 | 0.54(0.01-2.98) | 5 | 0.47(0.15-1.11) |
| **Other Cause of Death** | 135 | 0.95(0.79-1.12) | 547 | 0.80(0.73-0.87)* | 726 | 0.88(0.81-0.94)* | 768 | 1.09(1.01-1.17)* | 2,176 | 0.92(0.88-0.96)* |

Abbreviation: SMR, standardized mortality ratio. CI: confidence interval

Values indicate the number of patients with cancer who died from each cause of death.

*P value less than .05

**Supporting Table 27.** Standardized-mortality ratios (SMRs) for each cause of death following Endometrial Cancer diagnosis in patients which ICD codes were grouped as non-endometrioid subtype

| **Timing of deaths after diagnosis** | | | | | | | | | | |
| --- | --- | --- | --- | --- | --- | --- | --- | --- | --- | --- |
|  | **＜1year** | | **1-5years** | | **5-10years** | | **＞10years** | | **Total** | |
| **Causes of death** | **No.observed** | **SMR(95% CI)** | **No.observed** | **SMR(95% CI)** | **No.observed** | **SMR(95% CI)** | **No.observed** | **SMR(95% CI)** | **No.observed** | **SMR(95% CI)** |
| **All Causes of Death** | 2,789 | 6.11(5.89-6.34)* | 5,667 | 3.34(3.25-3.43)* | 2,339 | 1.36(1.3-1.41)* | 1,957 | 1.13(1.08-1.18)* | 12,752 | 2.27(2.23-2.31)* |
| **Endometrial Cancer deaths** | 2,015 | 425.26(406.9-444.25)* | 3,578 | 205.46(198.79-212.31)* | 526 | 32.18(29.49-35.05)* | 88 | 6.16(4.94-7.59)* | 6,207 | 117.60(114.69-120.56)* |
| **Other cancer causes of deaths** | 299 | 2.78(2.47-3.11)* | 851 | 2.18(2.04-2.33)* | 471 | 1.28(1.17-1.4)* | 335 | 1.04(0.93-1.16) | 1,956 | 1.65(1.57-1.72)* |
| **Non-cancer causes of deaths** | 475 | 1.38(1.26-1.51)* | 1,238 | 0.96(0.91-1.01) | 1,342 | 1(0.95-1.06) | 1,534 | 1.10(1.05-1.16)* | 4,589 | 1.05(1.02-1.08)* |
| **In situ, benign or unknown behavior neoplasm** | 8 | 3.03(1.31-5.97)* | 18 | 1.83(1.08-2.88)* | 10 | 0.99(0.47-1.81) | 11 | 1.1(0.55-1.96) | 47 | 1.44(1.06-1.91)* |
| **Septicemia** | 17 | 2.33(1.36-3.73)* | 29 | 1.08(0.72-1.54) | 28 | 1.04(0.69-1.51) | 30 | 1.17(0.79-1.68) | 104 | 1.2(0.98-1.45) |
| **Other Infectious and Parasitic Diseases including HIV** | 4 | 1.11(0.3-2.85) | 15 | 1.09(0.61-1.8) | 17 | 1.24(0.72-1.98) | 23 | 1.94(1.23-2.92)* | 59 | 1.38(1.05-1.78)* |
| **Diabetes Mellitus** | 25 | 1.67(1.08-2.47)* | 55 | 1.04(0.78-1.35) | 68 | 1.39(1.08-1.76)* | 73 | 1.65(1.3-2.08)* | 221 | 1.37(1.2-1.57)* |
| **Alzheimers (ICD-9 and 10 only)** | 10 | 0.58(0.28-1.06) | 39 | 0.53(0.37-0.72)* | 80 | 0.87(0.69-1.09) | 139 | 1.17(0.99-1.38) | 268 | 0.89(0.78-1) |
| **Diseases of Heart** | 182 | 1.56(1.34-1.81)* | 401 | 0.96(0.87-1.06) | 429 | 1.05(0.95-1.15) | 426 | 1.04(0.94-1.14) | 1,438 | 1.06(1.01-1.12)* |
| **Hypertension without Heart Disease** | 6 | 1.11(0.41-2.42) | 26 | 1.23(0.8-1.8) | 27 | 1.16(0.77-1.69) | 29 | 1.13(0.76-1.62) | 88 | 1.17(0.94-1.44) |
| **Cerebrovascular Diseases** | 28 | 0.88(0.59-1.28) | 99 | 0.87(0.71-1.06) | 96 | 0.86(0.69-1.05) | 142 | 1.22(1.03-1.44)* | 365 | 0.98(0.88-1.08) |
| **Atherosclerosis** | 4 | 1.9(0.52-4.86) | 5 | 0.73(0.24-1.7) | 5 | 0.87(0.28-2.04) | 4 | 0.82(0.22-2.09) | 18 | 0.92(0.54-1.45) |
| **Aortic Aneurysm and Dissection** | 2 | 0.98(0.12-3.56) | 5 | 0.71(0.23-1.65) | 3 | 0.47(0.1-1.38) | 4 | 0.7(0.19-1.8) | 14 | 0.66(0.36-1.11) |
| **Other Diseases of Arteries, Arterioles, Capillaries** | 1 | 0.51(0.01-2.83) | 7 | 0.98(0.39-2.02) | 6 | 0.86(0.32-1.88) | 3 | 0.43(0.09-1.27) | 17 | 0.74(0.43-1.19) |
| **Pneumonia and Influenza** | 15 | 1.35(0.76-2.23) | 40 | 0.99(0.71-1.35) | 42 | 1.06(0.77-1.44) | 41 | 1.05(0.75-1.42) | 138 | 1.06(0.89-1.25) |
| **Chronic Obstructive Pulmonary Disease and Allied Cond** | 22 | 0.76(0.48-1.16) | 54 | 0.49(0.37-0.63)* | 76 | 0.65(0.51-0.82)* | 83 | 0.71(0.57-0.88)* | 235 | 0.63(0.55-0.72)* |
| **Stomach and Duodenal Ulcers** | 0 | 0(0-5.46) | 3 | 1.29(0.27-3.76) | 3 | 1.4(0.29-4.08) | 1 | 0.48(0.01-2.69) | 7 | 0.97(0.39-1.99) |
| **Chronic Liver Disease and Cirrhosis** | 8 | 2.03(0.87-3.99) | 16 | 1.09(0.62-1.77) | 14 | 1.03(0.56-1.72) | 14 | 1.19(0.65-2) | 52 | 1.18(0.88-1.55) |
| **Nephritis, Nephrotic Syndrome and Nephrosis** | 17 | 1.93(1.12-3.08)* | 48 | 1.45(1.07-1.93)* | 38 | 1.12(0.8-1.54) | 43 | 1.34(0.97-1.8) | 146 | 1.35(1.14-1.59)* |
| **Complications of Pregnancy, Childbirth, Puerperium** | 0 | 0(0-176.4) | 0 | 0(0-50.7) | 1 | 18.76(0.47-104.53) | 0 | 0(0-151.2) | 1 | 5.84(0.15-32.51) |
| **Congenital Anomalies** | 0 | 0(0-7.13) | 1 | 0.55(0.01-3.04) | 1 | 0.63(0.02-3.51) | 3 | 2.26(0.47-6.62) | 5 | 0.95(0.31-2.22) |
| **Certain Conditions Originating in Perinatal Period** | 0 | 0(0-2,843.40) | 0 | 0(0-820.11) | 0 | 0(0-836.48) | 0 | 0(0-929.73) | 0 | 0(0-260.27) |
| **Symptoms, Signs and Ill-Defined Conditions** | 7 | 1.3(0.52-2.68) | 22 | 1.06(0.66-1.6) | 26 | 1.09(0.71-1.59) | 17 | 0.76(0.44-1.22) | 72 | 0.99(0.78-1.25) |
| **Accidents and Adverse Effects** | 12 | 1.15(0.59-2) | 29 | 0.71(0.48-1.02) | 36 | 0.85(0.59-1.17) | 29 | 0.65(0.44-0.94)* | 106 | 0.77(0.63-0.93)* |
| **Suicide and Self-Inflicted Injury** | 0 | 0(0-2.56) | 2 | 0.38(0.05-1.37) | 1 | 0.22(0.01-1.22) | 2 | 0.6(0.07-2.15) | 5 | 0.34(0.11-0.8)* |
| **Homicide and Legal Intervention** | 0 | 0(0-8.55) | 2 | 1.39(0.17-5.03) | 1 | 0.86(0.02-4.78) | 2 | 2.36(0.29-8.52) | 5 | 1.29(0.42-3.01) |
| **Other Cause of Death** | 85 | 1.27(1.02-1.58)* | 244 | 0.92(0.81-1.04) | 288 | 0.95(0.84-1.07) | 365 | 1.07(0.97-1.19) | 982 | 1.01(0.94-1.07) |

Abbreviation: SMR, standardized mortality ratio. CI: confidence interval

Values indicate the number of patients with cancer who died from each cause of death.

*P value less than .05

**Supporting Table 28.** Standardized-mortality ratios (SMRs) for each cause of death following Endometrial Cancer diagnosis in patients which ICD codes were grouped as sarcoma subtype

| **Timing of deaths after diagnosis** | | | | | | | | | | |
| --- | --- | --- | --- | --- | --- | --- | --- | --- | --- | --- |
|  | **＜1year** | | **1-5years** | | **5-10years** | | **＞10years** | | **Total** | |
| **Causes of death** | **No.observed** | **SMR(95% CI)** | **No.observed** | **SMR(95% CI)** | **No.observed** | **SMR(95% CI)** | **No.observed** | **SMR(95% CI)** | **No.observed** | **SMR(95% CI)** |
| **All Causes of Death** | 1,091 | 16.91(15.92-17.94)* | 1,282 | 7.49(7.08-7.91)* | 253 | 1.85(1.63-2.09)* | 133 | 1.45(1.21-1.72)* | 2,759 | 5.94(5.71-6.17)* |
| **Endometrial Cancer deaths** | 856 | 1,170.18(1,093.09-1,251.27)* | 915 | 461.39(431.97-492.28)* | 93 | 63.46(51.22-77.74)* | 15 | 16.27(9.11-26.84)* | 1,879 | 368.28(351.81-385.31)* |
| **Other cancer causes of deaths** | 135 | 9.03(7.57-10.69)* | 207 | 5.26(4.56-6.02)* | 55 | 1.88(1.42-2.45)* | 21 | 1.17(0.73-1.8) | 418 | 4.12(3.73-4.53)* |
| **Non-cancer causes of deaths** | 100 | 2.05(1.67-2.49)* | 160 | 1.23(1.05-1.44)* | 105 | 0.99(0.81-1.2) | 97 | 1.33(1.08-1.62)* | 462 | 1.29(1.18-1.41)* |
| **In situ, benign or unknown behavior neoplasm** | 2 | 5.45(0.66-19.68) | 3 | 3.07(0.63-8.96) | 4 | 5.08(1.38-13)* | 1 | 1.96(0.05-10.93) | 10 | 3.78(1.81-6.96)* |
| **Septicemia** | 6 | 5.50(2.02-11.98)* | 6 | 2.1(0.77-4.57) | 5 | 2.26(0.73-5.27) | 3 | 2.11(0.43-6.16) | 20 | 2.64(1.61-4.07)* |
| **Other Infectious and Parasitic Diseases including HIV** | 2 | 3.51(0.42-12.67) | 3 | 1.96(0.4-5.72) | 2 | 1.74(0.21-6.3) | 0 | 0(0-5.41) | 7 | 1.78(0.72-3.67) |
| **Diabetes Mellitus** | 6 | 2.73(1-5.93)* | 9 | 1.62(0.74-3.07) | 6 | 1.45(0.53-3.16) | 1 | 0.39(0.01-2.15) | 22 | 1.52(0.95-2.3) |
| **Alzheimers (ICD-9 and 10 only)** | 2 | 0.79(0.1-2.84) | 3 | 0.4(0.08-1.17) | 2 | 0.27(0.03-0.99)* | 10 | 1.7(0.81-3.13) | 17 | 0.73(0.43-1.17) |
| **Diseases of Heart** | 29 | 1.81(1.21-2.6)* | 45 | 1.11(0.81-1.48) | 39 | 1.23(0.87-1.68) | 26 | 1.22(0.79-1.78) | 139 | 1.27(1.06-1.5)* |
| **Hypertension without Heart Disease** | 1 | 1.18(0.03-6.55) | 2 | 0.87(0.11-3.15) | 2 | 1.02(0.12-3.68) | 1 | 0.7(0.02-3.92) | 6 | 0.92(0.34-2) |
| **Cerebrovascular Diseases** | 5 | 1.15(0.37-2.69) | 10 | 0.9(0.43-1.66) | 4 | 0.45(0.12-1.16) | 5 | 0.82(0.27-1.92) | 24 | 0.79(0.51-1.18) |
| **Atherosclerosis** | 0 | 0(0-14.35) | 2 | 3.48(0.42-12.59) | 0 | 0(0-9.45) | 0 | 0(0-15.65) | 2 | 1.37(0.17-4.96) |
| **Aortic Aneurysm and Dissection** | 0 | 0(0-13.97) | 1 | 1.52(0.04-8.5) | 0 | 0(0-7.77) | 0 | 0(0-12.8) | 1 | 0.59(0.02-3.31) |
| **Other Diseases of Arteries, Arterioles, Capillaries** | 2 | 7.26(0.88-26.21) | 0 | 0(0-5.26) | 1 | 1.83(0.05-10.22) | 2 | 5.41(0.65-19.53) | 5 | 2.64(0.86-6.17) |
| **Pneumonia and Influenza** | 2 | 1.32(0.16-4.79) | 7 | 1.82(0.73-3.74) | 2 | 0.66(0.08-2.38) | 6 | 2.99(1.1-6.5)* | 17 | 1.63(0.95-2.61) |
| **Chronic Obstructive Pulmonary Disease and Allied Cond** | 4 | 1.02(0.28-2.61) | 6 | 0.55(0.2-1.2) | 3 | 0.34(0.07-0.99)* | 7 | 1.21(0.49-2.5) | 20 | 0.68(0.42-1.05) |
| **Stomach and Duodenal Ulcers** | 1 | 11.46(0.29-63.83) | 0 | 0(0-16.73) | 0 | 0(0-22.09) | 0 | 0(0-33.82) | 1 | 1.71(0.04-9.54) |
| **Chronic Liver Disease and Cirrhosis** | 0 | 0(0-6.59) | 1 | 0.63(0.02-3.5) | 0 | 0(0-3.04) | 2 | 2.55(0.31-9.21) | 3 | 0.72(0.15-2.11) |
| **Nephritis, Nephrotic Syndrome and Nephrosis** | 1 | 0.74(0.02-4.11) | 10 | 2.82(1.35-5.19)* | 5 | 1.79(0.58-4.17) | 4 | 2.23(0.61-5.7) | 20 | 2.11(1.29-3.25)* |
| **Complications of Pregnancy, Childbirth, Puerperium** | 0 | 0(0-745.04) | 0 | 0(0-244.57) | 0 | 0(0-372.95) | 0 | 0(0-875) | 0 | 0(0-108.05) |
| **Congenital Anomalies** | 0 | 0(0-52.12) | 0 | 0(0-19.07) | 0 | 0(0-25.96) | 0 | 0(0-40.07) | 0 | 0(0-7.4) |
| **Certain Conditions Originating in Perinatal Period** | 0 | 0(0-18,505.02) | 0 | 0(0-6,010.04) | 0 | 0(0-7,553.22) | 0 | 0(0-10,839.58) | 0 | 0(0-2,246.81) |
| **Symptoms, Signs and Ill-Defined Conditions** | 4 | 5.01(1.37-12.84)* | 3 | 1.42(0.29-4.15) | 3 | 1.7(0.35-4.96) | 1 | 0.89(0.02-4.93) | 11 | 1.89(0.95-3.39) |
| **Accidents and Adverse Effects** | 0 | 0(0-2.39) | 2 | 0.45(0.05-1.62) | 3 | 0.82(0.17-2.39) | 2 | 0.8(0.1-2.88) | 7 | 0.57(0.23-1.18) |
| **Suicide and Self-Inflicted Injury** | 0 | 0(0-17.65) | 1 | 1.6(0.04-8.91) | 0 | 0(0-8.03) | 0 | 0(0-13.35) | 1 | 0.64(0.02-3.55) |
| **Homicide and Legal Intervention** | 0 | 0(0-52.79) | 0 | 0(0-19.88) | 0 | 0(0-29.67) | 0 | 0(0-53.15) | 0 | 0(0-8.21) |
| **Other Cause of Death** | 22 | 2.23(1.39-3.37)* | 26 | 0.94(0.61-1.37) | 19 | 0.78(0.47-1.21) | 23 | 1.31(0.83-1.96) | 90 | 1.13(0.91-1.39) |

Abbreviation: SMR, standardized mortality ratio. CI: confidence interval

Values indicate the number of patients with cancer who died from each cause of death.

*P value less than .05
